# Exoplanetary Atmospheres—Chemistry, Formation Conditions, and Habitability

**DOI:** 10.1007/s11214-016-0254-3

**Published:** 2016-05-12

**Authors:** Nikku Madhusudhan, Marcelino Agúndez, Julianne I Moses, Yongyun Hu

**Affiliations:** 1 Institute of Astronomy, University of Cambridge, Madingley Road, Cambridge CB3 0HA, UK; 2 Instituto de Ciencia de Materiales de Madrid, CSIC, C/Sor Juana Inés de la Cruz 3, 28049 Cantoblanco, Spain, marcelino.agundez@icmm.csic.es; 3 Space Science Institute, 4750 Walnut Street, Suite 205, Boulder, CO 80301, USA, jmoses@SpaceScience.org; 4 Laboratory for Climate and Ocean-Atmosphere Sciences, Department of Atmospheric and Oceanic Sciences, School of Physics, Peking University, Beijing 100871, China, yyhu@pku.edu.cn

**Keywords:** Exoplanets, Exoplanetary atmospheres, Atmospheric chemistry, Planet formation, Habitability

## Abstract

Characterizing the atmospheres of extrasolar planets is the new frontier in exoplanetary science. The last two decades of exoplanet discoveries have revealed that exoplanets are very common and extremely diverse in their orbital and bulk properties. We now enter a new era as we begin to investigate the chemical diversity of exoplanets, their atmospheric and interior processes, and their formation conditions. Recent developments in the field have led to unprecedented advancements in our understanding of atmospheric chemistry of exoplanets and the implications for their formation conditions. We review these developments in the present work. We review in detail the theory of atmospheric chemistry in all classes of exoplanets discovered to date, from highly irradiated gas giants, ice giants, and super-Earths, to directly imaged giant planets at large orbital separations. We then review the observational detections of chemical species in exoplanetary atmospheres of these various types using different methods, including transit spectroscopy, Doppler spectroscopy, and direct imaging. In addition to chemical detections, we discuss the advances in determining chemical abundances in these atmospheres and how such abundances are being used to constrain exoplanetary formation conditions and migration mechanisms. Finally, we review recent theoretical work on the atmospheres of habitable exoplanets, followed by a discussion of future outlook of the field.

## 1 Introduction

The study of extrasolar planets (or ‘exoplanets’) is one of the most dynamic frontiers of modern astronomy. A few thousand exoplanets are now known. The numerous exoplanet discoveries via different methods have shown that exoplanetary systems are very common and are extremely diverse in their macroscopic properties. Recent statistics from observational surveys are suggesting that almost every star in the solar neighborhood hosts at least one planet around it, and that terrestrial-size exoplanets are amongst the most numerous (Howard et al. 2012; Fressin et al. 2013). The exoplanets detected to date span a diverse range in masses, radii, temperatures, and orbital parameters (orbital periods, separations, eccentricities, inclinations, etc.). The extreme diversity of these macroscopic properties of exoplanets has for the first time placed the solar system in cosmic context, thereby opening a plethora of new questions with far reaching implications.

We are now entering a new era in exoplanetary science. Going beyond planet detections, the field is now moving towards detailed characterization of exoplanetary atmospheres through spectroscopic observations. Encoded within a spectrum of an exoplanet is information about the chemical composition and manifold physical processes in its atmosphere. State-of-the-art observations are now beginning to provide both the high sensitivity and long spectral baseline required to place detailed constraints on the various physicochemical properties of exoplanetary atmospheres. Such observations in recent years are already providing good constraints on the chemical compositions of exoplanetary atmospheres. The most observed exoplanets to date are hot giant planets whose large scale-heights and high temperatures (~800–3000 K) make them particularly conducive to atmospheric observations. Several prominent molecules of carbon and oxygen and several atomic species (e.g. Na, K) are expected to be abundant and observable in these atmospheres, making giant exoplanets rich laboratories for understanding atmospheric chemistry. The atmospheric chemical compositions are in turn beginning to provide the first insights into the possible formation conditions and migration mechanisms of exoplanets.

Atmospheric observations have been reported for a variety of exoplanets detected via transits, direct imaging, as well as the radial velocity method. Spitzer observations have been obtained for about 20 transiting exoplanets in at least four photometric bands (3.6, 4.5, 5.8, 8 μm) and about 50 in two (3.6 and 4.5 μm), primarily for close-in hot Jupiters (Madhusudhan et al. 2014c). Photometric observations have also been obtained in the near infrared using large ground-based telescopes. Recently, high S/N near-infrared transit spectroscopy has become possible thanks to the HST Wide Field Camera 3 (WFC3) leading to the first high-confidence detections of H_2_O in several transiting exoplanets (e.g. Deming et al. 2013). Multi-wavelength datasets are also providing the long spectral baseline and high precision required to derive joint constraints on the chemical compositions and temperature profiles of exoplanetary atmospheres. Over 400 HST orbits have been recently allocated for high S/N spectra of over a dozen transiting exoplanets using the HST instruments in the visible and near-infrared. High resolution spectroscopic observations have also been reported for several directly imaged planets in the near-infrared, and dedicated surveys are expected to pursue the same for dozens more. Finally, it has also now become possible to detect molecules in the atmospheres of close-in planets using very high resolution (*R* = 10^5^) Doppler spectroscopy (Snellen et al. 2010). These observational advancements have led to the detection of chemical species in a sizable ensemble of planets and have motivated rapid progress in our theoretical understanding of atmospheric chemistry in exoplanets.

In the present work, we review the latest developments in our understanding of atmospheric chemistry of exoplanets. We begin with a brief introduction to observational methods for exoplanet detection and atmospheric characterization in Sect. 2. We then discuss the theoretical developments in our understanding of atmospheric chemistry in exoplanets (in Sect. 3), with particular emphasis on large gas giant planets (in Sect. 4) as well as ice giants and super-Earths (in Sect. 5). We then review, in Sect. 6, observational inferences of chemical species in exoplanetary atmospheres of various class using different observational methods. In Sect. 7, we review developments towards a theoretical framework for using atmospheric chemical abundances to constrain exoplanetary formation conditions and migration pathways. In Sect. 8, we discuss theoretical developments in our expectations for the habitability of terrestrial exoplanets. We conclude with a discussion of the future outlook for the field.

## 2 Observational Methods

The thousands of exoplanets known to date have been detected via a variety of observational methods (see Fischer et al. 2014, for a review). By far the majority of the planets have been detected using the transit method and the radial velocity method, followed by some detections using various other methods such as pulsar timing, gravitational microlensing, and direct imaging. On the other hand, observations of exoplanetary atmospheres present much more stringent requirements on the instrumental capabilities than planet detection. A combination of significantly higher sensitivity and higher spectral resolution are required for reliable atmospheric detections. Amongst the various planet finding methods, those amenable for atmospheric characterization are the transit method, direct imaging, and radial velocity method, with the transit method being the most successful to date. A detailed review of all these observational methods for atmospheric characterization can be found in Madhusudhan et al. (2014c). Here we briefly review the key attributes of each method.

One of the most successful methods for observing exoplanetary atmospheres has been the transit method. When the planet transits in front of the host star, part of the star light traverses through the day-night terminator region of the planetary atmosphere before reaching the observer. The resulting ‘transmission spectrum’, obtained by subtracting the in-transit spectrum from the out-of-transit spectrum, contains absorption features imprinted on the starlight by chemical species in the planetary atmosphere. Therefore, transmission spectra probe the chemical composition and temperature structure at the day-night terminator of the planet. On the other hand, when the planet is at full phase before being occulted by the star (i.e. ‘secondary eclipse’) the thermal emission and reflection spectrum from the planet is observed along with the stellar spectrum which can be subtracted out later; during secondary eclipse only the stellar spectrum is observed. Thus, secondary eclipse spectra probe composition and thermal structure of the dayside atmosphere of the planet. Spectra for exoplanets have been obtained in both transmission and at secondary eclipse, and in a wide range of wavelengths. While transmission spectra have been obtained from UV to mid-infrared wavelengths, thermal emission spectra have been obtained predominantly in the near to mid infrared. Besides the transit method, atmospheric observations have also been obtained from direct imaging of planets as well as by high-resolution Doppler spectroscopy, which are both discussed briefly in their respective sections below.

## 3 Factors Influencing Atmospheric Chemistry of Exoplanets

The advent of observational techniques able to characterize atmospheres of extrasolar planets has been a major breakthrough in astronomy. The discovery of a great diversity of exoplanets, many of them with no analogue in the solar system, suggests that there is an exoplanet zoo out there with a continuum of possible sizes (from Jupiter-sized planets to sub-Earth bodies), atmospheric temperatures (from thousands of degrees Kelvin down to a few tens), and elemental compositions (from H/He-dominated atmospheres with a solar composition to atmospheres dominated by heavy molecules such as H_2_O, CO_2_, or N_2_). This diversity poses a great challenge to theorists who seek an understanding of the variety of existing planetary climates.

A theoretical approach to the chemistry of exoplanet atmospheres must start by posing a question of the following type. How can we infer the chemical composition of a planetary atmosphere from a reduced set of parameters related to the planet? Of course it is first necessary to identify such parameters and to understand how each of them affect the atmospheric chemistry. Some of these parameters can be obtained in a straightforward way from observations, although there are some others for which we can just have guesses, albeit in some cases with formidable uncertainties. Let’s have a look to each of these parameters.

### Gravity

This parameter follows directly from the mass and radius of the planet, which in turn can be derived from radial velocity and transit techniques, respectively. Whether or not a planet can retain a substantial amount of atmosphere is largely related to its gravity and the X-ray and EUV flux of the host star (see e.g. chapter on protoatmospheres in Massol et al. 2016, this issue). Moreover, depending on the mass and radius of the planet, light elements such as hydrogen and helium may or not escape from the planet, with strong implications for the elemental composition of the atmosphere. The gravity is also important in that, together with the mean mass of particles and temperature, it sets the scale height of the atmosphere, i.e., how compact or extended the atmosphere is.

### Elemental composition

The relative abundances of the different elements is one of the most important aspects that determine the atmospheric composition, although unfortunately these are rather difficult to infer from observations. Based on the study of solar system planets and on existing theories of planet formation (Lissauer 1993; Hubbard et al. 2002; Goldreich et al. 2004; Alibert et al. 2005; Papaloizou and Terquem 2006; Blum and Wurm 2008; Chiang and Youdin 2010; Morbidelli et al. 2012), it is expected that giant planets will from by core accretion, becoming massive enough at an early stage of planet formation to efficiently capture the nebular gas, and thus will retain a thick H/He-dominated atmosphere (see e.g. chapter on protoatmospheres in Massol et al. 2016, this issue). Terrestrial planets, on the other hand, will not become massive enough to efficiently accrete or retain H_2_/He from the nebular gas, and their atmospheres, whether thick or thin, will depend on their mass and evolutionary history (e.g., orbital evolution, stellar/disk evolution, impact history, interior outgassing, atmospheric escape, climate evolution, surface-atmosphere interactions, magnetospheric interactions). Terrestrial-planet atmospheres are expected to be typically dominated by secondary products such as H_2_O, CO_2_, CO, N_2_, Ne, Ar, Kr, SO_2_, SiO_2_ that were outgassed from the solid planetesimals that formed the planets or that were supplied by later impacts by solid bodies (see e.g. chapter on planet formation in Bai et al. 2016, this issue). In between giant and terrestrial planets there is a regime of super-Earths/sub-Neptunes whose elemental composition is largely unknown (e.g., Haghighipour 2013; Fortney et al. 2013; Lopez and Fortney 2014; Marcy et al. 2014; Lee et al. 2014; Rogers 2015). By comparing the outcomes of chemical models and observations, it is in principle possible to put constraints on the elemental composition of the planetary atmosphere (e.g., Madhusudhan et al. 2014c), which in turn can provide clues to the formation and evolution of the planet itself.

### Insolation

The amount of energy received at the top of the atmosphere per unit time and unit area depends essentially on the luminosity of the host star and the orbital distance, two properties that can be relatively well constrained by observations. Of course, the way the stellar luminosity is divided across the electromagnetic spectrum, i.e., the spectral type of the star, has a great importance in affecting atmospheric characteristics. Incoming visible and infrared photons take care of the heating of the atmosphere, especially at pressures greater than ~1 microbar (e.g., Seager and Sasselov 1998; Sudarsky et al. 2003; Barman et al. 2005; Iro et al. 2005; Fortney et al. 2005; Burrows et al. 2006), while ultraviolet photons heat the high-altitude thermosphere and lead to ionization and dissociation of atmospheric constituents and induce photochemistry at a variety of altitudes (e.g., Liang et al. 2003; Yelle 2004; García Muñoz 2007; Zahnle et al. 2009a; Line et al. 2010; Moses et al. 2011; Venot et al. 2012; Agúndez et al. 2012; Hu et al. 2012; Miller-Ricci Kempton et al. 2012; Koskinen et al. 2013; Lavvas et al. 2014; Shaikhislamov et al. 2014; Rimmer and Helling 2015).

### Internal heating

The interiors of planets can provide an important source of heat for the atmosphere, depending on the planet’s age and tidal interactions with the host star or other planets in the system. The internal heating can be estimated from theoretical models of the evolution of the planet interior and its reaction to tidal forces (Guillot 2005; Baraffe et al. 2010; Ogilvie 2014). However, uncertainties in some key magnitudes, such as the dissipation properties of the bulk material of the planet interior and the age of the star (and thus of the planetary system), keep the internal heating poorly constrained in most cases.

A self-consistent model of a planetary atmosphere should in principle be able to describe the physical and chemical state of the atmosphere from the parameters described above. The situation resembles that of stellar atmospheres (e.g., Kurucz 1979; Hauschildt et al. 1999), where models need just three parameters (gravity, metallicity, and effective temperature)—external insolation being not important. Of course, in certain types of planets, parameters or processes different from those listed above, such as, e.g., exchange of matter with the surface in the case of terrestrial planets (e.g., Leconte et al. 2014) or grain formation and resulting opacity effects (e.g., Tsuji et al. 1996; Marley et al. 1996; Allard et al. 1997), may be also of great relevance in establishing the physical and chemical atmospheric properties.

In practice, models of planetary atmospheres lack a full self-consistency and tend to focus on some particular aspects, adopting approximations for some others. On the one hand, there are one-dimensional models that concentrate on solving the radiative transfer and thermal balance in the vertical direction. These models result in a pressure-temperature (*P*-*T*) profile for which the temperature gradient (*dT /dP*) is subadiabatic in the radiative zone of the atmosphere and adiabatic in the deeper convective regions (e.g., McKay et al. 1989; Burrows et al. 1997, 2008; Seager et al. 2005). These radiative-convective equilibrium models usually make some assumptions about the chemical composition of the atmosphere, either adopting a particular one suitable for the modeled planet or assuming that chemical equilibrium holds in the atmosphere, and neglecting any fluid dynamics. For an assumed composition, in addition to the *P*-*T* profiles these non-gray numerical models also calculate the line-by-line thermal emission and reflectance spectra of the planetary atmospheres. Recent studies have also reported analytic and semi-analytic *P*-*T* profiles derived for irradiated planetary atmospheres in radiative equilibrium under the assumptions of gray/non-gray opacities (Hansen 2008; Guillot 2010; Robinson and Catling 2012; Heng et al. 2012; Parmentier and Guillot 2014; Parmentier et al. 2015).

Other type of models, the so-called general circulation models (GCMs), were originally developed to study the climate of the Earth (e.g., Phillips 1956; Mellor and Yamada 1982) and are now routinely used to study atmospheres of other solar system planets (e.g., Forget et al. 1999) as well as exoplanets such as hot Jupiters (e.g., Showman and Guillot 2002; Showman et al. 2009), hot Neptunes (e.g., Lewis et al. 2010), super-Earths (e.g., Castan and Menou 2011) and terrestrial planets (e.g., Showman et al. 2013). These are three-dimensional models which solve the Navier-Stokes equations or a reduced set of “primitive equations” (see e.g. Showman et al. 2010; Heng and Showman 2015) and end up with a three-dimensional view of the circulation and thermal structure of the atmosphere. Some of the limitations of GCMs have to do with the approximations used to deal with the radiative transfer (e.g., Showman et al. 2009) and the chemical processes (Cooper and Showman 2006). Another type of models are those focused on the atmospheric chemistry, in which we concentrate hereafter.

Chemical models of planetary atmospheres aim at describing how the atmospheric constituents are distributed, usually in the vertical direction. To build such a model it is necessary to have information on some basic parameters, among them three of the four aforementioned: (1) *gravity*, (2) *elemental composition*, and (3) *ultraviolet/X-ray irradiation.* It is also necessary to have information on a couple of additional parameters.

### (4) Thermal structure

The spatial distribution of the atmospheric temperature, at least in the vertical direction, can be retrieved from radiative-convective models and GCMs (see above). In the case of these latter models it is possible to get a three-dimensional map of the temperature. The atmospheric temperature has an enormous influence on the chemical composition because depending on whether the atmosphere is hot or cold the major constituents and the type of condensates formed (if any) are completely different.

### (5) Strength of transport processes

An atmosphere is in essence a fluid and therefore a variety of processes such as advection, diffusion, and turbulent motions can occur at different scales, having as consequence the transport and mixing of material between different regions. These processes are an important source of disequilibrium because they can transport molecules that were originally formed in a given location to other atmospheric regions. In particular, the vertical chemical structure can be strongly affected by transport processes, the strength of which is usually parameterized through an eddy diffusion coefficient (*K_zz_* if referred to the vertical direction). It is, however, difficult to obtain realistic estimates of the coefficient *K_zz_*. In the case of solar system planets such as Jupiter and Saturn, if observations are able to determine the abundance of certain species at a given altitude, it is possible to put constraints on the eddy diffusion coefficient (e.g., Atreya 1986). In the case of extrasolar planets, information on the strength of mixing processes must be obtained from theoretical models such as GCMs (e.g., Parmentier et al. 2013).

If one wants to build a chemical model of a planetary atmosphere, these five ingredients (*gravity, elemental composition, ultraviolet/X-ray irradiation, thermal structure*, and *strength of transport processes*) should be in principle enough to provide a good description of the chemical structure of the atmosphere.

The characterization of exoplanet atmospheres through observations has been to date restricted to hot Jupiters, some hot Neptunes, and a few super-Earths, most of them characterized by transit techniques, and to a few young and self-luminous gas giant planets with an orbital distance sufficiently large to be accessible by direct imaging. It is therefore not surprising that theoretical studies of exoplanet atmospheres carried out to date have been to a large extent biased toward these types of extrasolar planets. A general theoretical characterization of habitable-zone exoplanet atmospheres has also been a popular topic, due to the high intrinsic interest in the potential for life (and its detectability) outside of Earth.

## 4 Theory of Atmospheric Chemistry of Gas Giant Exoplanets

**Fig. 1:**
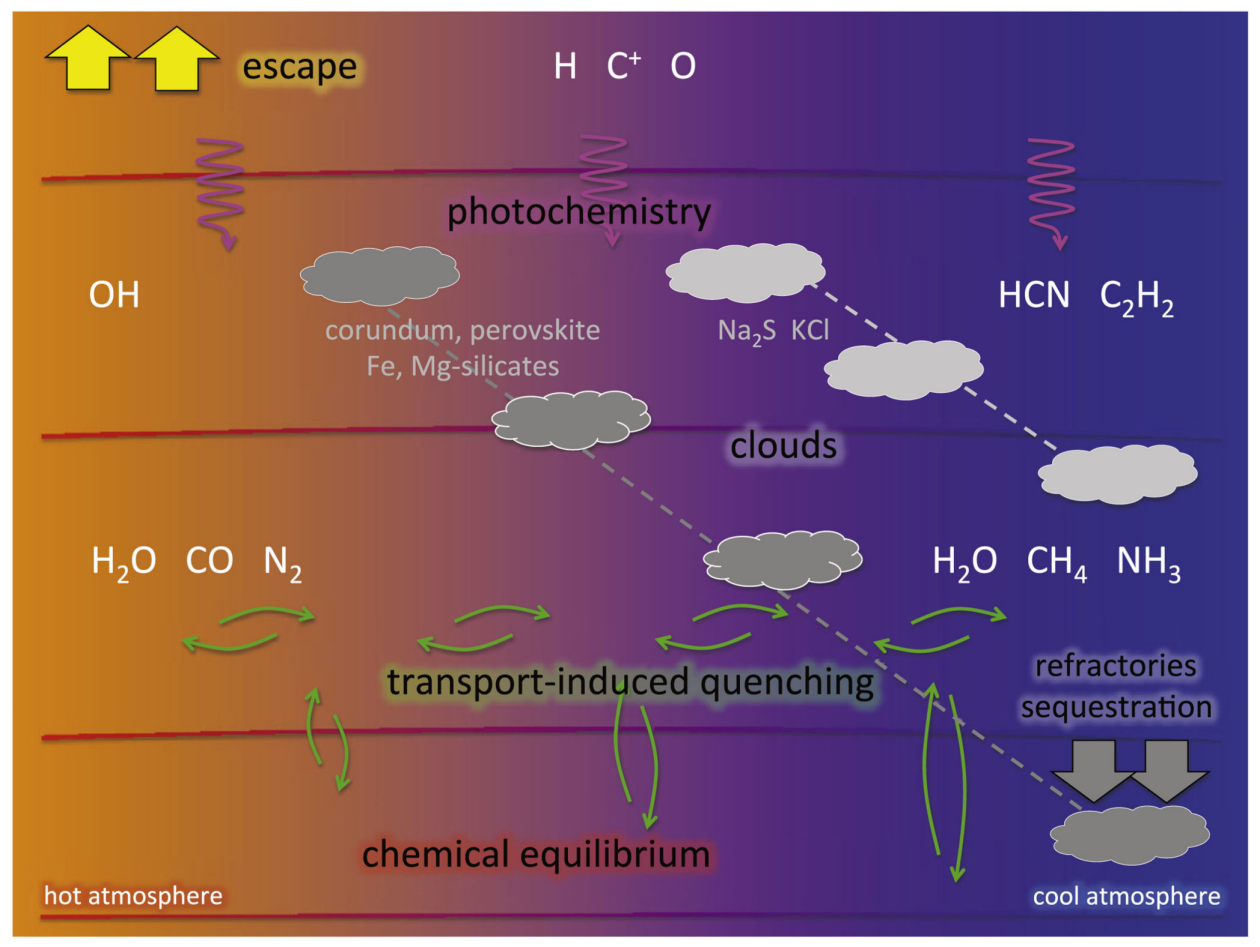
Sketch of the structure and processes at work in H/He-dominated atmospheres. The chemical composition in deep layers is controlled by chemical equilibrium, by transport-induced quenching in upper layers, and by photochemistry in still upper layers. Formation of clouds is an issue of increasing importance as the atmosphere becomes cooler. Escape can also be an important process in highly irradiated atmospheres (i.e., those with hot thermospheres), while sequestration of refractory elements becomes important in the cooler atmospheres

Gas giant exoplanets are expected to have H/He-dominated atmospheres based on their formation mechanism and on the bulk densities inferred from observations (Lissauer and Stevenson 2007; Baraffe et al. 2010). Their chemical composition depends on various parameters, perhaps the most important being the temperature. A sketch of the structure of such an atmosphere is depicted in Fig. 1, where various important processes at work are indicated. The chemical composition is controlled by chemical equilibrium in deep atmospheric layers, by transport-induced quenching in upper layers, and by photochemistry in still upper layers. Cloud formation becomes increasingly important as the atmosphere is cooler. Atmospheric escape can be an important issue for highly irradiated giant planets (e.g., Koskinen et al. 2014), while sequestration of refractory elements plays an important role in cool atmospheres (e.g., Lodders 2010).

### 4.1 Chemical Equilibrium

Under the chemical equilibrium assumption, the chemical composition does only depend on temperature, pressure, and elemental abundances, and can be calculated in a relatively straightforward way by minimizing the Gibbs free energy of the system. Chemical equilibrium can accurately describe the composition of an atmosphere provided it is sufficiently hot and dense to ensure that chemical reactions occur faster than any other process at work (dynamics, interaction with energetic radiation, etc.). This can be the case for atmospheres of cool stars and brown dwarfs, where chemical equilibrium has been routinely applied (Tsuji 1973; Fegley and Lodders 1996; Allard et al. 1996; Burrows and Sharp 1999; Lodders and Fegley 2002), and of hot exoplanets with atmospheric temperatures in excess of ~2000 K (Moses et al. 2011; Venot et al. 2012). In the case of cooler planets, it can still be a good starting point to have a general idea of which could be the main atmospheric constituents.

**Fig. 2:**
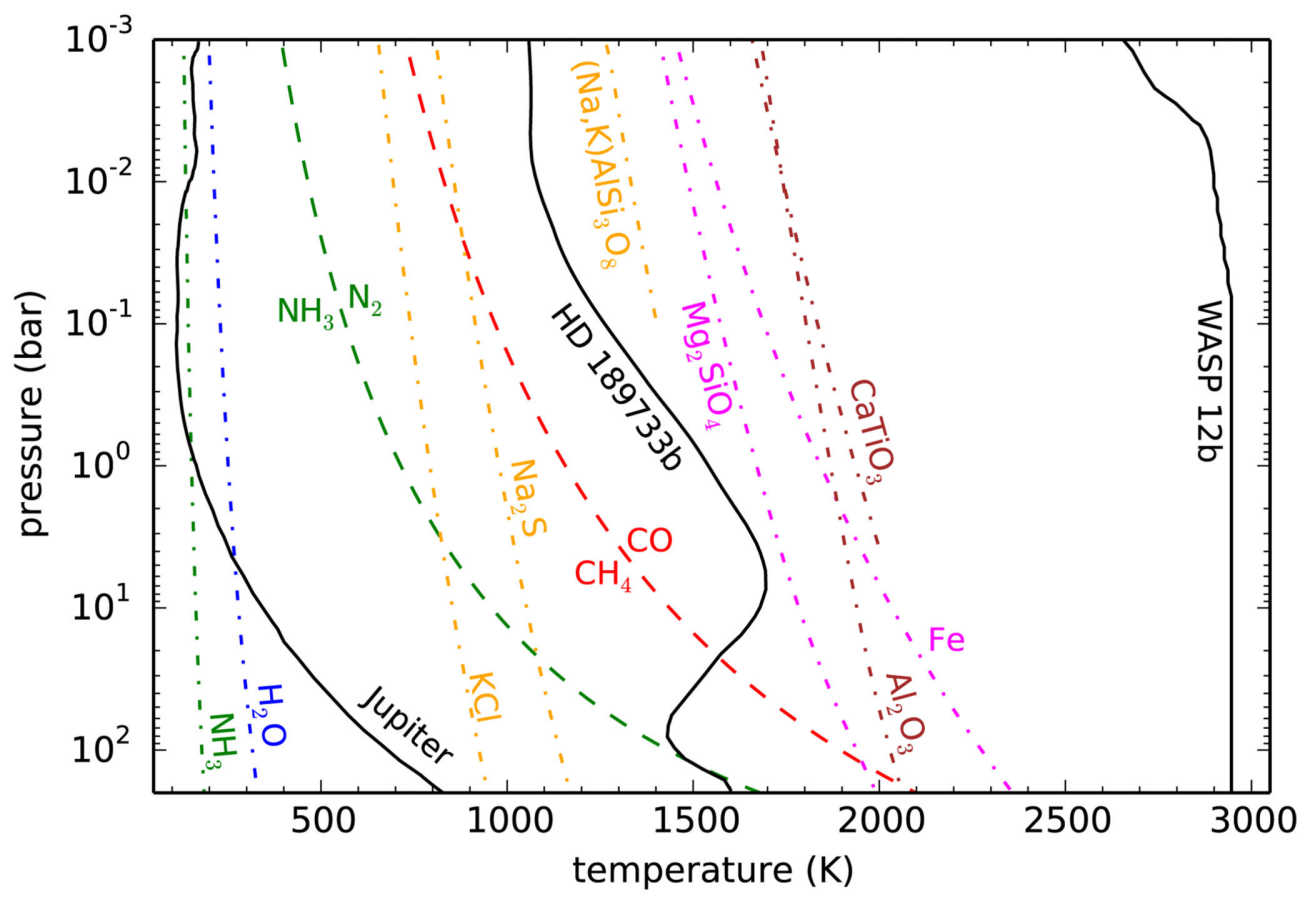
Pressure-temperature profiles for three gas giant planets covering a broad range of atmospheric temperatures: Jupiter (Lindal 1992), the widely studied hot Jupiter HD 189733b (Agúndez et al. 2014a), and the highly irradiated gas giant WASP-12b (Stevenson et al. 2014a). *Dashed lines* delimitate the regions where either CH_4_ or CO (*red curve*) or NH_3_ or N_2_ (*green curve*) are the major carbon or nitrogen reservoirs, respectively. *Dot-dashed lines* indicate the condensation curves for water, ammonia, and various refractory species containing Ca, Ti, Al, Fe, Mg, Na, and K

The atmospheric temperatures of gas giant planets may span over a very broad range, from a few hundreds of kelvins up to some thousands (see Fig. 2). Gas giant planets are expected to have a nearly solar elemental composition, although some elements heavier than helium could be enriched or depleted by a factor of a few relative to the solar composition, as occurs in Jupiter (Mahaffy et al. 2000; Wong et al. 2004). In any case, gas giant atmospheres are expected to be dominated by hydrogen and helium, with heavier elements like oxygen, carbon, and nitrogen being present at a lower level. Figure 2 shows various curves, calculated under chemical equilibrium for a solar elemental composition, which delimitate different regions of interest from a chemical view point. The red dashed curve indicates where CO and CH_4_ have the same abundance. To the right of this curve (at high temperatures/low pressures) we have the region of stability of CO while to the left (at low temperatures/high pressures) methane is the main carbon reservoir. Similarly, in the case of nitrogen chemical equilibrium indicates that to the right of the green dashed curve N_2_ is the main nitrogen reservoir while to the left it is ammonia that dominates. Thus, a first lesson to be learnt from chemical equilibrium is that at high temperatures and/or low pressures CO and N_2_ are the main reservoirs of carbon and nitrogen, respectively, while at low temperatures and/or high pressures the hydrides CH_4_ and NH_3_ dominate. In the case of oxygen, water vapor remains a major reservoir over most of the temperature regime of interest. This molecule locks either the excess of oxygen not locked into CO if the atmosphere resides in the stability region of CO (oxygen has a solar abundance about twice than carbon), or directly locks most of the available oxygen if the atmosphere resides in the stability region of CH_4_. Only at very low temperatures (see blue dot-dashed line) can water vapor be depleted from the gas phase because of condensation, something that can occur also for ammonia, albeit at even lower temperatures (see green dot-dashed line). That is, according to chemical equilibrium, depending on the thermal profile, the atmosphere of a giant planet may be dominated by (apart from H_2_ and He) a mixture of H_2_O/CO/N_2_, H_2_O/CH_4_/N_2_, H_2_O/CH_4_/NH_3_, or a modification of the latter in which first H_2_O, then NH_3_, and finally CH_4_ are progressively removed from the gas phase as the atmosphere gets cooler (e.g., Burrows et al. 1997; Sudarsky et al. 2000; Lodders and Fegley 2002).

### 4.2 Disequilibrium Chemistry Driven by Transport Processes

The atmospheric composition of gas giant exoplanets is governed by chemical equilibrium only in the hottest regions, usually in the deepest, densest regions (or the bulk of the atmosphere for very hot, strongly irradiated planets). In cooler upper regions, when chemical reactions become slower than dynamic processes (advection, diffusion, or turbulence), the bulk composition can be significantly driven out of equilibrium. The transport of material between different regions has as a consequence a redistribution (homogenization) of heat and chemical composition, with a net flow from hot to cooler regions.

The vertical distribution of the atmospheric constituents can be strongly affected by the so-called chemical quenching, in which as material moves upwards chemical reactions become slower, and at a certain level the abundances of the different species are progressively quenched. This phenomenon was first described by Prinn and Barshay (1977) to explain the presence of CO in the troposphere of Jupiter as a result of upward mixing from deeper and hotter levels, where CO is thermochemically stable. In gas giant exoplanets, chemical quenching regulates to a large extent the composition of the layers probed by observations, especially if their pressure-temperature profiles cross the boundaries of stability of the pairs CH_4_/CO or NH_3_/N_2_ (see Fig. 2). A quantitative evaluation of chemical quenching (i.e., the quench level and resulting quenched abundance) requires a good knowledge of both the chemical kinetics of the interconversion scheme of interest and the vertical eddy mixing coefficient.

The main issue when dealing with quenching and chemical conversion schemes is the identification of the elementary reactions involved and the “limiting step”, whose rate controls the kinetics of the overall conversion. The interconversion $\text{CO}⇌{\text{CH}}_{\text{4}}$ is of great importance in cool to moderately warm gas giant planets because exoplanet temperature-pressure profiles often cross the stability boundary between CO and CH_4_ (red dashed curve in Fig. 2). Understanding how the conversion between $\text{CO}⇌{\text{CH}}_{\text{4}}$ proceeds kinetically and which reaction is the rate-limiting step is thus of primordial importance (see Visscher and Moses 2011 for more details). In the same vein, the interconversion ${\text{N}}_{\text{2}}⇌{\text{NH}}_{\text{3}}$ is also of great relevance for the atmospheres of gas giant planets, N_2_ and NH_3_ being the main reservoirs of nitrogen at high and low temperatures (or low and high pressures), respectively. Although there are still important uncertainties in the reaction schemes and the rate-limiting step, the conversion N_2_ → NH_3_ is likely intrinsically slower than the conversion CO → CH_4_, resulting in a deeper quench level for the nitrogen species (see Moses 2014 for more details). The identification of interconversion reaction schemes is interesting in that it permits implementation of simple prescriptions to deal with chemical quenching in high-demanding computational models of atmospheres such as GCMs (Cooper and Showman 2006).

For 1D exoplanetary atmosphere models, the eddy diffusion coefficient must be quantified from 3D theoretical models like GCMs, or constrained from the observations themselves. Although mixing in planetary atmospheres can occur through large-scale advection, atmospheric waves, eddies of a variety of scales, and other transport processes that are not diffusive in a rigorous sense, vertical mixing can typically be well represented by a diffusion equation (e.g. Lindzen 1981; Strobel 1981; Brasseur et al. 1999). A common practice has been to estimate the vertical diffusion coefficient *K_zz_* as the root mean square of the vertical velocity (as extracted from a GCM) times the vertical scale height (Line et al. 2010; Moses et al. 2011). A better approach when 3D circulation models are available, as outlined by Parmentier et al. (2013), is (1) to follow the behavior of passive tracers in a GCM simulation and fit the resulting planet-averaged vertical tracer profiles via a 1D diffusion equation with an effective diffusion coefficient and/or (2) to determine the diffusive flux and resulting *K_zz_* profile that best matches the horizontally averaged vertical flux in the GCMs. These latter methods have been shown to result in *K_zz_* values significantly lower than those obtained by representing *K_zz_* as the root-mean-square vertical velocity times the scale height (Parmentier et al. 2013; Agúndez et al. 2014a). The exact value of the eddy diffusion coefficient, especially at pressures greater than ~0.1 bar, has a direct impact on the location of the quench level of each species (the higher the *K_zz_*, the deeper the quench level) and the abundance at which each species gets quenched in the upper atmosphere. If the quench point falls within the radiative region of the atmosphere, *K_zz_* is expected to vary with the inverse square root of atmospheric pressure near this quench point (Lindzen 1981; Parmentier et al. 2013). If the quench point falls within the deeper convective region of the atmosphere, mixing-length theory and free-convection theory can be used to estimate the magnitude of *K_zz_* near the quench point (Visscher et al. 2010b), or expressions based on laboratory studies of turbulent rotating convection can be developed (Wang et al. 2015). However, the appropriate mixing length to use for the *K_zz_* estimates is not straightforward (e.g., Smith 1998), and observations of quenched species may themselves provide our best means of estimating the magnitude of convective mixing at depth.

**Fig. 3:**
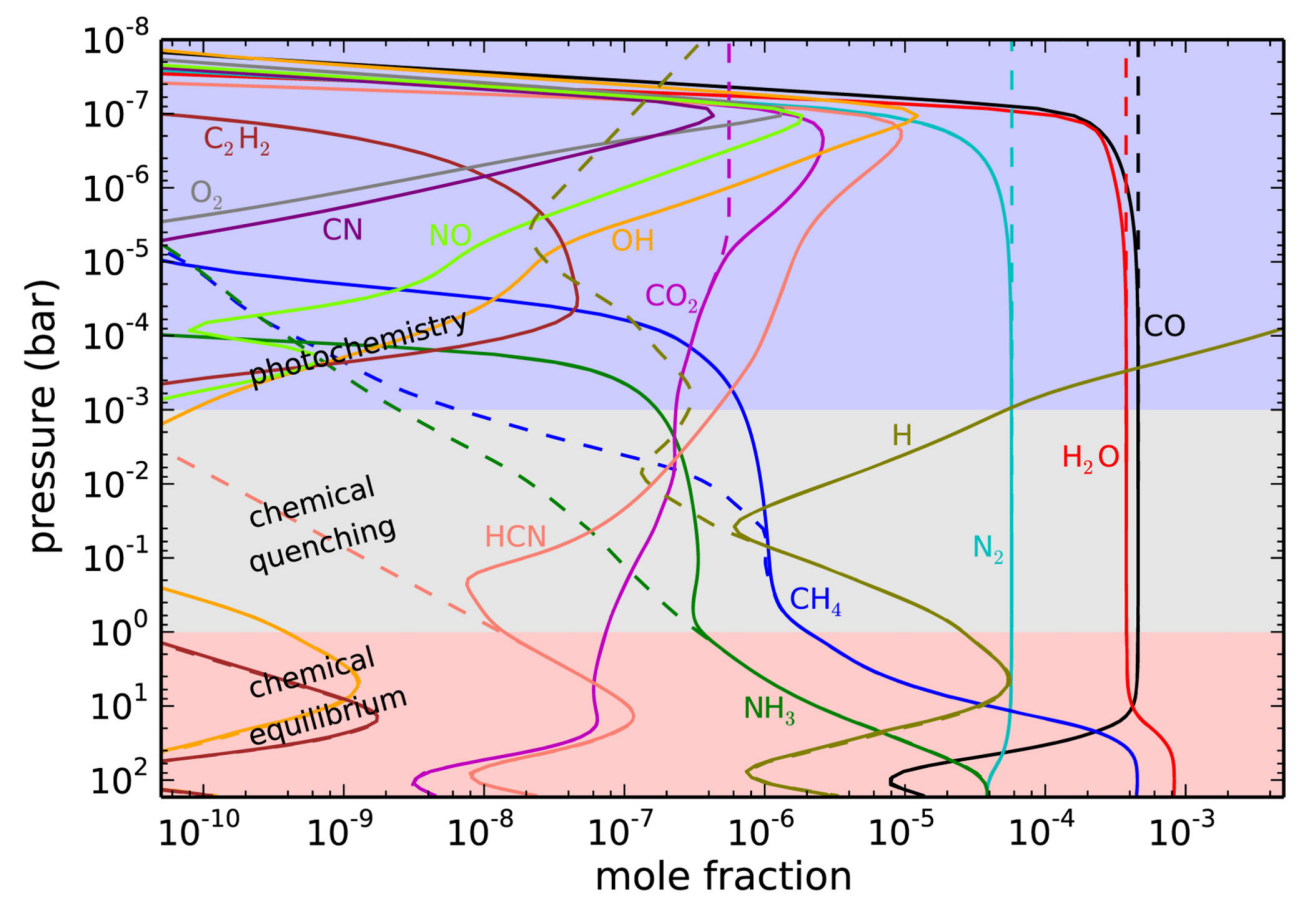
Vertical distribution of abundances at the substellar point of HD 189733b. *Dashed lines* correspond to chemical equilibrium and *solid lines* to a one-dimensional vertical model including thermochemical kinetics and photochemistry (Agúndez et al. 2014a). The atmosphere can be schematically divided into three regions where the composition is controlled by either chemical equilibrium, chemical quenching, or photochemistry

Chemical quenching in the vertical direction of hot Jupiter atmospheres has been studied through timescale arguments (Visscher et al. 2006; Line et al. 2010; Bilger et al. 2013) and using more robust chemical networks (in which endothermic reactions are included and reverse reaction rates are computed from thermochemical grounds) suitable to model high temperature environments (Zahnle et al. 2009a,b; Moses et al. 2011, 2013b; Venot et al. 2012; Kopparapu et al. 2012; Agúndez et al. 2014a; Miguel and Kaltenegger 2014; see also Fig. 3). Most of these studies focus on the atmospheres of the widely observed hot Jupiters HD 189733b and HD 209458b, where it is predicted that, assuming nearly solar elemental abundances, CO and N_2_ are the major reservoirs of carbon and nitrogen, respectively, H_2_O is very abundant (it takes up most of the excess of oxygen not locked into CO), and methane and ammonia are present at a lower level, with CO/CH_4_ and N_2_/NH_3_ abundance ratios in the ranges 50–500 and 5–200, respectively, in HD 189733b, and in the ranges 1000–10000 and 100–1000, respectively, in the hotter HD 209458b (Moses et al. 2011; Venot et al. 2012; Agúndez et al. 2014a; Miguel and Kaltenegger 2014). The abundance of carbon dioxide is found to be somewhat low for atmospheres with an assumed solar elemental composition (see Fig. 3). It is interesting to note that when CO is the dominant carbon component, CO_2_ is much less affected by chemical quenching than many other molecules because the various reactions involved in the interconversion between H_2_O-CO-CO_2_ are rapid enough (Moses et al. 2011; Venot et al. 2012). The chemistry of elements other than C, N, and O, although more difficult to constrain through observations and more uncertain due to a lack of robust reaction rate measurements under appropriate conditions, has been also studied theoretically. It has been pointed out, for example, that chemical quenching leads to PH_3_ becoming a major reservoir of phosphorus in hot Jupiter atmospheres (Visscher et al. 2006).

**Fig. 4:**
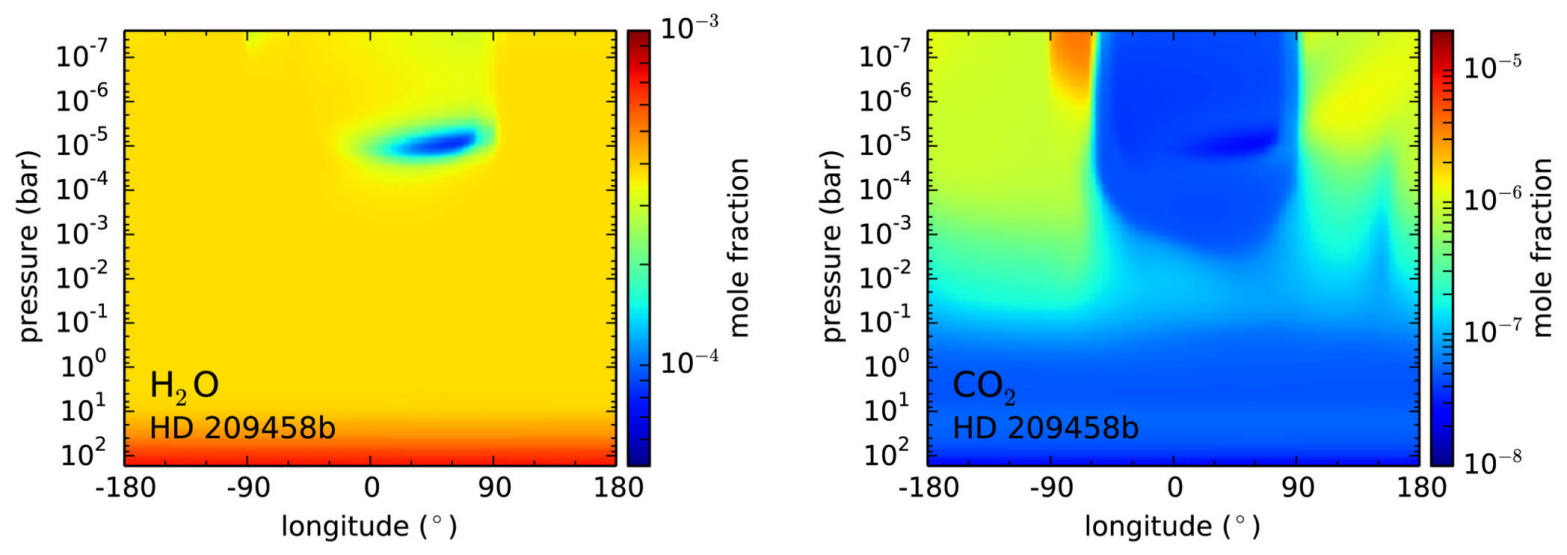
Calculated distributions of H_2_O and CO_2_ as a function of longitude and pressure in the atmosphere of HD 209458b, from Agúndez et al. (2014a). Note that H_2_O maintains a rather homogeneous distribution while CO_2_ experiences important abundance variations with longitude and height

Chemical quenching can also occur horizontally driven by winds that move material from hot to cooler atmospheric regions. In hot Jupiters, which are usually tidally locked (with permanent day and night sides), the uneven heating of the planet may result in important temperature contrasts, and thus in possible variations in the composition, between the day and night sides. On the other hand, circulation dominated by a strong superrotating equatorial jet tends to homogenize both the temperature and chemical composition between the different planetary sides (Showman and Guillot 2002; Cooper and Showman 2006; Knutson et al. 2007). The main effect of horizontal chemical quenching is that molecular abundances are quenched horizontally to values typical of the hottest dayside regions, making the cooler nightside to be highly contaminated by the warmer dayside regions (Cooper and Showman 2006; Agúndez et al. 2012, 2014a). The effect is however more marked for molecules such as H_2_O, CO, and N_2_, which show rather uniform abundances, than for others such as CH_4_, NH_3_, CO_2_, and HCN, which may still show important abundance gradients among the different planetary sides (see Fig. 4). The horizontal homogenization of abundances becomes more important as the atmosphere gets cooler. The distribution of the atmospheric constituents has implications for transit observations, which probe the terminator region, and for phase curves probing the different planetary sides.

### 4.3 Photochemistry

Hot Jupiters are strongly irradiated by their host stars and thus receive a high ultraviolet flux, which is absorbed in the upper atmosphere and induces a more or less rich photochemistry. That is, the parent molecules transported from deep atmospheric regions are photodissociated in upper layers producing radicals that react to form new species. The study of the photochemistry of exoplanet atmospheres has benefited from decades of study of solar system atmospheres such as those of Jupiter (Gladstone et al. 1996), Saturn (Moses et al. 2000), and Titan (Yung et al. 1984; Lara et al. 1996). However, some of the chemical kinetics and photoabsorption cross section data still need to be adapted to the high temperatures of hot Jupiters (Venot et al. 2013). The type of molecules formed by photochemistry depends on the composition of the precursor material transported from deeper layers, which in turn depends on the elemental composition (see Sect. 4.4) and thermal atmospheric structure (see Sects. 4.1 and 4.2). The cooler the planet the larger the extent of photochemistry because, on the one hand, high temperature chemistry counterbalances the action of photochemistry, and on the other, low temperatures favor the presence of CH_4_ and NH_3_, which are more active photochemical precursors than their respective high-temperature counterparts CO and N_2_ (Moses 2014).

There are various species produced by photochemistry in the atmospheres of gas giant planets (see Fig. 3). These atmospheres being hydrogen-rich, a major species produced by photochemistry is atomic hydrogen, which is formed by photolysis and thermal decomposition of H_2_ as well as by catalytic photolysis of H_2_O (Liang et al. 2003). The higher the irradiation of the planet the more abundant becomes H. Another important photochemical species is HCN, which is efficiently formed in the photochemical layer through schemes starting with the photodissociation of CH_4_ and NH_3_ (provided the atmosphere is not too hot to exclude these two hydrides as important constituents), and is able to diffuse to deeper regions (Zahnle et al. 2009a; Moses et al. 2011). The photodissociation of CH_4_ also triggers the formation of larger hydrocarbons such as C_2_H_2_, which may then polymerize to form soots (Zahnle et al. 2009a; Bilger et al. 2013). Another important photochemical product is the radical OH, which results from the photodissociation of water and act as a key intermediate in the synthesis of other O-bearing molecules such as O_2_ and NO (Moses et al. 2011). As concerns sulfur compounds, the major photochemical products of the dominant equilibrium sulfur species, H_2_S, are expected to be S, HS, S_2_, SO, and SO_2_ (Zahnle et al. 2009b). Lavvas et al. (2014) show that for planets hot enough that refractory elements are not tied up in condensates, atomic neutrals and ions such as Mg, Mg^+^, Fe, Fe^+^, Ca, Ca^+^, Na, Na^+^, K, K^+^, Si^+^, Al^+^ could be important photochemical and equilibrium species, although some dominant equilibrium molecular forms (e.g., SiO, SiS, AlOH, FeH) can also survive in the photosphere on hot planets (see also Visscher et al. 2010a), and molecular species like NaCl and KCl may form from both photochemistry and thermochemistry.

On highly irradiated hot Jupiters, most photochemical products are confined to relatively high altitudes (e.g., above ~0.1 mbar, see Fig. 3). Therefore, photochemical products are likely to have a limited impact on the infrared emission spectrum of the planet, whose photosphere is located at deeper layers, although they may leave their imprints on the transmission spectrum, which can probe higher atmospheric layers. The cooler the planet, however, the deeper the photochemical “layers” extend, and the higher the likelihood that the photochemical products affect the infrared emission spectra. On our own solar-system giant planets, for example, methane photochemical products such as C_2_H_2_ and C_2_H_6_ survive throughout the stratosphere, dominating the mid-IR spectra and enabling more effective cooling of the stratospheres of these planets. In the case of HCN, its extended vertical distribution and relatively high abundance in cool to moderately warm gas giant planets makes it a very likely candidate for detection (e.g., Moses et al. 2011).

One-dimensional models indicate that in still upper layers, molecules are completely photodestroyed and atoms become the major constituents. In the absence of a hydrodynamic escape flow, molecular diffusion at a high-altitude homopause layer (typically in the 10^−6^ to 10^−9^ bar range for hot Jupiters) will cause the atmospheric constituents to become layered according to their weight, following their own density scale heights. Because of both this molecular diffusion and the strong X-ray/ultraviolet irradiation from the host star (which strongly heats the high-altitude thermosphere), atomic hydrogen becomes the only remaining dominant neutral constituent at high altitudes on close-in giant planets (e.g., Moses et al. 2011). If the upper atmosphere were to remain cool, the molecular region could extend to very high altitudes (e.g., Agúndez et al. 2014a). However, thermospheric photochemical models that include X-ray and extreme ultraviolet photolysis and photodissociative ionization of H_2_, ion chemistry, and realistic atmospheric escape processes (García Muñoz 2007; Koskinen et al. 2013) demonstrate that hydrodynamic winds fueled by the escaping gas can drag heavy atoms and ions to regions as high as the exosphere in strongly irradiated planets, but that molecules (including H_2_) cannot survive the large thermospheric temperatures on such planets. In fact, various types of atoms, from the lightest hydrogen to heavy metals, have been detected in the vertically extended, escaping atmospheres of hot Jupiters (Vidal-Madjar et al. 2003, 2004, 2013; Lecavelier Des Etangs et al. 2010; Fossati et al. 2010; Linsky et al. 2010).

Ionization of atmospheric constituents by galactic cosmic rays provides an additional source of disequilibrium chemistry on exoplanets. Rimmer and Helling (2013) and Rimmer et al. (2014) have explored the effects of cosmic rays on the atmospheric compositions of extrasolar giants planets. Charging of mineral grains, and potential subsequent electrical discharges, are another possible source of disequilibrium chemistry on extrasolar giant planets (see Helling et al. 2013; Bailey et al. 2014; Stark et al. 2014).

### 4.4 Sensitivity to Bulk Elemental Composition

Gas giant planets are expected to have nearly solar elemental compositions. However some may show a different metallicity, i.e., the abundances of elements heavier than helium scaled up or down by a similar factor, and/or elemental abundance ratios different from those found in the Sun. These deviations from the solar elemental composition, whatever their origin (see Sect. 6), can have a significant impact on the atmospheric composition.

In general, an enhancement of metallicity in H/He-dominated atmospheres of gas giant planets favors an increase in the abundances of molecules that contain multiple heavy atoms, i.e., CO and N_2_ are favored over CH_4_ and NH_3_. Molecules with more than two heavy atoms are even more favored, as occurs in the case of CO_2_, whose abundance increases as the square of metallicity (Zahnle et al. 2009a,b). See more details on this subject in Sects. 5.1 and 5.2.

In warm H/He-dominated atmospheres, one of the most critical elemental ratios is C/O. The issue is well known in the study of evolved stars, some of which can bring out to the surface enough carbon to revert the C/O abundance ratio, which in the Sun is 0.55 (Asplund et al. 2009). The high stability of CO under these conditions makes it lock almost all the limiting reactant, either C or O, allowing for the reactant in excess to form O-bearing molecules such as H_2_O when C/O < 1 and C-bearing molecules such as HCN and C_2_H_2_ when C/O > 1 (Tsuji 1973). The observational claim of a carbon-rich atmosphere in the hot Jupiter WASP-12b (Madhusudhan et al. 2011b), although still subjected to debate (Cowan et al. 2012a; Crossfield et al. 2012; Madhusudhan 2012; Swain et al. 2013; Mandell et al. 2013; Stevenson et al. 2014a,b; Kreidberg et al. 2015), has opened a window on the possible existence of carbon-rich giant extrasolar planets. Whatever their origin (see Sect. 6), such planets would show a chemical composition dramatically different from the more traditional gas giants with a nearly solar C/O ratio (i.e., oxygen-rich). In moderately warm and hot carbon-rich atmospheres, water is no longer an abundant constituent, the C-bearing molecules C_2_H_2_, CH_4_, and HCN become major constituents, and CO_2_ vanishes to a negligible level (Madhusudhan 2012; Kopparapu et al. 2012; Moses et al. 2013a,b; Venot et al. 2015; Heng and Lyons 2016). If the atmosphere is cool enough (<1000 K) then CH_4_ and H_2_O become the main reservoirs of carbon and oxygen, respectively, at the expense of other carbon- and oxygen-bearing molecules (Madhusudhan 2012; Moses et al. 2013a,b; Venot et al. 2015).

### 4.5 Clouds and Hazes

The terms *cloud* and *haze* are often used interchangeably, but here we follow the spirit of Marley et al. (2013) and use these terms to refer to two fundamentally different types of condensates that may appear in the atmospheres of gas giant planets. By *cloud* we refer to the typical “cooling clouds” (e.g., Rossow 1978) that form when a volume of gas is cooled dynamically or radiatively, such that the partial pressure of a constituent or constituents exceeds its saturation vapor pressure and condensation ensues. The typical equilibrium condensates expected along the cloud-condensation sequence in a hydrogen-dominated atmosphere (e.g., Lodders 2010; Marley et al. 2013; Morley et al. 2013) are thus considered clouds, regardless of their optical depth, horizontal extent, etc. We restrict the term “hazes” to refer to aerosols that form in situ by the action of photochemistry or any other disequilibrium chemical process (e.g., the so-called “soots” discussed by Zahnle et al. 2009a) or from photochemically produced gases flowing dynamically into cooler regions, where they can condense.

In the rainout scenario of cloud formation, a condensate forms and gravitationally settles in the atmospheric layer where the temperature equals its condensation temperature, and the atmosphere above the cloud becomes depleted in the elements that take part in that condensate (Lewis 1969; Fegley and Lodders 1994, 1996; Burrows and Sharp 1999; Lodders 1999; Lodders and Fegley 2002; Sudarsky et al. 2003). Thus, as one moves from the deep and hot atmosphere to upper and cooler regions, elements are progressively removed from the gas phase according to their refractory character. Chemical equilibrium calculations including gas and condensed species are very useful to identify the most plausible cloud-forming species (see Fig. 2). In the rainout approach, chemical equilibrium is solved in order of decreasing temperature and, when a given species is found to condense the elements which take part in that condensate are removed (in the corresponding stoichiometric proportions) before continuing to solve chemical equilibrium at lower temperatures. Thus, it is not only necessary to know the possible condensates but also the condensation sequence and the main condensates that deplete each element.

Unlike the case of Jupiter, whose atmosphere is so cold that water and ammonia condense to form tropospheric clouds, most extrasolar giant planets characterized to date are sufficiently hot to ensure the survival of water vapor and other volatiles in their atmospheres. In hot Jupiters only the most refractory species can condense to form clouds. Although the exact temperature-pressure profile of the planet in question controls which refractory species will condense first (i.e., at the deepest pressures; see Lodders 2010), aluminum, titanium, and calcium are expected to be the first elements to be removed from the gas phase, condensing as corundum (Al_2_O_3_), Ca-aluminates such as hibonite (CaAl_12_O_19_), and Catitanates such as perovskite (CaTiO_3_). Therefore, atmospheres hotter than about 2000 K can maintain titanium in the gas phase to form oxides such as TiO, while cooler atmospheres would deplete most of this element in the form of perovskite or other Ca-titanate (Burrows and Sharp 1999; Lodders 2002). In fact, it has been proposed that in the atmospheres of very hot giant exoplanets, the survival in the gas phase of species such as TiO and VO can provide a sufficiently high opacity at optical wavelengths as to induce a thermal inversion (Hubeny et al. 2003; Fortney et al. 2008a; Spiegel et al. 2009). The detections of TiO and VO in brown dwarf atmospheres at high temperatures (Kirkpatrick 2005) provide support to this proposition. However, TiO and VO have not yet been unambiguously identified in the atmospheres of strongly irradiated hot Jupiters (Désert et al. 2008; Huitson et al. 2013; Mancini et al. 2013; Swain et al. 2013; Sing et al. 2013; Gibson et al. 2013; Stevenson et al. 2014b; Schlawin et al. 2014; Hoeijmakers et al. 2015), though recently a potential detection of TiO has been reported for one of the most irradiated hot Jupiters WASP-33b (Haynes et al. 2015).

At temperatures below ~1800 K, iron is expected to condense homogeneously, and magnesium, together with a good fraction of silicon, are expected to deplete in the form of silicates such as Mg_2_SiO_4_ and MgSiO_3_. The presence of such type of clouds has been inferred from transit observations of the hot Jupiter HD 189733b (Pont et al. 2008, 2013; Lecavelier Des Etangs et al. 2008a; Sing et al. 2009; Gibson et al. 2012). The alkali metals Na and K can be depleted in the form of the aluminosilicates NaAlSi_3_O_8_ and KAlSi_3_O_8_ at temperatures around 1500 K (Burrows and Sharp 1999), although in the rainout scenario it is likely that at these temperatures most of the aluminum is no longer available in the gas phase, in which case Na and K would condense at ~1000 K as Na_2_S and KCl, respectively (Lodders 1999). In sufficiently hot atmospheres neutral Na and K atoms can survive in the gas phase and can be readily detected through the Na I doublet at 589.0 nm and 589.6 nm and the K I doublet at 766.5 nm and 769.9 nm (Charbonneau et al. 2002; Redfield et al. 2008; Wood et al. 2011; Sing et al. 2011a, 2012; Nikolov et al. 2014; Murgas et al. 2014).

The rainout scenario provides a useful methodology to predict the composition of the clouds that may be present in a given atmosphere and the base level at which each cloud forms. However, the computation of the size and concentration of particles above this level and the horizontal distribution of the clouds, i.e., how particles nucleate and grow and are affected by atmospheric dynamics, still remains a formidable challenge. Nevertheless, efforts to this end have been undertaken by various groups (Ackerman and Marley 2001; Helling et al. 2008; Freytag et al. 2010; Parmentier et al. 2013; Morley et al. 2013; Lee et al. 2015).

The photochemical formation of organic hazes in the atmospheres of hot Jupiters has been addressed in a couple of theoretical studies (Liang et al. 2004; Zahnle et al. 2009a). The general view provided by these studies is that in hot atmospheres, most of the carbon remains locked into CO, while in cooler atmospheres the larger amount of carbon stored into CH_4_ can be photochemically driven to larger hydrocarbons, eventually producing hydrocarbon aerosols or soots. Cooler planets would therefore be expected to have more prevalent photochemical hazes. This scenario agrees with the presence of hazes in the cold atmospheres of Jupiter, Saturn, and Titan (e.g., Tomasko et al. 2005; Zhang et al. 2013), although it is in contradiction with the inference of aerosols in the very hot atmosphere of WASP-12b (Sing et al. 2013), unless these aerosols result from the most refractory species along the cloud-condensation sequence (e.g., Al_2_O_3_) at the cooler limb of the planet (Sing et al. 2013), or unless the planet is indeed carbon-rich (and thus contains the more photochemically active molecules HCN and C_2_H_2_, e.g., Kopparapu et al. 2012 and Moses et al. 2013b). It is clear that more work is needed in both the theoretical and observational sides to better understand the formation and role of hazes in extrasolar giant planets.

### 4.6 Hot Jupiters Versus Directly Imaged Planets

One of the most recent and formidable successes achieved in the field of exoplanets has been the detection by direct imaging of young and self-luminous gas giant planets, opening the way to characterize their atmospheres by direct spectroscopy (Janson et al. 2010, 2013; Hinz et al. 2010; Bowler et al. 2010; Currie et al. 2011; Barman et al. 2011; Skemer et al. 2012; Oppenheimer et al. 2013; Konopacky et al. 2013; Chilcote et al. 2015). The few planets that have been characterized by this method are more massive than Jupiter and have effective temperatures in the range 600–1700 K. Thus, they have some similarities with brown dwarfs and free-floating planets (Zapatero Osorio et al. 2000; Burrows et al. 2001, 2003). Directly imaged planets share also some characteristics with hot Jupiters, as both are gas giants and hot. However, unlike hot Jupiters, directly imaged planets orbit far from their host star and are young, so that they are heated predominantly from the interior rather than irradiated by the star. This difference leads to qualitative differences in the dynamical and thermal structure of the atmosphere. In hot Jupiters, the high irradiation causes the atmospheres to be radiative in almost the entire observable atmosphere, and the temperature profile is isothermal in the lower atmosphere, at pressures above ~1 bar (see e.g. Burrows et al. 2008), before convection dominates in the deep atmosphere (≳100 bar). On the other hand, the atmospheres of self-luminous planets and brown dwarfs are largely driven by convection, leading to adiabatic temperature gradients even in the observable atmospheres.

The atmospheres of hot Jupiters and directly imaged planets have temperatures of the same order and are expected to have a nearly solar elemental composition, so that one would expect a similar atmospheric chemistry in both types of planets. There are, however, a couple of major differences. First, the atmospheres of directly imaged planets are likely less affected by photochemistry than in the case of hot Jupiters because of their much larger orbital distances; however, directly imaged planets tend to orbit young stars, and young stars tend to have high ultraviolet output, so photochemistry will not be negligible on these planets. And second, the source of heat being located in the interior of the planet rather than outside imprints differences in the atmospheric thermal profile. According to the recent theoretical study by Zahnle and Marley (2014), the higher temperatures in the deep atmosphere of self-luminous planets favor CO over CH_4_ in deep layers, but also in the upper observable atmosphere as a consequence of upward mixing. Methane is therefore predicted to be a minor atmospheric constituent in most self-luminous planets characterized to date, except for a couple of planets with cool atmospheres whose near-infrared spectra show evidence for CH_4_ absorption, such as GJ 504b (Janson et al. 2013), 51 Eri b (Macintosh et al. 2015), and HR 8799b (Barman et al. 2015). Years of study of brown dwarf atmospheres (see e.g., Marley and Robinson 2015), which share many similarities with the atmospheres of self-luminous planets, provide an invaluable basis to aid in the understanding of atmospheric chemistry of self-luminous planets, although further observations are still needed.

## 5 Theory of Atmospheric Chemistry of Exo-Neptunes and Super-Earths

The same chemical processes discussed above for giant planets—thermochemical equilibrium, disequilibrium quenching due to transport, and disequilibrium photochemistry—also affect smaller planets, but the basic ingredients available to the atmospheres of smaller planets differ from those of gas giants. These differences emerge early on in the planet’s evolution, with such factors as the dust/planetesimal surface density distribution within the protoplanetary disk (see chapters on protoplanetary disk evolution in Oberg et al. 2016, this issue; Fang et al. 2016, this issue), the planet’s initial formation and feeding-zone location within the disk, and the planet’s migration history being important parameters that control how massive the planet becomes, what atmospheric volatiles are collected, and how much atmospheric hydrogen and helium are retained (e.g., Pollack et al. 1996; Lissauer and Stevenson 2007; D’Angelo et al. 2010; Dodson-Robinson and Bodenheimer 2010; Marboeuf et al. 2014; Ali-Dib et al. 2014; Madhusudhan et al. 2014a; Helling et al. 2014). In the gravitational-instability theory for giant-planet formation (e.g., Boss 1997), giant planets are expected to end up with a metallicity similar to the host star. In the core-accretion theory of giant-planet formation (e.g. Pollack et al. 1996), protoplanetary cores form from the accretion of solid planetesimals, and gas accretion rates are initially slow. As the solid protoplanetary core grows, it can accrete more and more of the surrounding hydrogen-rich nebular gas (Lammer et al. 2014; Stökl et al. 2015, also see chapter by Massol et al. 2016, this issue). When the core becomes massive enough, with a critical mass of order ~10 Earth masses (but cf. Venturini et al. 2015), the protoplanet can experience a runaway gas-accretion phase in which the nebular gas is rapidly accreted onto the protoplanetary core. Planets that reach this runaway gas-accretion stage become hydrogen- and helium-rich gas-giant planets. Planets that don’t reach this stage contain less H and He.

Whether a protoplanet can reach this runaway gas-accretion stage or not depends largely on the accretion rate of solids in comparison to the time scale for dissipation of gas from the disk. Just beyond condensation fronts in the disk, such as the water-ice “snow line”, the surface density of solid material becomes large, leading to more rapid accretion of solids and a reduced time scale for formation of massive protoplanetary cores (e.g., Stevenson and Lunine 1988; Ciesla and Cuzzi 2006; Dodson-Robinson et al. 2009). The formation of giant planets is thus expected to be particularly efficient near the disk snow line. In lower-surface-density regions of the disk farther out from the snow line, the solid accretion rate is slower, and a protoplanet may never reach this runaway gas-accretion phase, leading to less accumulation of the nebular gas. In this classical picture, the formation of Uranus and Neptune occurred too slowly to allow these planets to fully reach the runaway gas-accretion phase, leading to “ice giant” planets whose total mass is dominated by heavier volatiles such H_2_O rather than hydrogen and helium (e.g., Lissauer and Stevenson 2007; Fortney and Nettelmann 2010). The overall initial mass fraction of H/He in a planet’s atmosphere is a sensitive function of the disk characteristics and lifetime, with small changes in disk properties or evolutionary history having a significant impact on the resulting mass and composition of planets that are intermediate in size between gas giants and solid terrestrial planets (e.g., Helled and Bodenheimer 2014; Lammer et al. 2014; Luger et al. 2015). While generally atmospheres of ice-giants are expected to be H_2_-rich, recent theoretical studies have also suggested the possibility of He-dominated atmospheres (Hu et al. 2015b).

In this section, we discuss the atmospheric chemistry of these intermediate-sized planets, the so-called super-Earths, mini-Neptunes, and Neptune-sized planets, with radii spanning ~1−6 *R*_⊕_, that are observed to constitute a large percentage of the known planets in our galaxy (e.g., Borucki et al. 2011; Mayor et al. 2011; Howard et al. 2012; Batalha et al. 2013; Fressin et al. 2013; Burke et al. 2014; Rowe et al. 2015). These planets will typically contain less hydrogen and helium (and thus a higher atmospheric metallicity) than giant planets because of the less efficient accretion of nebular gas during their formation and evolution, as well as the higher likelihood of the escape of light gases over time. Outgassing from the planetary interior is expected to contribute additional volatiles—a component of the atmosphere that will become increasingly important for smaller planets—and other evolutionary processes such as atmospheric escape, impact delivery or erosion, atmosphere-surface exchange, weathering, and sequestration of volatiles into the interior can have and major influence on atmospheric composition and chemistry. The stochastic nature of the different possible evolutionary pathways is expected to lead to highly diverse atmospheric properties for intermediate and small planets (e.g. Pepin 2006; Dodson-Robinson and Bodenheimer 2010; Rogers et al. 2011; Lopez and Fortney 2013; Fortney et al. 2013; Haghighipour 2013; Leconte et al. 2014). Here, we consider the atmospheric chemistry of exo-Neptunes and super-Earths with widely diverse volatile contents, as well as the chemistry of outgassed atmospheres of smaller, hot, rocky planets. The atmospheric chemistry of terrestrial planets near the habitable zone is briefly discussed in Sect. 8.

### 5.1 Chemical Equilibrium in Exo-Neptune and Super-Earth Atmospheres

Thermochemical equilibrium can be maintained in high-temperature, high-pressure regions of exoplanet atmospheres (see Sect. 4.1), so equilibrium conditions are appropriate to consider to first order for any super-Earths or exo-Neptunes with thick, hot atmospheres. This statement is true whether the planet is a low-density fluid “ice giant”, like Neptune itself, or has a thick atmosphere overlying a solid surface, like Venus. Even secondary atmospheres produced from the outgassing of interior volatiles during and after the accretion phase can lead to thick, high-pressure atmospheres that remain hot at depth due to accretional energy, radioactive decay in the interior, tidal heating, strong stellar irradiation, and/or a greenhouse effect.

Our own terrestrial planets have demonstrated the importance of secondary outgassing of interior volatiles in shaping the atmospheric properties of solid-surface planets (Pepin 2006). The theoretical equilibrium composition of super-Earth atmospheres dominated by such an outgassing source has been explored by several investigators. For example, Elkins-Tanton and Seager (2008) have examined the wide range of atmospheric masses and compositions that can result from the degassing of different meteoritic compositions during the planet’s accretion phase. Schaefer and Fegley (2010) have performed similar more detailed calculations, albeit with a focus on the early Earth, to investigate the chemistry of potential steam atmospheres, such as those predicted to be associated with magma oceans (e.g., Abe and Matsui 1985) thought to form during the accretion of the Earth and other terrestrial (exo-)planets. Both Schaefer and Fegley (2010) and Elkins-Tanton and Seager (2008) find that the resulting atmospheric composition is a sensitive function of the assumed composition of the meteoritic material being accreted—water-dominated steam atmospheres occur only for CI and CM chondritic starting material. For other assumed meteoritic starting compositions, the atmospheres can be dominated by CO_2_, N_2_, H_2_, CH_4_, or CO, depending on the starting material composition and atmospheric temperatures (Schaefer and Fegley 2010). Schaefer and Fegley (2011) have also pursued the potential atmosphere-surface buffering of hot atmospheres in equilibrium with planetary surfaces.

The possible formation of exotic (by solar-system standards) silicate atmospheres on hot super-Earths, in which volatile elements such as H, C, N, S, and Cl have already escaped from the planet, is explored by Schaefer and Fegley (2009) and Miguel et al. (2011). In these calculations, the atmosphere is assumed to be in gas-melt equilibrium with a volatile-free magma ocean or partially molten lithosphere. Their results indicate that such atmospheres can be composed largely of atomic Na, O_2_, O, with SiO, Fe, and/or Mg, depending on the planet’s orbital distance and resulting atmospheric temperature. Ito et al. (2015) have performed a similar set of equilibrium calculations for super-Earth atmospheric compositions over a volatile-free magma ocean of various assumed compositions; they discuss the resulting thermal structure, spectroscopy, and detectability of such atmospheres. They find that SiO, in particular, affects the atmospheric opacity, causing thermal inversions and notable infrared emission signatures.

**Fig. 5:**
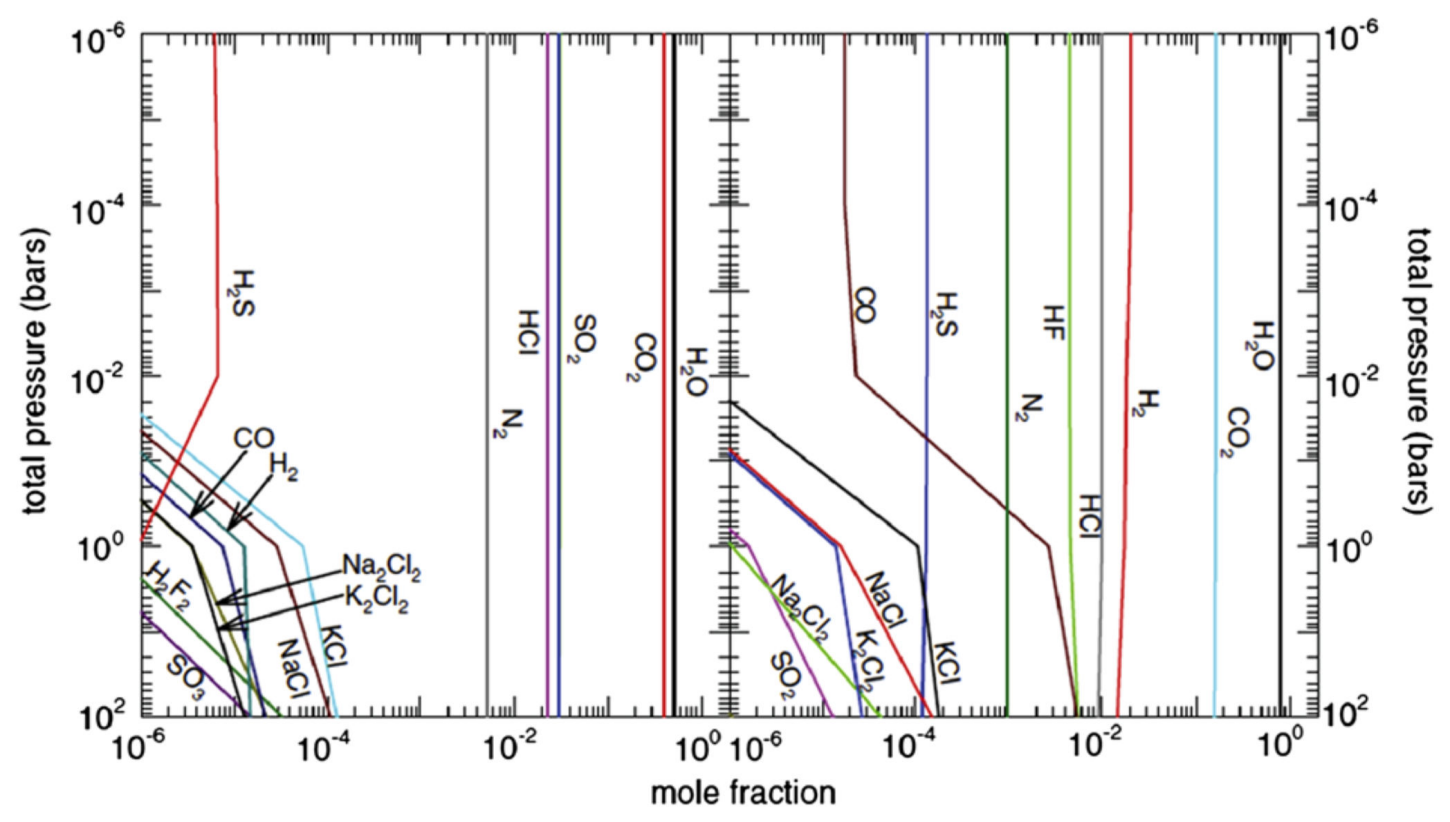
Equilibrium atmospheric composition for a GJ 1214b-like exoplanet, assuming the atmosphere results from outgassing from a high-temperature felsic silicate terrestrial crustal composition (*left*) or a more mafic bulk-Earth silicate composition (*right*). Figure from Schaefer et al. (2012)

Schaefer et al. (2012) have extended their earlier 2010 investigation to consider a wider range of parameter space that might be relevant to outgassed atmospheres of hot terrestrial exoplanets and super-Earths, including considerations of variations in the relative abundances of elements such as H, C, and O. Again, these authors emphasize that the equilibrium atmospheric composition of such secondary outgassed atmospheres depends strongly on the source material and temperatures. Outgassing from accreted material with the composition of the bulk silicate Earth or the terrestrial crust would result in a water-dominated atmosphere under conditions relative to GJ 1214b, with CO_2_ as an important secondary component (see Fig. 5), whereas O_2_ and Na could dominate in the hotter, lower-density atmosphere of CoRoT-7b (Schaefer et al. 2012; see also Léger et al. 2011). The chemistry of possible hot equilibrium atmospheres of Earth-like planets after giant impact events is explored by Lupu et al. (2014), who find that the main consituents are water and CO_2_, along with smaller amounts of HCl, H_2_, HF, CO, N_2_, alkali halides, SO_2_, and H_2_S.

The equilibrium atmospheric composition of warm super-Earths like GJ 581c is investigated by Miller-Ricci et al. (2009), for an assumed cometary-like complement of volatiles with variable hydrogen content. For hydrogen-rich atmospheres, the dominant constituents are H_2_, H_2_O, CH_4_, and NH_3_. For the hydrogen-poor situation, the resulting Venus-like atmosphere has dominant constituents CO_2_ and N_2_, with much less H_2_O. For the “intermediate” hydrogen case, the atmosphere has an interesting mixture of dominant constituents like H_2_O, H_2_, CO_2_, and CH_4_. The overall hydrogen content therefore has a strong influence on the resulting composition.

Observations of exoplanets for which both mass and radius have been determined show a transition in planetary bulk densities near radii of ~1.5−2 *R*_⊕_; the smallest planets tend to be dense and presumably rocky, with at best a tiny mass fraction of light volatiles like hydrogen and helium, and larger planets tend to require increasingly larger mass fractions of H/He envelopes to explain the bulk density (cf. Weiss et al. 2013; Weiss and Marcy 2014; Marcy et al. 2014; Wu and Lithwick 2013; Hadden and Lithwick 2014; Rogers 2015). Population-synthesis models and other theoretical arguments suggest that the trend of decreasing hydrogen and helium content with decreasing planetary size is a natural consequence of planetary formation and evolution (Miller and Fortney 2011; Rogers et al. 2011; Lopez et al. 2012; Fortney et al. 2013; Owen and Wu 2013; Lopez and Fortney 2013, 2014; Benz et al. 2014; Wolfgang and Lopez 2015). Regardless of whether close-in exo-Neptunes and super-Earths with comparatively large H/He contents formed farther out in the disk and migrated/scattered inward (e.g., Ida and Lin 2005; Alibert et al. 2006; Terquem and Papaloizou 2007; Kennedy and Kenyon 2008; Mordasini et al. 2012; Inamdar and Schlichting 2015) or whether they formed in situ (Hansen and Murray 2012, 2013; Chiang and Laughlin 2013; Chatterjee and Tan 2014), the amount of hydrogen in the atmospheric envelope has a strong influence on the resulting atmospheric composition. The metallicity (or bulk mole fraction of H, in general) of the atmosphere is therefore an important parameter controlling the chemistry of low-density super-Earths and exo-Neptunes.

**Fig. 6:**
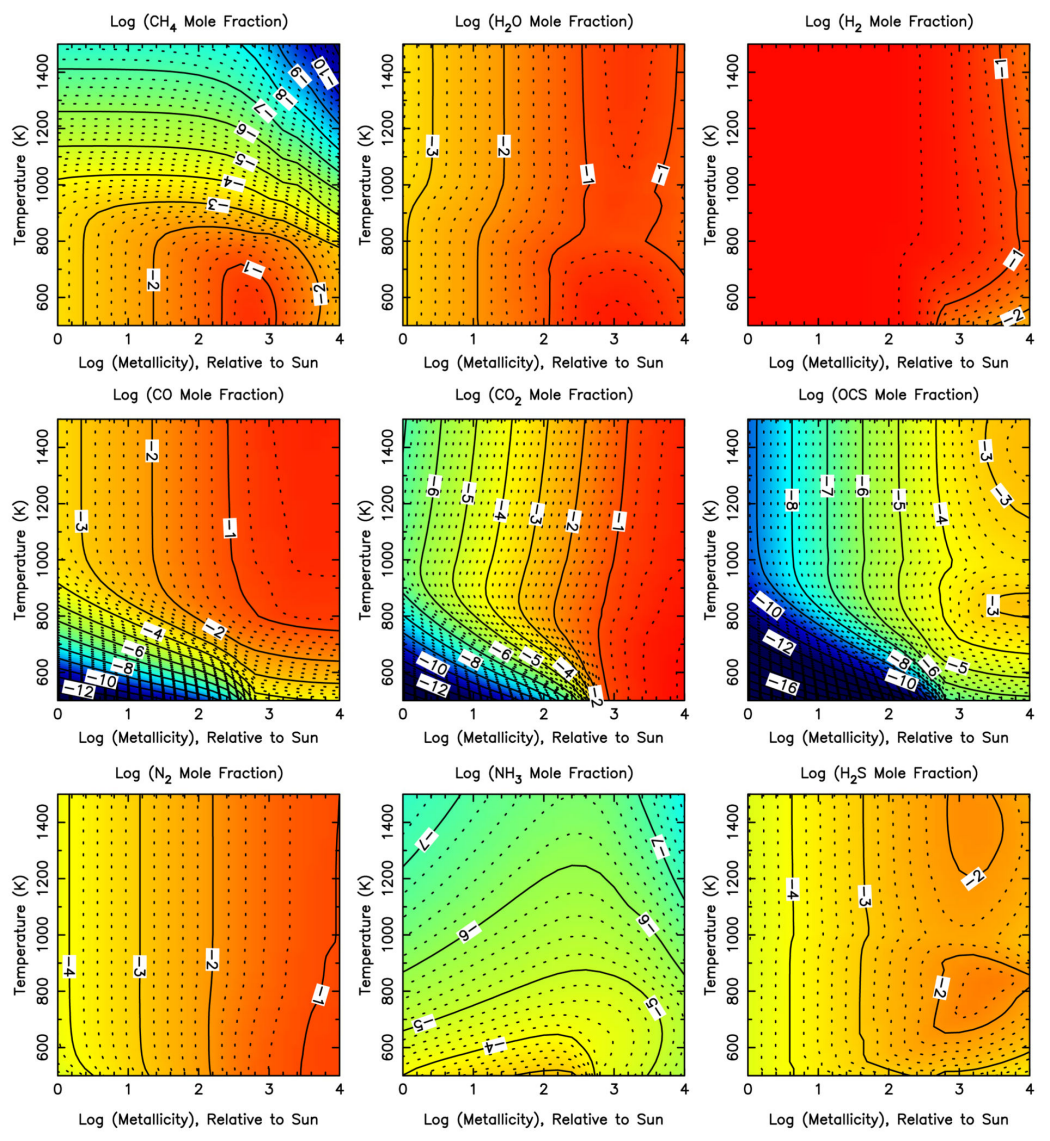
Equilibrium mixing ratios for various atmospheric constituents as a function of temperature and metallicity for an assumed typical photospheric pressure of 0.1 bar. Figure adapted from Moses et al. (2013a)

The effect of metallicity on the equilibrium composition, temperature structure, and/or spectra of exoplanet atmospheres has been studied by Lodders and Fegley (2002), Visscher et al. (2006, 2010a), and Fortney et al. (2008b) for metallicities up to 3–5× solar; Zahnle et al. (2009a,b), Spiegel et al. (2010), Fortney et al. (2010), Lewis et al. (2010), Line et al. (2011) for metallicities up to 30−50× solar; Agúndez et al. (2014b) and Venot et al. (2014) for metallicities up to 100× solar; and Moses et al. (2013a), Hu and Seager (2014), and Miguel et al. (2015) for very high metallicities (e.g., up to and beyond 1000× solar). These investigations reach concensus on several general trends. First, high-metallicity planets will have higher temperatures at lower pressures than otherwise similar low-metallicity planets, due to greater atmospheric opacity from the heavy (i.e., non-H_2_) molecular constituents; that is, the photosphere will shift to higher altitudes. Second, the CO/CH_4_ and N_2_/NH_3_ equal-abundance curves shift to lower temperatures with higher metallicities, leading to a higher likelihood that CO and N_2_ will be important carbon and nitrogen components, respectively, of a high-metallicity planetary atmosphere. Third, molecules with multiple heavy elements, such as CO, CO_2_, N_2_, CS, S_2_ become favored at the expense of molecules that just contain one heavy element, like CH_4_, NH_3_, H_2_S, as the metallicity increases—an effect that is particularly notable for molecules that contain more than two heavy elements, such as CO_2_ and OCS (see Fig. 6). At sufficiently high metallicities, CO_2_ will even replace H_2_ as the dominant atmospheric constituent for an otherwise solar-composition atmosphere. Molecular hydrogen remains a major constituent of the atmosphere under the conditions studied in Fig. 6, but it ceases to dominate at the highest metallicities considered (e.g., several thousand times solar). Water increases roughly linearly with metallicity until metallicities of ~1000 times solar, at which point the lower H mole fraction begins to adversely impact all H-bearing molecules. The expected depletion of hydrogen in super-Earths and exo-Neptunes due to inefficient accretion of the nebular gas and/or efficient escape of hydrogen at small orbital distances is therefore expected to lead to increased atmospheric mean molecular weights and a wide variety of interesting atmospheric compositions.

The sensitivity-to-metallicity calculations in Fig. 6 and in most of the investigations described in the previous paragraph have been performed assuming that the relative abundances of all elements other than H, He (and sometimes Ne) remain in solar proportion. Of course, that is not likely going to be true for exoplanetary atmospheres, given the different formation scenarios and evolutionary process at work. The sensitivity of the chemical equilibrium composition to changes in the C/O ratio at near-solar metallicities is discussed in Sect. 4.4. Moses et al. (2013a) examine the more general case of the sensitivity of the equilibrium atmospheric composition to both the C/O ratio and metallicity (i.e., to the relative abundances of H, C, and O) as a function of temperature, and Hu and Seager (2014) perform a similar general analysis considering disequilibrium processes like photochemistry (see also Sect. 5.2). Here is where the diversity of potential heavy-element-rich super-Earths and exo-Neptunes really stands out. The dominant equilibrium atmospheric constituent on intermediate-sized planets will typically be H_2_ at low-enough metallicities (e.g., less than several hundred times solar) but can become H_2_O at moderately high metallicities and subsolar C/O ratios, can become CO_2_ at solar-like and subsolar C/O ratios and high metallicities, can potentially become CO at high metallicities and C/O ratios near unity (depending on graphite stability), and can even become O_2_ at very low C/O ratios and high metallicities. At high C/O ratios and low metallicities, CH_4_ is an important atmospheric component at low temperatures, while HCN and C_2_H_2_ become more important carbon phases at high temperatures. Hu and Seager (2014) suggest that hydrocarbons like C_2_H_2_ and C_2_H_4_ can even become dominant atmospheric constituents at bulk C/O ratios greater than ~2, but they have ignored graphite formation in their calculations. At high C/O ratios, the graphite stability field for equilibrium conditions is greatly expanded (see Moses et al. 2013a), and for a large range of temperature and metallicity conditions, graphite will condense out and sequester a significant fraction of the carbon when bulk C/O ratios are greater than ~ 1 (or even C/O <1 for high metallicities), leaving the remaining gas much less carbon-rich. Therefore, high-metallicity atmospheres can achieve an unusual state where the dominant gas is CO_2_, even for bulk C/O ratios greatly exceeding unity (Moses et al. 2013a). It is also possible that atmospheric O may be lost preferentially by photodissociation of H_2_O and subsequent hydrodynamic escape of O, thereby changing the C/O ratio (Chassefière 1996; Luger and Barnes 2015).

Between the potential for hot, silicate- and metal-rich outgassed atmospheres and the variety of volatile compositions available from inefficient accretion of nebular gas, the super-Earths and exo-Neptune population can be expected to have a rich diversity of atmospheric compositions from thermochemical equilibrium considerations, and disequilibrium processes (below) simply augment this possible diversity.

### 5.2 Disequilibrium Chemistry in Exo-Neptune and Super-Earth Atmospheres

Both photochemistry and transport-induced quenching can affect the atmospheric composition of intermediate-size planets, just as on giant planets (see Sects. 4.2 and 4.3). The first photochemical models specifically applied to intermediate-sized planets were those of Line et al. (2011) for the exo-Neptune GJ 436b and Miller-Ricci Kempton et al. (2012) for the super-Earth GJ 1214b, although the generic, higher-metallicity, hot-Jupiter models studied by Zahnle et al. (2009a,b) should also have relevance to some exoplanets in the intermediate-size range. Thermochemistry, photochemistry, and transport-induced quenching are considered in the Miller-Ricci Kempton et al. (2012) and Line et al. (2011) models, and the planets are assumed to have H_2_-rich atmospheres with metallicities up to 30−50× solar. Given that both GJ 1214b and GJ 436b are expected to be relatively cool transiting planets, the results from both models are qualitatively similar. Methane is expected to be the dominant equilibrium carbon species for both planets up to 30−50× solar metallicity for the other conditions considered. At the quench point where interconversion between CH_4_ and CO shuts down, methane is the main carbon component. However, transport-induced quenching causes CO to be more abundant than it otherwise would have been in equilibrium. This situation represents the opposite of the case for hotter planets, where quenching in the CO-dominated regime causes CO to be the major carbon component, with methane then being a less-abundant, but still important, quenched component (see Sect. 4.2). Photolysis of methane at high altitudes leads to the production of C_2_H*_x_* hydrocarbons and, because of interactions with water photolysis products, the photochemical production of CO and CO_2_. Coupled methane-ammonia photochemistry causes the production of HCN. However, methane is not removed from the photospheric region of either planet due to photochemistry (as was suggested as a possibility by Madhusudhan and Seager 2011), which is problematic, given that cloudless H_2_-rich models with equilibrium methane abundances do not reproduce transit and eclipse observations of these planets (e.g., Stevenson et al. 2010; Désert et al. 2011; Bean et al. 2011; Berta et al. 2012; Kreidberg et al. 2014a).

**Fig. 7:**
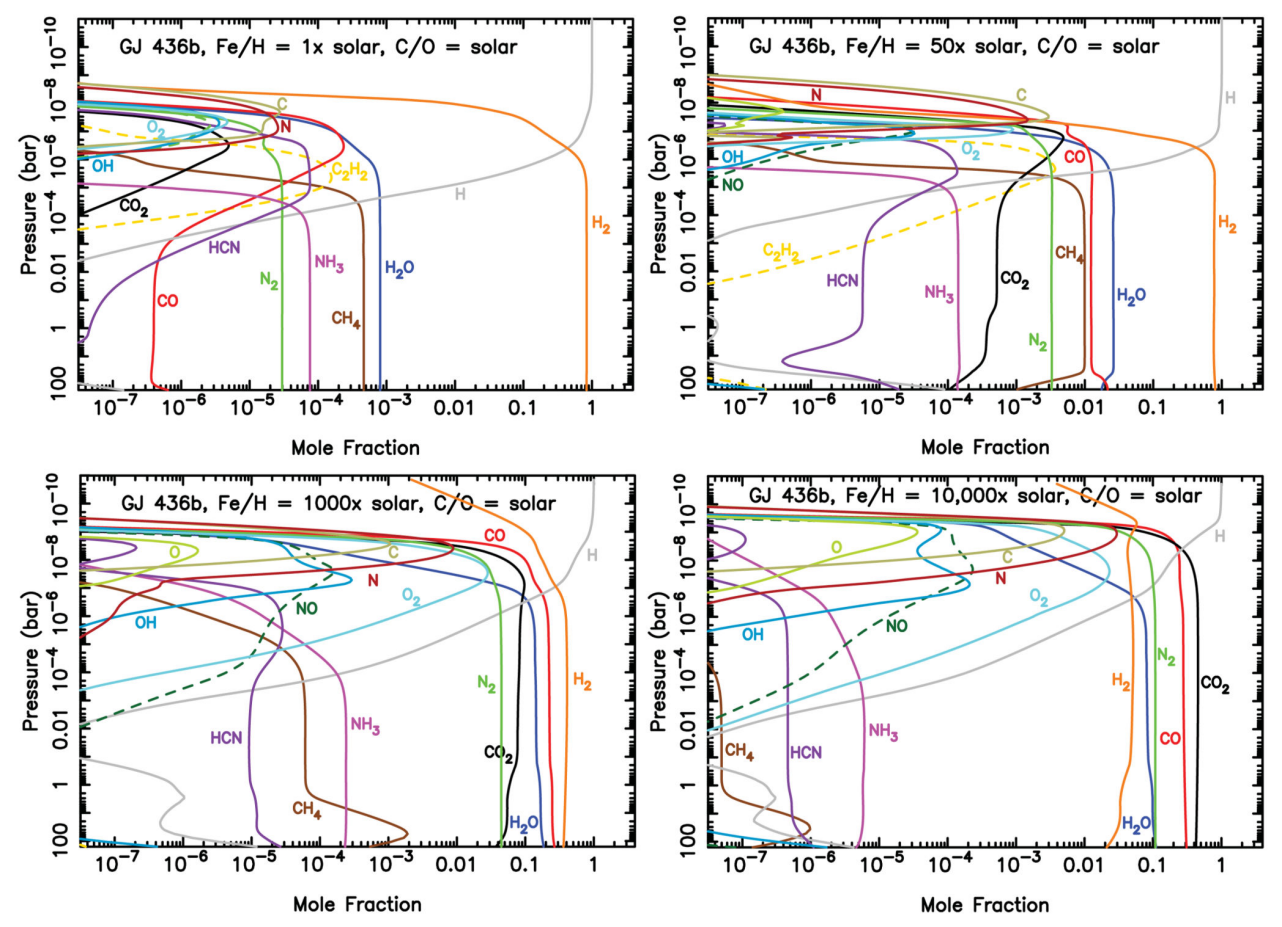
Vertical mixing-ratio profiles for several major atmospheric constituents in disequilibrium thermo/photochemical models of GJ 436b, for assumed metallicities of 1× solar (*top left*), 50× solar (*top right*), 1000× solar (*bottom left*), and 10000× solar (*bottom right*). Note the major change in the abundance of CO_2_, CO, and CH_4_ with the increase in metallicity (figure from Moses et al. 2013a)

This model-data mismatch led Moses et al. (2013a) to suggest that intermediate-sized planets could have much higher metallicities than previously considered, as long as their overall H content remains consistent with constraints supplied by the planet’s bulk density. As discussed above, higher metallicities lead to hotter photospheres at lower pressures and shift the atmosphere toward the CO and CO_2_ stability fields and away from the CH_4_ stability field. Figure 7 shows how the atmospheric composition and photochemistry change for GJ 436b as the metallicity is assumed to increase from 1× solar to 10000× solar. The atmosphere transitions from being hydrogen-dominated, with abundant hydrogen-saturated components like H_2_O, CH_4_, and NH_3_ and photochemically produced hydrocarbons and nitriles at low metallicities, to becoming CO_2_-, CO-, H_2_O-, and N_2_-rich, with more oxidized photochemical products like O_2_ and NO at high metallicities. Condensed graphite is a likely cloud component in the very-high-metallicity scenarios. Moses et al. (2013a) find that GJ 436b models with metallicities in the 230–2000× solar range provide the best overall consistency with both the planet’s inferred interior structure (see Nettelmann et al. 2010, and references therein) and its apparent CO-rich, CH_4_-poor dayside atmosphere and relatively flat transmission spectrum (e.g., Stevenson et al. 2010; Lanotte et al. 2014; Knutson et al. 2014a).

Venot et al. (2013) also consider the thermo/photochemistry of a presumed 100× solar metallicity atmosphere for GJ 436b, focusing in particular on how their new measurements of the CO_2_ ultraviolet absorption cross sections at high temperatures (up to 800 K) affect the model abundances, and how stellar type affects the results. Venot et al. (2013) find that their results for the predicted abundances of NH_3_, CO_2_, and CO are especially affected by the temperature-sensitive CO_2_ cross sections, with models that are irradiated by hotter stars (with their corresponding higher near-ultraviolet fluxes) exhibiting the biggest change in abundance due to use of high-temperature CO_2_ cross sections.

Thermo/photochemical models for GJ 436b are also presented by Agúndez et al. (2014b) and Miguel et al. (2015). Agúndez et al. (2014b) explore the influence of tidal heating and metallicity on the thermal structure and resulting disequilibrium chemistry of planet, calculating the thermal structure self-consistently. Both greater tidal heating at depth and higher metallicities help shift the thermal structure into the CO dominated regime, again helping favor higher CO/CH_4_ ratios and a greater CO_2_ abundance. Miguel et al. (2015) examine the sensitivity of the atmospheric composition of GJ 436b to the flux in the stellar Lyman alpha line for both low-metallicity and high-metallicity models. They find that constituent abundances in the upper stratosphere at pressures less than ~0.1 mbar are affected by Lyman alpha, with stronger fluxes favoring the destruction of molecules and the resulting dominance of atomic species. Note, however, that when one considers the formation and presence of an extended hot thermosphere (García Muñoz 2007; Koskinen et al. 2013), which is the likely consequence of strong X-ray and EUV radiation received by close-in extrasolar planets— including GJ 436b (see Sanz-Forcada et al. 2011; Koskinen et al. 2014)—much or all of the Lyman alpha flux itself could be absorbed within the thermosphere (Lavvas et al. 2011), never reaching the stratosphere. On the other hand, other strong X-ray and EUV lines could have a similar effect to that described in Miguel et al. (2015), ultimately moving the base of the atomic-dominated hot thermosphere to deeper pressures for stronger EUV and X-ray fluxes.

**Fig. 8:**
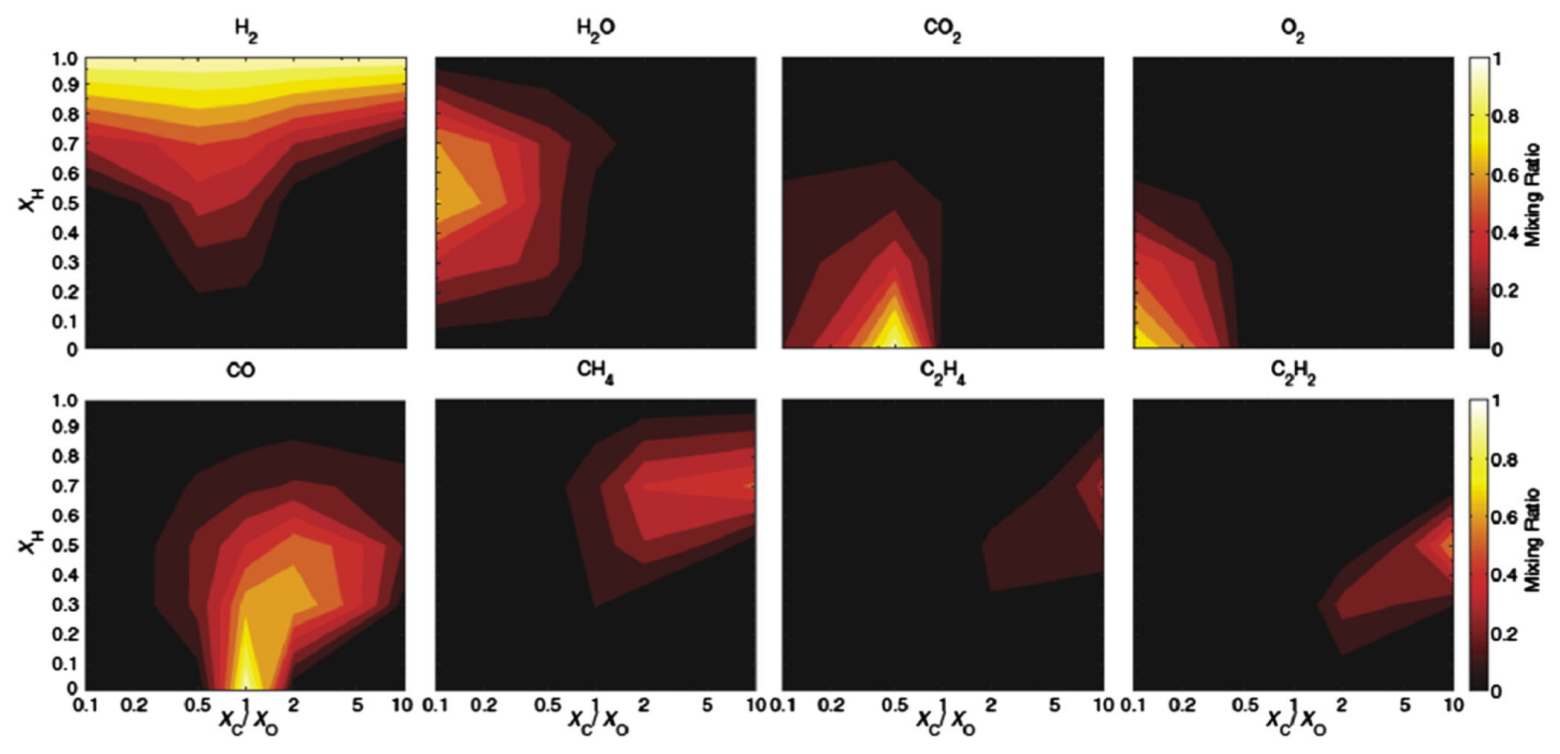
Mixing ratios for important atmospheric constituents vertically averaged over the pressure range 1–100 mbar from a disequilibrium thermo/photochemistry model of the super-Earth GJ 1214b, as a function of the bulk atmospheric H mole fraction (*X_H_* , where smaller values correspond to higher metallicities) and the C/O ratio (*X_C_/X_O_*). Figure from Hu and Seager (2014)

Hu and Seager (2014) have examined the sensitivity of the disequilibrium atmospheric composition of several intermediate-sized exoplanets (e.g., GJ 1214b, HD 97658b, and 55 Cnc e) to the general variation of H, O, and C elemental abundances, using a thermo/photochemistry model that self-consistently calculates temperatures. Figure 8 shows their results for GJ 1214b. These results are similar to the equilibrium results shown in Fig. 6 in that the atmospheric composition can be highly diverse, depending on the relative abundances of the key volatile elements H, C, and O. At low metallicities, H_2_ dominates, but GJ 1214b’s atmosphere could also be dominated by H_2_O, CO_2_, CO, or O_2_, depending on the overall H mole fraction and C/O ratio. Hu and Seager (2014) suggest that the atmosphere could be dominated by hydrocarbons such as C_2_H_2_ and C_2_H_4_ for high C/O ratios and moderately high metallicities; however, graphite condensation has been neglected in their calculations. Unless graphite formation is somehow kinetically inhibited (see Moses et al. 2013a), condensation of graphite will tie up much of the excess carbon, causing the remaining vapor to have lower C/O ratios, disfavoring hydrocarbons as the dominant constituents. Hu and Seager (2014) also show how the results for intermediate-sized planets can change with stellar flux (and thus temperature), and they suggest classification schemes for super-Earths with thick atmospheres based on the dominant constituents that appear for different H mole fractions and C/O ratios. In an earlier study, Hu et al. (2013) examine the fate of sulfur species from photochemistry in atmospheres of different dominant compositions relevant to super-Earths.

Venot et al. (2014) have investigated the disequilibrium chemistry of the warm exo-Neptune GJ 3470b, using a grid of disequilibrium models with various metallicities, thermal profiles, incident ultraviolet fluxes, and eddy diffusion coefficients (specifying the strength of atmospheric mixing) to determine the possible sensitivity of the atmospheric composition to these parameters. They find that, like GJ 1214b and GJ 436b, methane is favored over CO as the dominant atmospheric constituent, except when both metallicities (up to 100× solar) and temperatures are high.

Conspicuously absent from the current literature is a study of how ion chemistry affects the gas-phase composition and possible formation of hazes on super-Earths and exo-Neptunes. Given that ion chemistry initiates the formation of high-molecular-weight organics in the N_2_- and CH_4_-rich upper atmosphere of Titan (Waite et al. 2007), it seems likely that ion chemistry would be interesting on intermediate-sized exoplanets (especially the cooler ones).

### 5.3 Clouds and Hazes in Exo-Neptune and Super-Earth Atmospheres

Basic concepts of cloud and haze formation in extrasolar-planet atmospheres are discussed in Sect. 4.5. The same physics and chemistry that was described for giant planets is relevant to intermediate-sized planets, but the different starting ingredients can lead to different possible aerosol compositions. Morley et al. (2013) provide a good discussion of the possible equilibrium condensates along the standard cloud condensation sequence for H_2_-rich super-Earths and exo-Neptunes. In order from the hottest to the coldest dominant condensates, H_2_-rich atmospheres could typically contain equilibrium clouds of Al-Ca-Ti oxides and silicates, Fe metal, Mg silicates, Cr metal, MnS, Na_2_S, ZnS, KCl, NH_4_H_2_PO_4_, H_2_O, NH_4_SH, NH_3_, and CH_4_. For a solar-metallicity atmosphere, the total available mass for some of these clouds (e.g., ZnS, KCl) is pretty sparse, suggesting that they would not be very optically thick in the vertical; however, Morley et al. (2013) demonstrate that this conclusion changes as the metallicity is increased. They find that ZnS and KCl clouds could obscure the transit spectra of a metal-rich GJ 1214b if the particles are lofted to sufficiently high altitudes and have sedimentation times that are sufficiently long (e.g., due to small particle sizes).

Other possibilities for aerosols on moderately H_2_-rich planets include high-molecular-weight organics or “soots” that form through photochemical processes (e.g., Zahnle et al. 2009a; Moses et al. 2011; Miller-Ricci Kempton et al. 2012; Morley et al. 2013), or graphite that is stable in equilibrium for metal-rich atmospheres or ones with high C/O ratios (Moses et al. 2013a). Graphite can also be stable for a range of other compositions relevant to outgassed atmospheres of intermediate-sized planets (Schaefer and Fegley 2009, 2010), as can various other equilibrium condensates, such as alkali salts (Schaefer et al. 2012). Formation of sulfuric acid (H_2_SO_4_) clouds is likely through photochemical processes under a wide variety of conditions for high-metallicity or CO_2_-rich atmospheres that have sufficient SO_2_ (e.g., Schaefer and Fegley 2011; Schaefer et al. 2012; Hu et al. 2013), and elemental sulfur aerosols (e.g. S8) can be photochemically produced (e.g., Hu et al. 2013). However, the detailed formation pathways of photochemical hazes within the diverse atmospheres of intermediate-size planets has received relatively little attention and is still poorly understood.

## 6 Observational Inferences of Chemical Compositions

The last decade has witnessed substantial progress in observational inferences of chemical species in the atmospheres of giant exoplanets through a variety of methods. The planets for which such detections have been made are mostly hot giant planets, either in close-in orbits (i.e. ‘hot Jupiters’) or directly-imaged young objects at large orbital separations. The methods employed include differential photometry and spectroscopy of transiting hot Jupiters, high-resolution Doppler spectroscopy of transiting and non-transiting hot Jupiters, and high-resolution spectroscopy of directly-imaged planets. Whereas atomic species have been detected across the ultraviolet and visible, molecular species have been detected primarily in the near infrared. These detections were made thanks to pioneering observations using *Spitzer, HST*, and ground-based facilities. In what follows, we review the developments in each of these various areas.

### 6.1 Chemical Detections via Transit Spectroscopy

Atmospheric observations have been reported for over 50 transiting exoplanets to date, mostly as broadband/narrowband photometry or low resolution spectra. But, given the limited spectral resolution of the observations of most planets, molecular compositions have been inferred for only a few exoplanets to date. The inferred molecules typically include the most abundant and spectroscopically dominant molecules expected in hot atmospheres e.g. H_2_O, CO, CH_4_, and CO_2_. On the other hand, inferences have also been made of several atomic species and the presence of clouds/hazes in some atmospheres.

#### 6.1.1 Detections of Atomic Species

In high-temperature gas giant atmospheres (*T* ≳ 1000 K) in chemical equilibrium, alkali metals sodium (Na) and potassium (K) are expected to exist predominantly in atomic form thereby causing significant absorption in the visible via strong resonance lines at 589 nm and 770 nm, respectively (Seager and Sasselov 2000; Brown 2001). The strong line cores and wide pressure broadened wings are observable in optical transmission spectra of hot Jupiters. Thus the first Na detection was made using a visible band transmission spectrum of the hot Jupiter HD 209458b obtained using the HST STIS spectrograph (Charbonneau et al. 2002). However, while unambiguous, the spectrum revealed lower Na absorption than predicted by equilibrium models, indicating either lower atomic abundances than assumed or non-equilibrium processes such as rainout of condensed species and photoionization of Na/K (Fortney et al. 2003; Barman 2007).

The Na resonance doublet was also detected from ground in the optical transmission spectrum of the hot Jupiter HD 189733b (Redfield et al. 2008; Wyttenbach et al. 2015). Additionally, HST STIS observations of the transmission spectrum of HD 189733 also detected the Na absorption line core (Huitson et al. 2012). However, the ensemble of observations spanning UV to visible showed that the Na line is significantly weaker than that observed for HD 209458b, with a clear lack of broad line wings. Besides the weak Na I line, the spectrum was found to be largely featureless with a blue-ward slope which was consistent with the presence of strong scattering due to a thick haze of condensate grains (Vidal-Madjar et al. 2011; Pont et al. 2013). In recent years, Na I has been inferred in visible transmission spectra of a few other hot Jupiters, e.g. XO-2b (Sing et al. 2012), WASP-17b (Wood et al. 2011; Zhou and Bayliss 2012), HAT-P-1b (Nikolov et al. 2014), and WASP-12b (Burton et al. 2015).

Visible transmission spectra of hot Jupiters have also revealed other atomic species. Similar to Na, several studies have also detected the K resonance double at 770 nm using transmission spectra of hot Jupiters from both space and ground-based instruments, e.g., XO-2b (Sing et al. 2011a), and HD 80606 (Colón et al. 2012), HAT-P-1b (Wilson et al. 2015), WASP-31b (Sing et al. 2015). Other atomic species inferred in the visible include H *α* at 656 nm (Jensen et al. 2012), Ca and Sc (Astudillo-Defru and Rojo 2013)

Several atomic species have also been detected in the exospheres of hot Jupiters using UV transmission spectroscopy. The first such observations in Ly *α* revealed an extended envelope of escaping H in the hot Jupiter HD 209458b (Vidal-Madjar et al. 2003). Subsequent observations have revealed a rich population of atomic species in several hot Jupiter exospheres, e.g. H (Lecavelier Des Etangs et al. 2010; Bourrier et al. 2013), O and C (Vidal-Madjar et al. 2003), Si (Linsky et al. 2010; Schlawin et al. 2010), Mg (Fossati et al. 2010; Vidal-Madjar et al. 2013). Most recently, Ehrenreich et al. (2015) reported a detection of escaping atomic hydrogen in the exosphere of the hot Neptune GJ 436b.

#### 6.1.2 Early Molecular Inferences

Early inferences of molecules in exoplanetary atmospheres were based on few channels of photometry or low-resolution spectra obtained using then available instruments on *Spitzer* and *HST*. For example, some early studies used 2–3 near-infrared photometric observations in transmission to infer the presence of H_2_O at the day-night terminator regions of HD 209458b (Barman 2007) and HD 189733b Tinetti et al. (2007), but cf. Beaulieu et al. (2008) and Désert et al. (2009). Early attempts were also made to detect molecules in a handful of hot Jupiters using infrared spectroscopy with *HST* and *Spitzer*. For example, several studies used near-infrared spectra of hot Jupiters in transmission and/or emission obtained with the *HST* NICMOS instrument (1.8–2.3 μm) to report detections of H_2_O, CH_4_, CO, and/or CO_2_ in the hot Jupiters HD 189733b (Swain et al. 2008, 2009b; Madhusudhan and Seager 2009), HD 209458b (Swain et al. 2009a), and XO-1b (Tinetti et al. 2010). However, the uncertainties on the NICMOS observations have since been extensively debated in the literature leading to different molecular detection significances claimed by different teams, ranging from confident detections to no detections at the nominal 3-*σ* significance (Gibson et al. 2011, 2012; Waldmann et al. 2013; Swain et al. 2014).

Similar molecular inferences were also made using observations over a longer spectral baseline using *Spitzer* photometry and spectroscopy. Grillmair et al. (2008) used *Spitzer IRS* spectroscopy to infer the presence of H_2_O in the dayside atmosphere of HD 189733b using chemical equilibrium models (also see Todorov et al. 2014), whereas (Madhusudhan and Seager 2009) were able to place only an upper-limit on the possible abundances of H_2_O using the same dataset. Using six-channel photometric and/or spectroscopic observations from *Spitzer* and/or *HST*, Madhusudhan and Seager (2009) also inferred the presence of H_2_O, CH_4_, CO, and/or CO_2_ in the hot Jupiters HD 189733b and HD 209458b. However, several of the *Spitzer* photometric observations have also since been revised drastically (Knutson et al. 2012; Diamond-Lowe et al. 2014), thanks to our improved understanding of the instrumental systematics and, hence, the observational uncertainties (but cf. Hansen et al. 2014). Therefore, early inferences based on such observations are not currently substantiated.

The above early inferences, were followed by more molecular inferences from multiband photometry for smaller and/or more distant planets. Stevenson et al. (2010) and Madhusudhan and Seager (2011) used six-channel *Spitzer* photometry in thermal emission to infer the presence of CO and/or CO_2_ and the absence of CH_4_ in the dayside atmosphere of the hot Neptune GJ 436b. Given the relatively lower equilibrium temperature of the planet, the presence of CO and absence of CH_4_ was suggested to be indicative of strong chemical disequilibrium and high metallicity in the atmosphere. While these data, and hence the molecular inferences, were originally contested by Beaulieu et al. (2011), recent comprehensive and independent analyses by Lanotte et al. (2014) support the original inferences of a methane poor and CO/CO_2_ rich atmosphere in GJ 436b.

Another major observational advancement that followed was the possibility of detecting thermal emission from hot Jupiters in the near-infrared from ground (e.g. Sing and López-Morales 2009; Croll et al. 2010). The advantage of these measurements was that their spectral range, between ~0.9 μm and 2.3 μm, complemented that typically available from *Spitzer* photometry (3.6–8 μm) to provide a long spectral baseline to retrieve chemical species from the combined data. In the first such instance, observations of thermal emission in seven photometric bandpasses (between ~1.2–8 μm) were used to infer the presence of significant CO and CH_4_ and lack of H_2_O, and hence a C/O ratio ≥ 1, in the hot Jupiter WASP-12b (Madhusudhan et al. 2011a). Again, the *Spitzer* photometric observations were a subject of intense debate initially (Crossfield et al. 2012; Cowan et al. 2012a). However, subsequent multi-epoch *Spitzer* observations in the same bandpasses have reinstated the original data and the conclusions (Stevenson et al. 2014a) for the dayside atmosphere of WASP-12b.

#### 6.1.3 Molecular Detections with HST WFC3

**Fig. 9:**
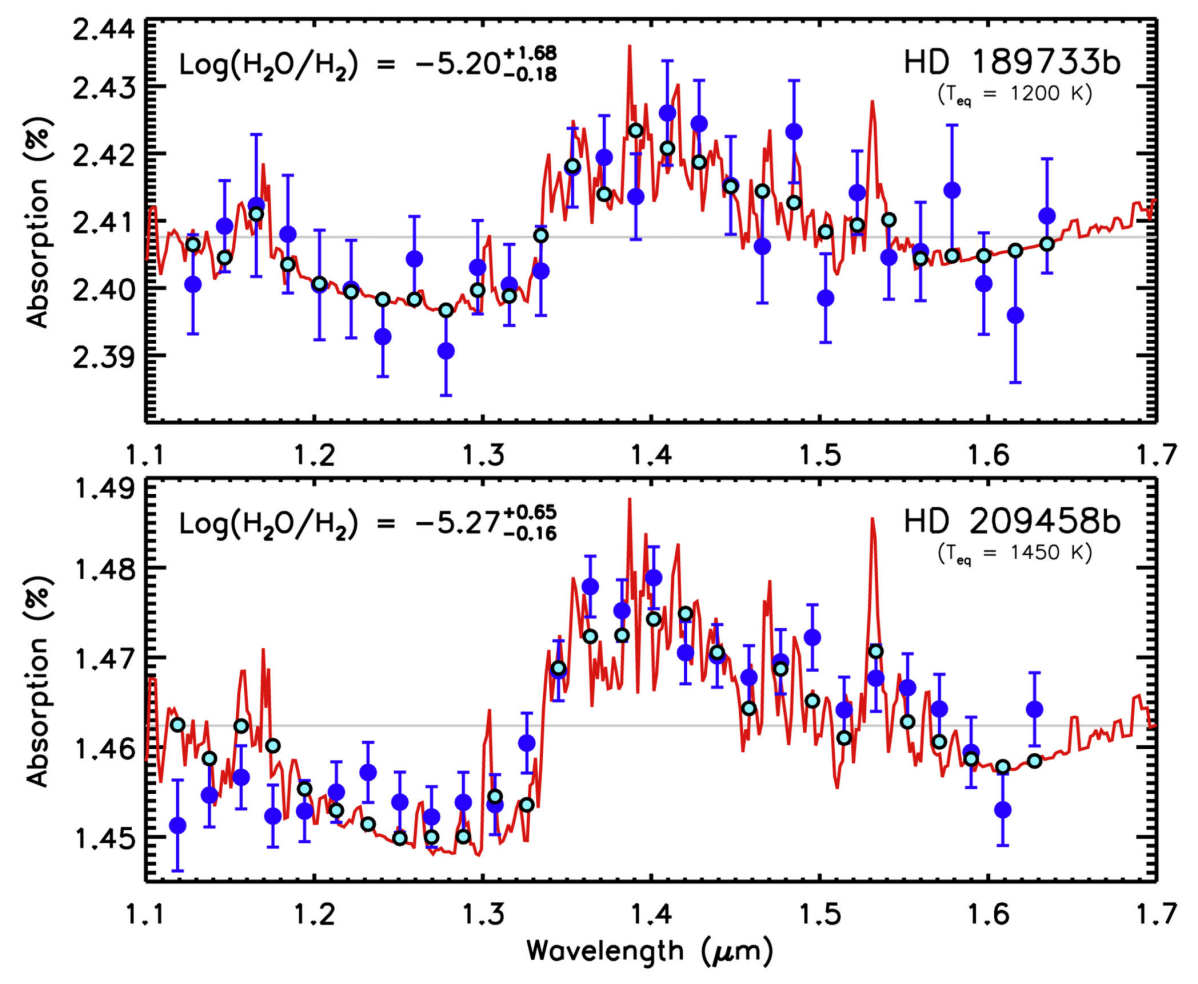
Transmission spectra of two hot Jupiters observed with HST WFC3 (adapted from Madhusudhan et al. 2014b). The *vertical axis* shows absorption (transit depth). The *blue circles* show the data: HD 209458b from Deming et al. (2013) and HD 189733b from McCullough et al. (2014). The *red curves* shows the best-fit model spectra, and the *cyan circles* show the models binned to the same resolution as the data. The *peaks* around 1.4 μm show H_2_O absorption

**Fig. 10:**
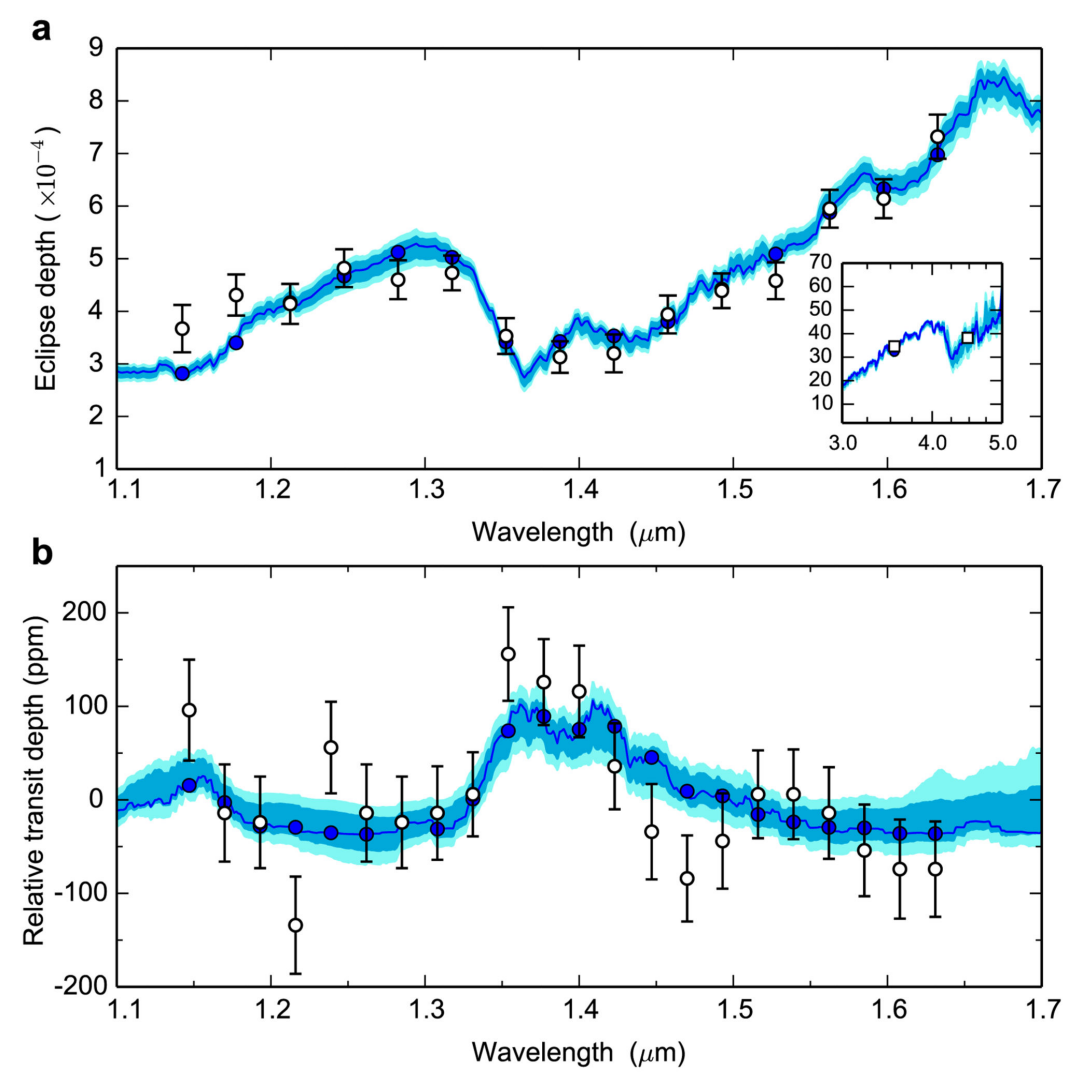
Spectra of the hot Jupiter WASP-43b observed with HST WFC3 (from Kreidberg et al. 2014b). *Top*: eclipse spectrum. *Bottom*: transmission spectrum. In both cases, the *black circles with error bars* show the data and *solid curves* show best-fit model spectra. The *filled dark blue circles* show the best-fit model binned to the same resolution as the data

In recent years, near-infrared *HST* spectroscopy has led to detections of H_2_O in the atmospheres of several transiting hot Jupiters. This has been made possible by the *HST* Wide Field Camera 3 (WFC3) spectrograph (McCullough and MacKenty 2012) which operates in the near-infrared range 1.1–1.8 μm, where water vapor has a strong absorption band, as shown in Figs. 9 and 10. Since H_2_O is one of the most abundant molecules expected in giant exoplanetary atmospheres, the HST WFC3 instrument provides a unique opportunity to constrain H_2_O abundances in such atmospheres. As shown in Fig. 9, Deming et al. (2013) reported the first WFC3 detection of H_2_O in the transiting hot Jupiter HD 209458b using transmission spectroscopy, which probed the atmosphere at the day-night terminator region of the planet. The observations led to a clear 10-*σ* detection of H_2_O in a single transit thanks to the high brightness of the host star (*V* = 7.6).

Several subsequent studies reported single-event transmission spectra of transiting hot Jupiters orbiting less brighter stars with varied levels of success in H_2_O detections. Some of the notable H_2_O detections, albeit less significant than that for HD 209458b, were reported for hot Jupiters WASP-17b (Mandell et al. 2013), WASP-19b (Huitson et al. 2013), HAT-P-1b (Wakeford et al. 2013), WASP-12b (Stevenson et al. 2014a,b), and HD 189733b (McCullough et al. 2014), and a hot Neptune HAT-P-11b (Fraine et al. 2014), whereas similar observations of several other planets resulted in featureless spectra within the observed uncertainties (e.g. Swain et al. 2013; Mandell et al. 2013; Ranjan et al. 2014). These efforts demonstrate that single transits are inadequate to make high-confidence H_2_O detections for most hot Jupiters currently known with the exception of those transiting the brightest stars such as HD 209458b. Consequently, more recent observations using multiple visits to reduce the observational uncertainties have led to good quality transmission spectra with clear H_2_O detections for hot Jupiters orbiting even moderate brightness stars (Kreidberg et al. 2014a, 2015).

Several studies have also used *HST* WFC3 to observe thermal emission spectra of hot Jupiters at secondary eclipse. In addition to the presence of molecular abundances, thermal emission spectra also provide constraints on the disk-averaged temperature profile of the dayside atmosphere. However, with the exception of a few cases WFC3 observations to date have generally revealed thermal spectra with subdued molecular features, if any. The first such spectrum was observed for the very hot Jupiter WASP-12b (*T_eq_* ~ 2500 K) which revealed a featureless thermal spectrum consistent with a blackbody spectrum (Swain et al. 2013; Madhusudhan 2012; Stevenson et al. 2014a). Considered on its own, the spectrum is consistent with an isothermal temperature structure in the atmosphere. On the other hand, considering together with existing photometric observations in the range 0.9–8 μm the observations require an atmosphere with a temperature profile decreasing outward and significantly depleted in H_2_O as expected for a carbon-rich atmosphere, C/O ≥ 1 (Madhusudhan 2012; Stevenson et al. 2014a). Subsequent studies have observed WFC3 thermal emission spectra for several other hot Jupiters (Wilkins et al. 2014; Ranjan et al. 2014; Stevenson et al. 2014a; Crouzet et al. 2014; Haynes et al. 2015).

Robust detections of molecular features in thermal emission spectra using HST WFC3 have been reported for the dayside atmospheres of only two hot Jupiters to date. Stevenson et al. (2014c) and Kreidberg et al. (2014b) reported unambiguous detection of H_2_O in the dayside atmosphere of WASP-43b, as shown in Fig. 10. Most recently, Haynes et al. (2015) reported a detection of H_2_O and the first detection of TiO in the dayside atmosphere of the extremely irradiated hot Jupiter WASP-33b.

#### 6.1.4 Abundance Estimations Using Retrieval Methods

Going beyond detecting the presence of molecules, the exoplanetary spectra discussed above have also been used to derive the molecular abundances which in turn can be used to constrain atmospheric processes, elemental abundances, bulk compositions, and formation conditions. Molecular abundances are derived from exoplanetary spectra using detailed atmospheric retrieval methods which lead to joint statistical constraints on the chemical composition and temperature profile of an exoplanetary atmosphere given an observed spectrum; see e.g. Madhusudhan et al. (2014c) for a detailed review of atmospheric retrieval techniques for exoplanets. Atmospheric retrieval methods for exoplanets typically comprise of a 1-D atmospheric model coupled with an optimization algorithm to estimate the free parameters of the model given the data. The molecular abundances and the pressure-temperature (*P*-*T*) profile are free parameters in the model; typically there are over ten free parameters depending on the number of molecules included and the adopted parametrization of the temperature profile. For the optimization algorithm, a number of methods have been tried over time ranging from grid-search in the early days (Madhusudhan and Seager 2009) to Bayesian approaches such as the Markov Chain Monte Carlo (MCMC) method in subsequent years (Madhusudhan et al. 2011a; Line et al. 2012; Benneke and Seager 2013; Waldmann et al. 2015) and gradient descent methods (Lee et al. 2012).

Chemical abundances have been retrieved for several giant exoplanets to date. The majority of these planets are transiting hot Jupiters for which high-precision transmission and/or emission spectra have been obtained using the HST WFC3 spectrograph and, in some cases, Spitzer and/or ground-based photometry. Initial statistical constraints on atmospheric abundances (e.g. Madhusudhan and Seager 2009; Lee et al. 2012; Line et al. 2012) in the pre-WFC3 era were based on photometric data and/or low-resolution spectra, the data and uncertainties of which have since been revised as discussed above. On the other hand, while robust HST WFC3 spectra have been observed for over a dozen planets now, most of them have uncertainties large enough to be consistent with featureless spectra (Mandell et al. 2013; Ranjan et al. 2014). Therefore, in what follows we review only a selection of observed transiting exoplanets for which non-flat spectra have been observed at ≥3-*σ* significance and for which abundance constraints have been reported. This includes five transiting hot Jupiters (HD 209458b, HD 189733b, WASP-12b, WASP-33b, WASP-43b), and a hot Neptune (HAT-P-11b).

Currently, the molecule with the best abundance estimates available is H_2_O, thanks to high-precision spectra with HST WFC3. As discussed above, the HST WFC3 G141 grism with a spectral range of 1.1–1.8 μm covers a strong H_2_O absorption band near 1.4 μm thereby allowing H_2_O measurements from exoplanetary spectra. H_2_O has been detected in all the six giant exoplanets listed above, albeit with different abundances. One important feature in almost all the transmission spectra with robust H_2_O detections to date is that the amplitude of the H_2_O absorption feature is typically smaller than model predictions for a clear (i.e. free of clouds/hazes) solar-abundance atmosphere. This was first noted for the hot Jupiter with the most-precise transmission spectrum, HD 209458b (Deming et al. 2013). For such spectra, assuming a clear atmosphere results in an H_2_O abundance at the day-night terminator that is markedly sub-solar: 20–100× sub-solar for HD 209458b and 3−200× sub-solar for HD 189733b (Madhusudhan et al. 2014b). However, the derived low H_2_O abundances are degenerate with the possibility of clouds or hazes at the terminator in these atmospheres (Sing et al. 2013; Benneke 2015), i.e. the same spectra can be explained with solar or supersolar abundances if high altitude clouds or hazes are allowed in the atmosphere. Transmission spectra of several other hot Jupiters were also found to be consistent with solar abundances when clouds/hazes are invoked or when the uncertainties are large enough to preclude high-precision abundance estimates (Huitson et al. 2013; Line et al. 2013; Barstow et al. 2014; Benneke 2015). Therefore, the possibility of clouds present a fundamental challenge in deriving chemical abundances from transmission spectra, which may be mitigated if optical transmission spectra are available (discussed in the following section) and/or where abundance estimates are available for the dayside atmospheres using thermal spectra.

Constraints on molecular abundances have also been reported in the dayside atmospheres of hot Jupiters using thermal emission spectra. An early example in this regard is the hot Jupiter WASP-12b, for which broadband photometric observations originally suggested a 3-*σ* upper-limit on the H_2_O abundance that was ~100× sub-solar, and implied a C/O ratio of ≥ 1 (Madhusudhan et al. 2011a). While the veracity of the Spitzer photometric observations of WASP-12b were a subject of substantial debate in the intervening years (Cowan et al. 2012a; Crossfield et al. 2012), latest multi-epoch Spitzer observations together with high-precision HST WFC3 spectra are consistent with the lack of significant H_2_O in the dayside atmosphere of WASP-12b (Madhusudhan 2012; Stevenson et al. 2014b). Most recently, H_2_O was detected at the day-night terminator region of the planetary atmosphere using a HST WFC3 transmission spectrum, however the abundance of H_2_O detected was subject to model assumptions as discussed above (Kreidberg et al. 2015). Similar to the other datasets discussed above, a cloud-free atmosphere required a sub-solar H_2_O abundance whereas the inclusion of clouds/hazes and/or the imposition of chemical equilibrium allowed the data to be consistent with solar abundance H_2_O.

In another example, joint constraints on the H_2_O abundance at both the terminator as well as the dayside atmosphere were obtained using transmission and emission spectra for the hot Jupiter WASP-43b resulting in an H_2_O abundance of 0.3–3× solar, i.e. both sub-solar and marginally super-solar abundances are allowed by the data (Kreidberg et al. 2014b). Finally, abundance estimates for the dayside atmosphere of WASP-33b, the most irradiated hot Jupiter observed with HST WFC3 to date, also revealed a marginally sub-solar (~0.5×) H_2_O abundance (Haynes et al. 2015). HST WFC3 thermal spectra for a few other hot Jupiters have also revealed relatively low-amplitude or non-existent H_2_O features, hinting at the possibility of low H_2_O abundances (e.g. Wilkins et al. 2014; Crouzet et al. 2014). While in principle clouds or hazes could also cause featureless (blackbody-like) spectra in thermal emission, the observed brightness temperatures of the spectra are quite high, indicative of the lower atmospheres, implying that they were unlikely to have been impeded by clouds/hazes in the atmosphere.

The observed molecular abundances can be used to constrain the O/H, C/H, and C/O ratio of the atmosphere (e.g. Madhusudhan et al. 2011a), which in turn are important to constrain exoplanetary formation conditions, as discussed in Sect. 6. However, estimating C/O ratios is non-trivial. Reliably estimating the C/O ratio for an exoplanetary atmosphere requires that the observations are able to constrain the molecular abundances of all the dominant O and C bearing molecules in a given atmosphere. As discussed in Sect. 3, for hot giant exoplanets, the dominant molecules are H_2_O, CO, CH_4_, and to a lesser extent CO_2_, C_2_H_2_, and HCN, depending on the C/O ratio. Simultaneously constraining the abundances of all these molecules requires a long spectral baseline with observable bandpasses where these molecules have strong spectral features. This is, in principle, possible with the combination of HST WFC3 and the Spitzer IRAC Channels 3.6–8 μm (Madhusudhan 2012). Indeed, such constraints were placed for the few planets where such data are available in thermal emission allowing constraints on the C/O ratios of their dayside atmospheres. While a carbon-rich dayside atmosphere (C/O ≥ 1) has been reported for the hot Jupiter WASP-12b (Madhusudhan 2012; Stevenson et al. 2014b), oxygen-rich dayside atmospheres consistent with a solar C/O of 0.5 were reported for the hot Jupiters WASP-43b (Kreidberg et al. 2014b) and WASP-33b (Haynes et al. 2015).

On the other hand, determining the C/O ratio at the day-night terminator from transmission spectra is challenging and prone to misinterpretation. One of the main reason is that typically only WFC3 spectra are available in transmission, implying that stringent constraints are possible only on the H_2_O abundance. In principle, the abundances of CH_4_ and HCN may also be constrained in the WFC3 spectral range, however the most dominant O and C bearing molecule in hot Jupiters is CO, which is unconstrained by WFC3 observations. Consequently, with WFC3 data alone the C/H and hence the C/O ratio cannot be reliably constrained. For example, if a sub-solar H_2_O abundance is observed in a hot Jupiter (assuming a cloud-free atmosphere), the low H_2_O can be caused either by a low overall metallicity (i.e. low O/H) with a solar-like C/O ratio or by a high metallicity but a high C/O ratio (~1), depending on the temperature. Therefore, studies which attempt to derive C/O ratios from WFC3 transmission spectra alone do so by enforcing constraints of equilibrium chemistry or temperature profiles in radiative equilibrium or both (Benneke 2015; Kreidberg et al. 2015), neither of which is strictly justified at the day-night terminator regions of strongly irradiated atmospheres with strong atmospheric circulation. On the other hand, the presence of clouds only makes the inference more degenerate. Therefore, multi-wavelength observations besides WFC3 spectra are essential for reliable constraints on C/O ratios from transmission spectra.

#### 6.1.5 Degeneracy with Clouds and Hazes

The presence of high-temperature condensates as hazes and clouds in hot exoplanetary atmospheres may contribute significantly to the optical depth in transmission spectra (Seager and Sasselov 2000; Fortney 2005). Therefore, determinations of chemical compositions from transmission spectra can be strongly degenerate with the presence of clouds or hazes in the atmospheres. In transmission spectra, the amplitude of a spectral feature is directly proportional to the height of the atmospheric annulus through which the star light traverses. The presence of an opaque cloud deck at a given height in the atmosphere means that only the layers above the cloud deck contribute to the transmission spectrum. Thus for a cloud deck located high enough in the atmosphere the amplitude of a spectral feature can be significantly diminished thereby confounding estimates of the corresponding molecular abundances.

High-precision observations of several transiting exoplanets have revealed such spectra with diminished features. At the extreme end are recent HST WFC3 observations of remarkably flat spectra, despite extremely high precisions, for super-Earths GJ 1214b (Kreidberg et al. 2014a) and HD 97658b (Knutson et al. 2014b), exo-Neptune GJ 436b (Knutson et al. 2014a), and exo-Uranus GJ 3470b (Ehrenreich et al. 2014). The featureless transmission spectra in these low-mass, low-temperature (*T* below ~800 K), planets have been attributed to the presence of thick high-altitude clouds with cloud-top pressures below a mbar. On the other hand, even for hot Jupiters (*T ~* 1200–3000 K) which show unambiguous H_2_O features the amplitude of the spectral features are significantly diminished, corresponding to only ~1–2 scale heights (Deming et al. 2013; Kreidberg et al. 2015), compared to expectations of 5–8 scale heights (Madhusudhan and Redfield 2015). These diminished spectral features may be attributed to the presence of clouds/hazes in these hot Jupiters, but as discussed above the same features may also be attributed to significantly lower H_2_O abundances than previously expected (Madhusudhan et al. 2014c; Benneke 2015).

**Fig. 11:**
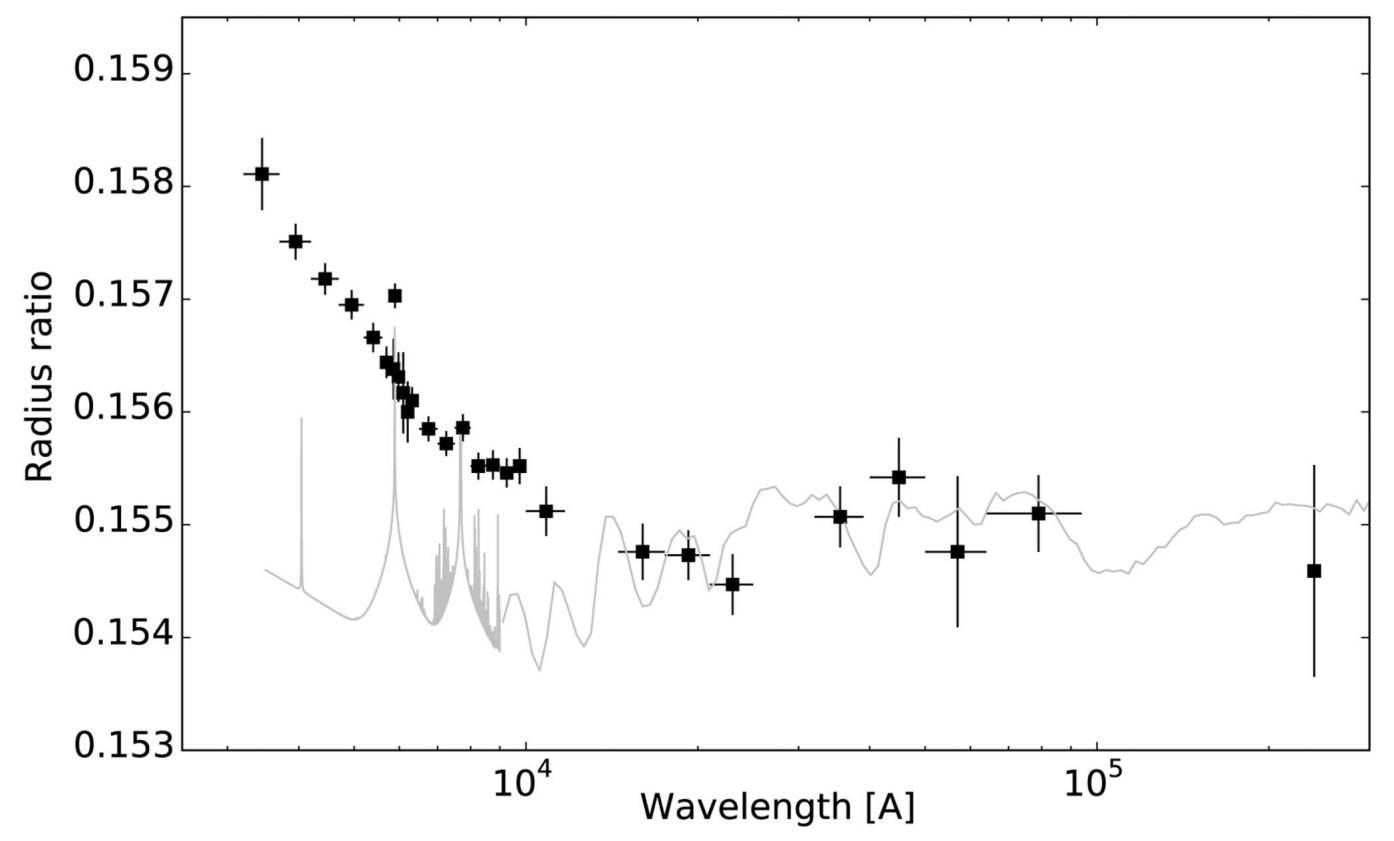
Transmission spectrum of the hot Jupiter HD 189733b from UV to near-infrared (from Pont et al. 2013). The *black circles with error bars* show the data available at that time, observed using multiple instruments on *HST* and *Spitzer*. The *grey curve* shows a model spectrum for a dust-free atmosphere. The *steep blue-ward rise* in the spectrum was indicative of strong scattering due to haze/dust in the atmosphere which was not consistent with the expected model spectrum for a haze-free atmosphere

Scattering due to haze particles have also been inferred from visible transmission spectra of hot Jupiters which show an inverse power-law dependence on wavelength with parameters different from that due to pure gaseous Rayleigh scattering. The archetype for such inferences is the hot Jupiter HD 189733b (Lecavelier Des Etangs et al. 2008b; Pont et al. 2008; Sing et al. 2011b) where observations with HST revealed a power law slope in the UV and optical indicating the presence of hazes, as shown in Fig. 11. Similar inferences have been made for several other hot Jupiters in recent years (Sing et al. 2013, 2015; Nikolov et al. 2015). A potential degeneracy in such inferences arises from the fact that the presence of star spots may also cause similar optical spectra with inverse power law slopes (e.g. McCullough et al. 2014). However, in addition to the power-law slopes observations of partially muted alkali (Na and/or K) absorption lines in the optical have also indicated the presence of cloud decks in some atmospheres (Sing et al. 2013, 2015).

#### 6.1.6 Albedos and Optical Phase Curves

The presence of clouds/hazes have also been inferred using reflection spectra of hot Jupiters obtained at occultation. Early observations presented little evidence for clouds/hazes and suggested that hot Jupiters are dark, potentially due to strong absorption in the optical due to alkali metals Na and K. Such studies reported rather low geometric albedos for several hot Jupiters in the optical, e.g. 0.038 ± 0.045 for HD 209458b (Rowe et al. 2008) and 0.0136 ± 0.0027 for TrES-2 (Kipping and Spiegel 2011). However, recent new observations are providing evidence for high geometric albedos and evidence for clouds/hazes in dayside spectra of hot Jupiters. Demory et al. (2011, 2013) reported a high visible geometric albedo of 0.35 ± 0.02 for the hot Jupiter Kepler-7b observed in the Kepler photometric bandpass (~0.4–1.0 μm). Furthermore, based on the asymmetry in the visible phase curve they also suggested the presence of an inhomogeneous cloud cover in the atmosphere of Kepler-7b. In another study, Evans et al. (2013) reported a low-resolution albedo spectrum of the hot Jupiter HD 189733b in the 0.29–0.57 μm range using HST which nominally showed the albedo increasing blue-ward and inferred the presence of reflective clouds in the atmosphere; also see Barstow et al. (2014). Most recently, Martins et al. (2015) reported a high-resolution spectroscopic detection of reflected light for the non-transiting planet 51 Peg b which suggests a high geometric albedo (~0.5) of the planet. Several other recent studies have used photometric observations to understand the trends and/or report constraints on albedos and/or optical phase curves for a sizeable sample of exoplanets (e.g. Heng and Demory 2013; Esteves et al. 2015; Hu et al. 2015a; Schwartz and Cowan 2015).

While it is apparent from the above observations that clouds/hazes likely exist in at least a subset of irradiated atmospheres, the compositions of cloud forming species in currently unconstrained. However, as discussed in Sects. 3–4, theoretical studies over the past decade show that a wide range of high-temperature refractory condensates can form in such atmospheres in the 500–2000 K range. Such condensates range from NaCl, KCl, ZnS for *T* below 1000 K to silicates, Fe, and Al_2_O_3_ at ~1000–2000 K temperatures (e.g. Sudarsky et al. 2003; Marley et al. 2013; Morley et al. 2013).

### 6.2 Molecular Detections via High-Resolution Doppler Spectroscopy

Robust detection of molecules in exoplanetary atmospheres have also been made using high-resolution Doppler spectroscopy in the near infrared. This technique involves the detection of molecular lines in the planetary spectrum that are shifted in wavelength due to the radial velocity of the planet (Brogi et al. 2012; Birkby et al. 2013). For close-in hot Jupiters, the orbital velocities are ~ kms^−1^ whereas the stellar orbital velocities are significantly lower (e.g. below ~100 ms^−1^). Thus the spectrally shifted molecular lines of the planetary atmosphere are easily identifiable compared to those in the stellar spectrum as well as those in the telluric spectrum which is static. A template planetary spectrum including the sought after molecule is cross-correlated with the observed spectrum to detect the Doppler shift in the molecular lines with phase thereby revealing the presence of the molecule. Critical to this method, however, is the high resolution of the observed spectrum so that individual molecular lines can be resolved. Consequently, most of the success in this area has been achieved using the CRIRES instrument on the Very Large Telescope (VLT) with a spectral resolving power of 100000 in the near-infrared (Snellen et al. 2010). Moreover, as the planetary signal diminishes with the increased resolution the method has been successfully applied only to planets orbiting the brightest stars.

This method has been used to robustly detect CO and H_2_O in several hot Jupiter atmospheres. The detections of these particular molecules are favored by the fact that they are expected to be the most dominant O and C bearing molecules in hot Jupiter atmospheres, especially for *T >* 1300 K, and also have detectable absorption lines in the range ~2–3 μm where these atmospheres are most conducive to observe from ground. This wavelength range offers the optimal conditions because in the near-infrared the planet-star flux ratio increases with wavelength, however the background noise also increases with wavelength; therefore the K-band (around 2.1 μm) typically offers an optimal choice for ground based transit/eclipse spectroscopy. Snellen et al. (2010) reported the first detection of CO using this technique in the hot Jupiter HD 209458b in transit which in turn also led to a constraint on the day-night wind velocity in the planetary atmosphere. Subsequent observations have led to the detection of CO and H_2_O in the dayside atmospheres of several transiting and non-transiting planets: *τ*Boo (Brogi et al. 2012; Rodler et al. 2012; Lockwood et al. 2014), 51 Peg b (Brogi et al. 2013), HD 189733b (de Kok et al. 2013; Birkby et al. 2013; Rodler et al. 2013), and HD 179949b (Brogi et al. 2014).

### 6.3 Directly Imaged Gas Giants

Direct imaging offers another avenue to characterize atmospheric compositions of exoplanets and works preferentially for a complementary region in planetary parameter space. Exoplanets detected to date via direct imaging are all young gas giants at large orbital separations. Their young ages (below ~100 Myr) implies high effective temperatures (~1000 K) and, hence, high planet-star flux contrasts in the near-infrared. On the other hand, their large orbital separations (beyond ~10 AU), which are set by the inner working angles of the instruments used, help in minimizing the stellar glare. Together these factors make young gas giants particularly favorable to near-infrared spectroscopy via direct imaging.

The advantage of direct imaging is that the detection of a planet simultaneously results in observation of its atmospheric thermal emission spectrum, i.e. atmospheric characterization can be pursued simultaneously with detection. This is in contrast to transiting exoplanets where the detections are generally made using photometric transit surveys and RV confirmations, while atmospheric spectra are obtained for optimal targets using follow-up observations with specialized instruments. On the other hand, the challenge with directly imaged planets is that generally only the atmospheric spectrum is available with little information about any other planet property; the mass, radius, and hence gravity, temperature, age, are all unknowns in modeling the planets. Therefore, typically, planetary evolution models are required along with atmospheric models to robustly constrain the atmospheric and physical parameters of the planets. Moreover, spectra are available at only one point of the orbital phase which means thermal phase curves are not observable precluding constraints on atmospheric properties with longitude. Nevertheless, the possibility of high-resolution absolute near-infrared spectroscopy for directly-imaged planets means that chemical signatures can be robustly detected in emission spectra using ground-based instruments.

Chemical detections have been reported for a few directly-imaged exoplanets in recent years. Given the expected temperature range of young giant planets, the dominant molecular species expected are H_2_O, CO, and CH_4_. All these molecules have been detected in one or more directly-imaged planets. Most of the detections have been reported for planets in the well characterized and nearby HR 8799 system. H_2_O, CO, and CH_4_ have been unambiguously detected in the planet HR 8799b using high-resolution spectroscopy in the H and K band (Barman et al. 2011, 2015), though the CH_4_ absorption is weaker than expected from chemical equilibrium (Bowler et al. 2010; Barman et al. 2015). H_2_O and CO have also been detected in high-resolution spectra of HR 8799c (Konopacky et al. 2013). The presence of CH_4_ and other candidate molecules (e.g. NH_3_, C_2_H_2_, CO_2_, and/or HCN) have also been suggested using lower resolution spectra for the four planets in the HR 8799 system (Oppenheimer et al. 2013). Janson et al. (2013) reported an H-band detection of CH_4_ in GJ 504b. More recently, CO was also detected unambiguously in *β* Pic b (Snellen et al. 2014) which also provided the first measurement of the spin period of an exoplanet.

In addition to molecular detections, spectra of directly imaged planets have also been use to place constraints on their elemental abundance ratios just as have been pursued for transiting hot Jupiters. However, determining elemental abundance ratios for directly imaged planets is challenging since most of the planetary properties (e.g. mass, radius, gravity, temperature) are not known a priori and hence need to be fit for, often requiring evolutionary models, in addition to the usual complexities introduced by the presence of clouds. Nevertheless, several studies have attempted to fit static models to spectra of directly-imaged planets to report nominal constraints on the elemental abundances. Using a model grid to fit spectroscopic observations of HR 8799 c, Konopacky et al. (2013) suggested a C/O ratio of $\text{0}{\text{.65}}_{-0.05}^{\text{+0}.10}$ which is slightly higher than a solar value of 0.54. Lee et al. (2013) performed an atmospheric retrieval analysis for HR 8799b to suggest a high metallicity, with a mean molecular weight of 3.8 compared to 2.4 for solar abundance, and a C/O ratio ~1 in its atmosphere. More recently, Todorov et al. (2015) performed an atmospheric retrieval analysis to constrain the molecular abundances in the sub-stellar companion *κ* and b and reported an H_2_O abundance of ~10^−4^, nearly consistent with a solar abundance composition and those of several transiting hot Jupiters.

## 7 Implications for Formation Conditions of Exoplanets

Atmospheric elemental abundances of solar-system giant planets have led to important constraints on the origins of the solar system. In Jupiter’s atmosphere, the abundances of C, N, S, Ar, Kr, and Xe, relative to H have been measured to be 2–3× solar values (Owen et al. 1999; Atreya and Wong 2005) which suggest substantial accretion of solids during its formation, and have been used as evidence for its metal-rich interior and its formation by core accretion. However, the abundance of O is not known for Jupiter. Given the low temperatures (≲125 K) in Jupiter’s observable troposphere, H_2_O is condensed down to deeper layers of the atmosphere at pressures greater than ~10 bar. As the Galileo probe descended into Jupiter’s atmosphere, its last measurement showed an H_2_O abundance, and hence an O abundance, of 0.3× solar, which is 10× lower than the other elements (Atreya and Wong 2005). However, this measurement of Jupiter’s H_2_O abundance is considered to be a lower-limit since the region the probe entered is thought to be an anomalously dry spot in Jupiter’s atmosphere. On the other hand, indirect constraints based on kinetics models explaining the observed CO abundance suggest an O abundance of 0.3–7.3× solar (Visscher et al. 2010b), which allows for a wide range of C/O ratios.

Taking the currently available lower-limit on O at its true abundance, implying more carbon than oxygen, would require unusual formation conditions in the early solar system. Lodders (2004) studied the hypothetical possibility and suggested that tarry planetesimals may have dominated Jupiter’s accretion history, instead of planetesimals dominant in water-ice as expected from compositions of minor bodies in the solar system. Instead, using core accretion models Mousis et al. (2012) suggested that considering a solar-composition nebula predicts an O abundance of 3–7× solar. Therefore, a significantly lower observed O abundance would require substantial depletion of H_2_O ice in Jupiter’s formation region, unless the planetesimals are unusually carbon-rich. Thus, accurately measuring the O abundance is critical to constrain the formation conditions of Jupiter, and of the outer solar system in general. The H_2_O abundance is similarly unknown for any other giant planet in the solar system. The upcoming Juno mission to Jupiter (Matousek 2007), therefore, aims to constrain its O abundance by measuring the H_2_O abundance in Jupiter’s atmosphere. In particular, the H_2_O abundances play a central role in constraining exoplanetary formation conditions. Since O is cosmically the most abundant heavy element it is expected that H_2_O is one of the most dominant volatile in interstellar and planet-forming environments (van Dishoeck et al. 2014). Thus, the H_2_O abundances in giant planetary atmospheres are of fundamental importance to constrain the inventory of O that was available in planet’s formation environments.

The O/H and C/O ratios are easier to measure for hot giant exoplanets than they are for solar-system giant planets (Madhusudhan 2012). The vast majority of extrasolar gas giants known have *T* ~ 600–3000 K, thus hosting gaseous H_2_O in their observable atmospheres accessible to spectroscopic observations. Other detectable gases include CH_4_, CO, CO_2_, and NH_3_, depending on the temperature and incident irradiation. Measurements of such molecular abundances allow estimations of elemental abundances ratios involving H, C, O, and N. Such elemental abundances can in turn provide crucial clues regarding exoplanetary atmospheric processes, interior compositions, and formation mechanisms, just as pursued for solar system planets. This fortuitous opportunity makes hot giant exoplanets the perfect laboratories to investigate the origins of giant planets and the diversity of their atmospheres and interiors. Indeed, as discussed above, recent observations are already leading to unprecedented detections of chemical species in giant exoplanetary atmospheres. Such spectra have already led to clear H_2_O detections in atmospheres of several transiting hot Jupiters, as discussed above (e.g. Deming et al. 2013; Kreidberg et al. 2014b; Madhusudhan et al. 2014b). H_2_O, CO, and/or CH_4_ have also been detected using high-resolution ground-based spectroscopy of hot Jupiters (e.g. Brogi et al. 2014), as well as directly imaged planets (e.g. Konopacky et al. 2013). Upcoming large facilities such as the *JWST* and *E-ELT* will further revolutionize the field.

New studies are beginning to investigate the influence of formation and migration histories of giant exoplanets on their observable chemical compositions. As discussed in previous sections, observations are suggesting the possibility of carbon-rich (C/O ≥ 1) as well as oxygen-rich (C/O < 1) atmospheres in giant exoplanets (Madhusudhan et al. 2011a; Madhusudhan 2012) which are in turn motivating new ideas on the formation conditions of planetary systems. One of the key questions in this regard is about how C-rich gas giants can form around O-rich sun-like stars; the solar C/O is 0.5 (Asplund et al. 2009). As discussed above, an early investigation into this question was pursued in the context of Jupiter in the solar-system for which, as discussed above, only a lower limit on the O/H is known, which allows for a C/O > 1 in Jupiter. More recently, following the inference of C/O ≥ 1 in WASP-12b (Madhusudhan et al. 2011a), Öberg et al. (2011) suggested that C/O ratios in giant planetary envelopes depend on the formation location of the planets in the disk relative to the snow lines of major C and O bearing volatile species, such as H_2_O, CO, and CO_2_, since the C/O ratio of the gas approaches 1 outside the CO and CO_2_ snow lines. By pre-dominantly accreting such C-rich gas, more so than O-rich planetesimals, gas giants could, in principle, host C-rich atmospheres even when orbiting O-rich stars. On the other hand, it may also be possible that inherent inhomogeneities in the C/O ratios of the disk itself may contribute to higher C/O ratios of the planets relative to the host stars (Kuchner and Seager 2005; Madhusudhan et al. 2011b; Mousis et al. 2012; Moses et al. 2013b; Ali-Dib et al. 2014). The compositions of gas and solids accreted also depend on the physicochemical properties of the disk at the given location which are time-dependent (Helling et al. 2014; Marboeuf et al. 2014). Furthermore, some of the volatile elements can also be depleted due to dust formation and settling in the atmosphere (Moses et al. 2013b; Helling et al. 2014). These various scenarios predict different limits on the metallicities and C/O ratios of the giant planets, which high-precision observations of their atmospheres will be able to test in the near future.

Besides the various factors discussed above, the main formation mechanisms also contribute significantly to the planetary composition. Giant planets are thought to form via one of two primary mechanisms, core accretion (CI) versus gravitational instability (GI). In the CI model (Pollack et al. 1996), the planetary embryos start out as 10 Earth-mass cores in the protoplanetary disk that subsequently undergo runaway accretion of a large volume of gas and planetesimals to form a massive gaseous envelope. On the other hand, a GI in a young disk can cause rapid collapse of a large volume of ambient gas and solids to form a giant planet (Boss 2000). Both scenarios occur in planet-forming disks, but at different orbital separations. While CA is favored closer to the snow-line (within ~2–10 AU) because cores take too long to form at larger distances and only reach large masses after the disk has dispersed, GI is favored at larger distances (≳10 AU) where the disk can cool sufficiently on orbital timescales to fragment. In this regard, GI may be the favored mechanism for the formation of distant gas giants detected via direct imaging. However, because the different formation mechanisms are favorable at different orbital separations in the disk they may be expected to probe different chemical compositions in the disk. In principle, both CA and GI can cause significant metallicity enhancements or depletions depending on the specific accretion history (Helled and Bodenheimer 2010), but the relative elemental abundances (e.g. C, O, N, etc.) may be different depending on the formation location corresponding to the respective snow lines.

The existence of hot Jupiters in very close-in orbits (e.g. ≲0.1 AU) presents a key challenge to theories of giant planet formation. Neither GI nor CA discussed above is thought to operate in such a way that allows hot Jupiters to form in situ at their current locations close to the host stars. The disk cannot fragment at those distances (Gammie 2001; Rafikov 2005), and cores with sufficient mass to attract significant envelopes cannot form. Therefore, the existence of hot Jupiters requires some form of “migration” from their original formation locations to their present orbits. Two competing hypotheses suggest that the planets migrated either through interaction with the protoplanetary disk during their formation (Papaloizou et al. 2007), or by ‘disk-free’ mechanisms such as gravitational interactions with a third body (e.g. Rasio and Ford 1996; Fabrycky and Tremaine 2007). Measurements of spit-orbit misalignment (or stellar obliquity) for a large sample of hot Jupiters over the past decade (e.g. Triaud et al. 2010; Albrecht et al. 2012) have been advocated as possible metrics to distinguish between the two hypotheses for migration. In a simplistic view, migration through the disk was thought to likely align the orbital angular momentum vector of the planet with the stellar spin axis whereas disk-free migration via dynamical encounters could lead to spin-orbit misalignment. A significant number of large spin-orbit misalignments (or stellar obliquity) observed in hot Jupiter systems initially supported disk-free migration mechanisms (Winn et al. 2010). However, recent studies show that spin-orbit misalignments may also be caused by planet migration through disks that are themselves misaligned and due to star-disk interactions (Crida and Batygin 2014; Lai 2014). Consequently, observed dynamical properties of hot Jupiters have been unable to conclusively constrain their migration pathways.

**Fig. 12:**
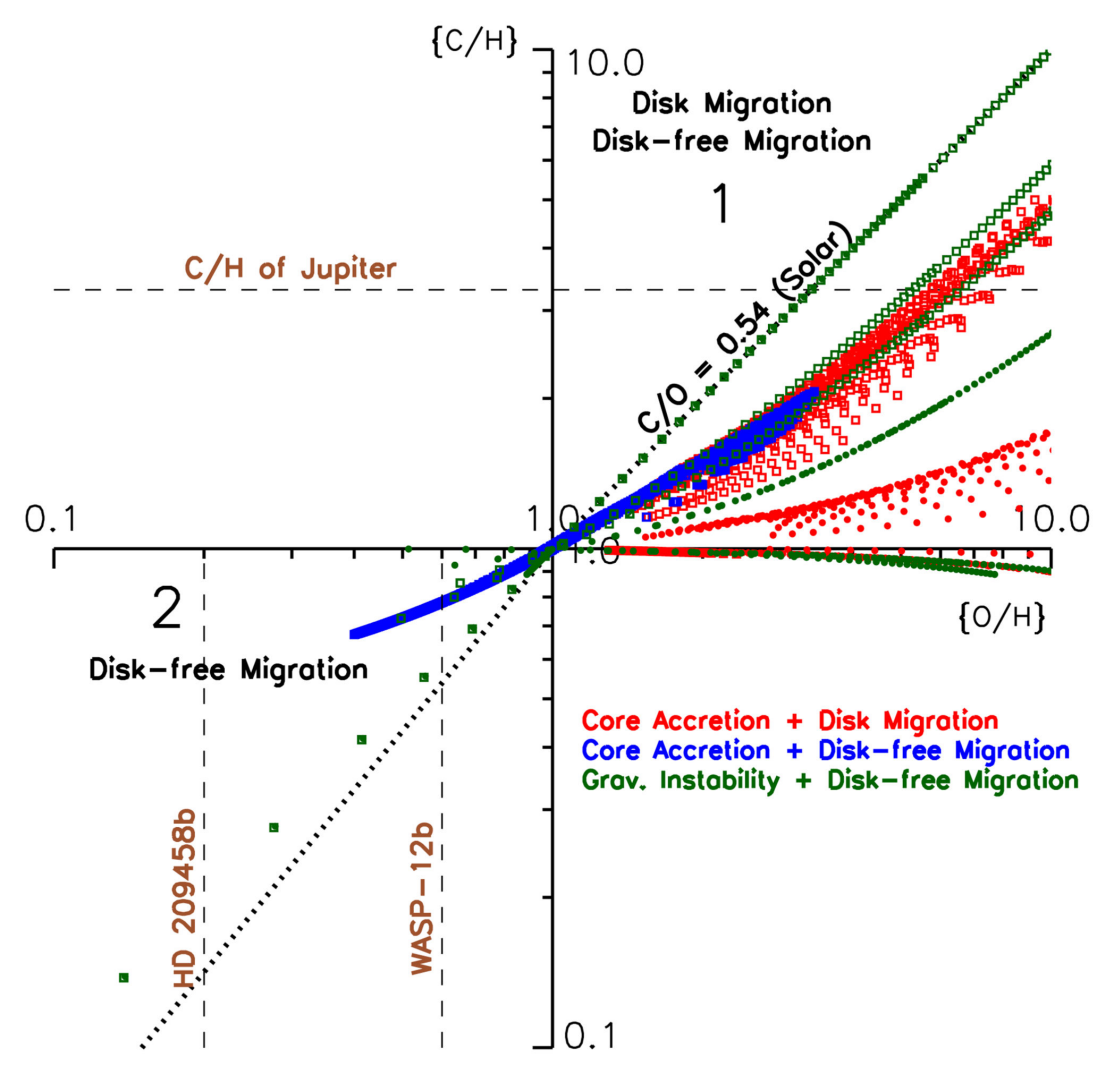
Predicted elemental O and C abundances in hot Jupiters from semi-analytic chemical models of hot Jupiter formation and migration (from Madhusudhan et al. 2014a). The *axes* show O/H and C/H abundances relative to solar values, and the *colored symbols* represent models with different formation-migration histories. *Each dot* represents a different model realization and shows the final O/H and C/H of the planet resulting from that model. The final elemental abundances in a planet depend critically on the amount of gas and solids accreted by the planet as well the region from which that material is accreted relative to the H_2_O, CO_2_, and CO *snow lines*, giving rise to the distinct loci of models in the O-C plane as shown. The results suggest that different migration mechanisms can cause different patterns of elemental enhancements in hot Jupiters

Instead, recent studies suggest that chemical abundances of hot Jupiters could provide stronger constraints on their formation and migration pathways. As discussed above, the O/H, C/H, and C/O ratios can change substantially with their formation locations in the protoplanetary disk. Madhusudhan et al. (2014a) suggested that atmospheric metallicities of hot Jupiter atmospheres could potentially constrain their migration mechanisms as migration through the disk is more likely to cause metal enrichment due to planetesimal accretion compared to disk-free migration mechanisms. The results lead to three key predictions as evident from Fig. 12. Firstly, planets migrating through the disk always accrete solids efficiently enough to result in solar or super-solar abundances (top-right quadrant in Fig. 12). Secondly, the C and O abundances are enhanced or depleted together, i.e. no cases in the top-left or bottom-right quadrants. Thirdly, planets with sub-stellar O and C abundances could not result from disk-migration. These findings imply that elemental abundances of hot Jupiters could potentially provide important constraints not only on their local formation environments but also on the migration pathways. Current observations are already providing the first constraints on the O and C abundances which in turn are being used to constrain formation and migration mechanisms (Madhusudhan et al. 2014a). These efforts will be further bolstered in the future as the spectral range of JWST instruments will contain strong molecular features of several molecules which will allow precise abundance estimates for a wider range of elements (e.g. O, C, N, P, S, Si, etc.).

## 8 Theory: Climates of Habitable Exoplanets

### 8.1 Habitability of Exoplanets

Existence of life requires many kinds of conditions. One of the critically necessary conditions for a planet to harbor life is permanent existence of liquid water on the planet’s surface. To maintain liquid water, the planetary surface temperature has to be between the freezing point of water (0 °C) and the boiling point (100 °C), i.e., at 1 bar,

(1) $$0\;{}^{\circ}\text{C}<T_{s}<100{}^{\circ}\text{C}.$$

In reality, the upper limit of surface temperature has to be lower than the boiling point of water. Otherwise, the planet would fall into the runaway greenhouse state. We will return to this issue in the next section. To retain a fairly dense atmosphere and large amount of water against escape processes, the planetary mass has to be large enough so that the planet has sufficiently strong gravity. However, the planetary mass cannot be too large. Otherwise, it could have accreted a massive H_2_-He envelope whose greenhouse effect along with the high pressure of the envelope would warm the surface and prevent water from being liquid, or it could be a Neptune-like ice-giant planet (Selsis et al. 2007; Venturini et al. 2015). Specifically, a habitable planet has to be a solid planet, and its mass should be in an approximate range given by:

(2) $$0.5M_{⊕}<M<10M_{⊕},$$

where, *M*_⊕_ denotes Earth mass (Selsis et al. 2007).

Based on the above two conditions, an Earth Similarity Index (ESI) has been proposed (Schulze-Makuch et al. 2011) which attempts to quantify the similarity of a given property of an exoplanet to that of the Earth and ranges between 0.0 and 1.0. There are about 30 exoplanets that have ESI greater than 0.5. In other words, there are about 30 potentially habitable exoplanets among nearly 2000 discovered exoplanets. Gl 581c is the first exoplanet that had ever been thought to be habitable (Udry et al. 2007). However, radiative-convective model simulations indicated that Gl 581c is too hot to maintain liquid water (Selsis et al. 2007; Hu and Ding 2011). Especially, Hu and Ding (2011) showed that the climate of Gl 581c can readily fall into runaway greenhouse state even if the CO_2_ level is extremely low and cloud albedo is extremely high. Gl 581d had also been considered a habitable super-Earth (Udry et al. 2007; Selsis et al. 2007; Von Bloh et al. 2007), and it is probably the first exoplanet whose habitability has drawn intensive studies. Wordsworth et al. (2010), Hu and Ding (2011), and Von Paris et al. (2011) all showed that at least 7 bars of CO_2_ are required to maintain the surface temperature of Gl 581d above the freezing point of water. Kepler 186f, which has a size similar to Earth’s, was also suggested to be a habitable exoplanet. However, calculations indicate that the surface temperature of Kepler 186f would be much colder than that of Gl 581d for same CO_2_ concentration because Kepler 186f receives less stellar radiation than Gl 581d does. So far, none of these potentially habitable exoplanets has been confirmed to be habitable.

### 8.2 Habitable Zones

Condition (1) defines a circumstellar zone in which a terrestrial planet can hold permanent liquid water on its surface. Such a zone is the so-called Habitable Zone (HZ). The HZ was first proposed by Shapley (1953) as the region around a star in which liquid water could exist on a planet’s surface. The term ‘habitable zone’ was first introduced by Huang (1960), in the context of planetary habitability and extraterrestrial life. The HZ was more precisely defined in later works (Kasting et al. 1993; Kopparapu 2013), based on results from one-dimensional radiative-convective climate models. In these works, various factors that influence the width of the HZ, such as surface albedo, atmospheric compositions, and stellar radiation spectra, were considered. Especially, Kasting et al. (1993) extensively addressed how the inner and outer edges of HZ are constrained by the positive water-vapor feedback and by the saturation limit of the maximum CO_2_ concentration, respectively. One might expect that the inner edge of HZ is at the distance where planetary surface temperature is equal the boiling point of water (100 °C). However, as shown by Ingersoll (1969), all liquid water on the planetary surface would completely evaporate into the atmosphere due to the positive water-vapor feedback once the surface temperature reaches about 70 °C, and the planet falls into the runaway greenhouse climate state. Then, water is lost throughout photolysis and hydrogen escape. The outer edge is constrained by the limit of CO_2_ saturation, with which CO_2_ condensation begins to take place. Thus, further increasing CO_2_ does not enhance the greenhouse effect. Instead, latent heat release by CO_2_ condensation even causes weakened greenhouse effect due to the decrease of lapse rate. Using the one-dimensional climate model, Kasting et al. (1993) found that conservative estimates of the inner and outer edges of HZ in our own Solar System is about 0.95 and 1.37 AU, respectively. Thus, the width of the solar HZ is about 0.42 AU. It is wider than the mean spacing between the four terrestrial planets in the Solar System, which is about 0.35 AU. It suggests that, statistically speaking, at least one of the terrestrial planets ought to be in the solar HZ. Kasting (2010) pointed out that the possibility that at least one rocky planet will be in the HZ should be high if such a statistical result can be applied to other exoplanetary systems. For darker stars like M dwarfs that have effective temperatures of about 3500 K, the HZ is much closer to the stars, at about 0.1 AU.

One uncertainty of the HZ width calculated from radiative-convective models is cloud radiative effects that are not included in these one-dimensional models. It was thought that the inner edge could be pushed closer to stars as the negative feedback of cloud reflection of stellar radiation is considered (Kasting et al. 1993; Selsis et al. 2007). Three-dimensional general circulation models (GCMs) include cloud radiative effects because GCMs have self-adjusted dynamical and physical processes of cloud formation. Several recent works have studied the HZ width with GCMs. However, Leconte et al. (2013) demonstrated that the inner edge of the solar HZ calculated from their GCM is nearly the same as that predicted by one-dimensional models. The reason why the inner edge does not move closer to Sun in their GCM is because of two important factors. One is that water-vapor absorption of solar radiation is higher in their model than previously assumed. The other one is that for high surface temperatures and water-rich atmosphere conditions high cirrus clouds increase in fraction faster than low stratus clouds. The former consists of ice particles and have greenhouse effects, and the later mainly reflects solar radiation and cools the surface. On the other hand, Yang et al. (2013) used a GCM to show that deep convective clouds develop around the substellar point for slow-rotating or tidally-locked exoplanets. These clouds largely reflect stellar radiation and cool the surface. As a result, the inner edge of the HZ is pushed much closer to the star, and the HZ width is nearly doubled.

It is worthwhile to point out that the cloud radiative effect on the inner edge of the HZ has large uncertainties. Parameterized clouds in GCMs are the primary source of uncertainty even in simulating modern climates where we have a wealth of direct observations. Thus, it is not clear to what extent cloud parameterizations in Earth GCMs can be applied to studying exoplanetary atmospheres. Using Earth GCMs to study the outer edge of the HZ also has great challenges. There is also lack of parameterizations of CO_2_ clouds. Radiative effects of CO_2_ clouds are not well understood (Forget and Pierrehumbert 1997).

### 8.3 Climates and Habitability of Tidal-Locking Exoplanets in the HZ of M Dwarfs

M dwarfs are the most common stars in the Universe (Rodono 1986). Thus, the probability of finding habitable exoplanets around M dwarfs is much higher than around other types of stars. Moreover, it is much easier to find habitable exoplanets closer to stars than that with greater distances with current observation techniques. Therefore, it is very likely to first discover habitable exoplanets around M dwarfs. Since M dwarfs have much weaker luminosities than Sun-like stars, the HZ around an M dwarf is typically about 0.1 AU, much closer than that for a solar-type star. However, the short distance of the HZ could also cause exoplanets in the HZ around M dwarfs to be uninhabitable, as discussed below.

First, exoplanets within such short distance to M dwarfs can be exposed to high levels of X-ray and extreme ultraviolet radiation (X-EUV) and strong particle fluxes from stellar winds or coronal mass ejections (Lammer et al. 2011). Such strong emissions are due to M dwarfs magnetic activity. It can generate significant atmospheric escapes to space and can even potentially cause atmosphere erosion or strip the whole atmosphere. For solar-type stars, such strong X-EUV emissions may be limited to the first few hundred millions years. However, the extreme irradiation could last several Gyr for M-type stars. Thus, exoplanets in the HZ of M-type stars may receive X-EUV fluxes that are 10–100 times higher than those in the HZ of solar-type stars of the same age (Selsis et al. 2007). X-EUV flux could cause a CO_2_ dominant atmosphere with modest amount of free oxygen (O_2_). Rapid escape of carbon from a CO_2_ dominant atmosphere could lead to the formation of O_2_ even without the presence of biological O_2_ production (Tian 2009). Photochemical models also suggest that O_3_ and CH_4_ can also be produced abiotically for different stellar characteristics and levels of volcanic outgassing (Domagal-Goldman et al. 2014). On the other hand, several other compounds, e.g. of sulfur, could act as potential biosignatures (Domagal-Goldman et al. 2011). At the current stage, more observational and quantitative modeling works are needed for understanding the effects of the extreme irradiation on atmospheres of exoplanets in the HZ around M dwarfs (see e.g. Segura et al. 2010).

Second, habitable exoplanets orbiting M-type stars are very likely to be tidally locked to their primaries due to strong forcing of gravitational gradients. Tidal-locking exoplanets receive very uneven stellar heating because their one side permanently faces their stars and the other side remains dark. While the dayside can be warm enough to sustain liquid water, the nightside could be so cold that any gases condense out there. If there exists atmosphere collapse, exoplanets in the HZ of M dwarfs would be uninhabitable. There were works that studied whether atmospheric heat transports could prevent atmosphere from collapse on the nightside using simplified or sophisticate models (Haberle et al. 1996; Joshi et al. 1997; Joshi 2003; Pierrehumbert 2011). They all showed that for a sufficiently dense atmosphere (surface air pressure greater than 0.1 bar) atmospheric heat transports would be able to warm the nightside and maintain nightside surface temperature above the condensation point of CO_2_ (−78.5 °C at 1 bar of CO_2_ partial pressure, and much lower at lower partial pressures). Therefore, atmosphere collapse can hardly happen as long as exoplanets are able to attract a fairly dense atmosphere envelope. However, more recent works suggest that the threshold of air pressure to prevent atmosphere collapse requires further investigation (Heng and Kopparla 2012; Wordsworth 2015).

**Fig. 13:**
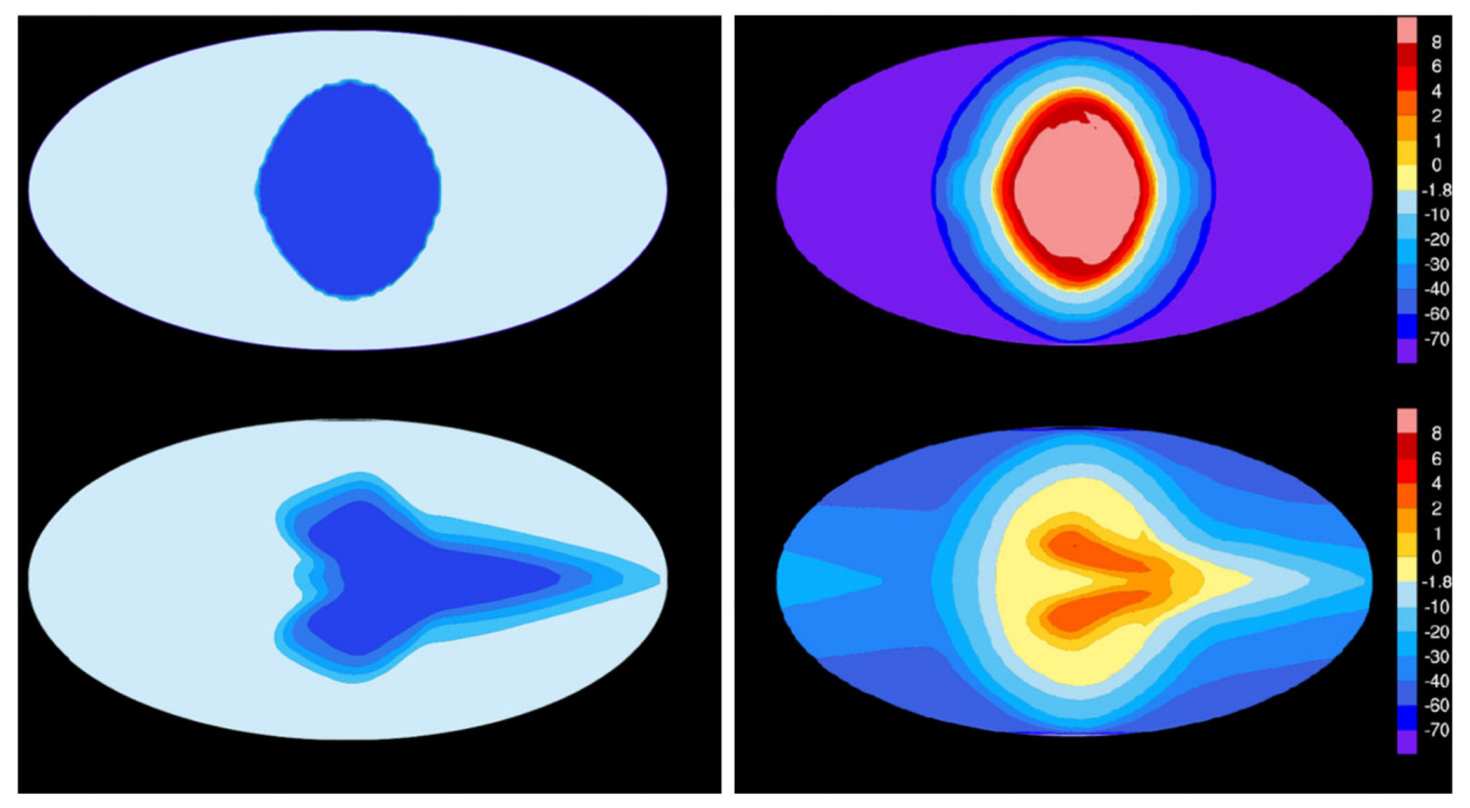
Spatial patterns of open-ocean areas and surface temperatures. *Left panels*: sea-ice fraction. *Blue colors* indicate open-ocean areas, and *light-gray* indicates ice coverage. *Right panels*: surface temperatures, *color bar* unit is °C. *Top panels*: with only atmospheric heat transport, and *bottom panels*: heat transports by both atmosphere and ocean. The substellar point is *at the center of each panel*. After Hu and Yang Hu and Yang (2014)

The above studies have not taken ocean into account. If an exoplanet has an extensive ocean, its habitability also involves ocean heat transports and the positive sea-ice albedo feedback (Hu and Yang 2014). On the one hand, it is well known that ocean heat transports are equally important in Earth’s climate (Peixoto and Oort 1992). In the presence of sea ice, ocean heat transports are likely to be especially important, since it is known from studies of the Snowball Earth phenomenon in Earth-like conditions that ocean heat transports are very effective in holding back the advance of the sea-ice margin (Pierrehumbert 2011; Yang et al. 2012a,b,c). On the other hand, in the presence of sea ice there exists the possibility that the exoplanet could be locked in a globally glaciated Snowball state due to the positive ice-albedo feedback. Note that the distribution of ice on tidally locked exoplanets is a particularly interesting issue only for M-type stars, since exoplanets orbiting hotter stars in orbits close enough to yield tidal-locking are likely to be too hot to permit ice, and may even be too hot to retain liquid water. Using a fully coupled atmospheric-oceanic GCM (AOGCM), Hu and Yang (2014) carried out simulations and demonstrated that ocean heat transports substantially extend the area of open water along the equator, and that the open-ocean area shows a lobster-like spatial pattern (Fig. 13), instead of an eyeball as shown in Pierrehumbert (2011). Figure 13 also shows that as CO_2_ concentration increases, the dayside temperature does not increase very much, but the nightside temperature increases a lot, indicating that most heat is transported from the dayside to the nightside by ocean currents. They also showed that ocean heat transports can even lead to complete deglaciation of the nightside as greenhouse gas concentration is sufficiently high or stellar radiation is sufficiently strong. By contrast, the open-ocean area is not expanded very much by atmospheric heat transports alone for the same high level of CO_2_. They also showed that it is more ready for aqua-planets to fall into runaway greenhouse and runaway freezing at the inner and outer edges, respectively, compared with simulation results without a dynamic ocean. It implies that the HZ would be narrower for aqua-planets or exoplanets with extensive oceans. These results indicate that ocean heat transports play critically important roles in determining the climate state and habitability of exoplanets.

**Fig. 14:**
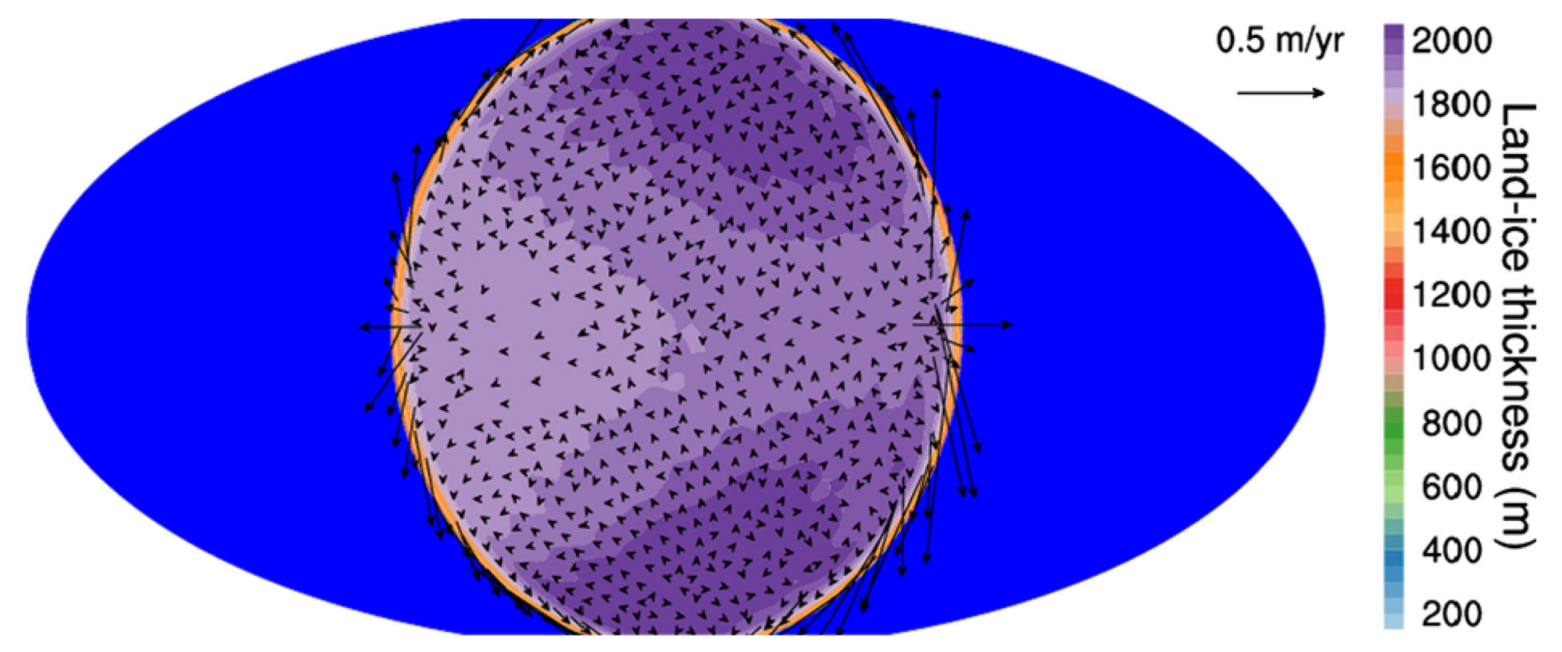
Ice sheets over a supercontinent spanning the entire nightside. *Color shading* indicates ice-sheet thickness, and *arrows* indicate ice-sheet flow velocity. Geothermal flux is 0.1 W m^−2^ in the simulation. Adapted from Yang et al. (2014)

The third problem is whether water could be completely trapped on the nightside of tidal-locking exoplanets in the HZ of M dwarfs (Menou 2013). Although Fig. 13 shows that the lowest surface temperature on the nightside is about −40 °C, well above the condensation point of CO_2_, it is still far below the freezing point of water. Therefore, there is the possibility of water trap on the nightside. Hu and Yang (2014) showed that as long as the ocean is not completely frozen, wind stresses transport sea ice toward the dayside and the ocean carries heat toward the nightside. As a result, sea-ice thickness on the nightside remains thin, less than 10 meters. Yang et al. (2014) studied that a case with a super-continent on the nightside and an ocean on the dayside. In this case, ocean heat transports from the dayside to the nightside are ceased. It is found that ice sheets over the nightide supercontinent can grow 2000 m thick if geothermal heat flux is close to Earth’s or smaller (Fig. 14). It suggests that if the dayside ocean is deeper than 2000 m, water will not be completely trapped on the nightside continent. Only exoplanets with a geothermal heat flux lower than Earths, and much of their surface covered by continents would be susceptible to complete water trapping.

### 8.4 Observable Signatures of Tidally-Locked Habitable Exoplanets

The above climate patterns have important implications for future observations. Showman et al. (2009), Showman and Polvani (2011), Heng and Showman (2015) showed that an equatorial superrotating jet stream develops in the atmosphere of tidal-locking exoplanets. The hot spot of tidal-locking exoplanets is advected to the downstream of the substellar point by the equatorial superrotating jet stream. Their simulation results are confirmed by observations for hot Jupiters (Knutson et al. 2009). It was shown that the hot spot is shifted 20°−30° east of the substellar point of hot Jupiter HD 189733b. The equatorial superrotating flow is a common feature for tidal-locking exoplanets (Heng and Vogt 2011; Hu and Ding 2013). Thus, the hot-spot shift shall also be observed from the thermal phase curve of terrestrial exoplanets. In fact, the climate pattern in Fig. 13 also suggests eastward shift of the hot spot. Especially, as a dynamic ocean is considered, the equatorial ocean current, together with the equatorial atmospheric jet stream, can cause further downstream shift of the hot spot. Cowan et al. (2012b) studied how surface thermal inertia of Earth-like exoplanets leads to distinguishable thermal phase curve for observations. They studied two types of climate states. One is the temperate climate which is just like the modern Earth with low- and middle-latitude oceans and polar ice-caps. The other one is the Snowball state. They found that the former has a relatively flat thermal phase curve because its relatively large surface thermal inertia tends to damp out the amplitude of the thermal phase curve. Wang et al. (2014) argued that terrestrial exoplanets around M dwarfs could have different climate states. They use an atmospheric GCM coupled with a slab ocean to show that exoplanets with nonzero eccentricities could have different spin-orbit resonance states different from the synchronous rotation state, and these exoplanets should have a striped-ball climate pattern, with a global belt of open ocean at low and middle latitudes and ice caps over both polar regions. This is in contrast to synchronous rotating habitable exoplanets around M dwarfs that have an eyeball climate pattern a limited region of open water on the dayside and ice on the rest of the planet (Pierrehumbert 2011; Hu and Yang 2014). They pointed out that the striped-ball climate state should be common for habitable exoplanets in large eccentric orbits around M dwarfs. They further suggested that these different climate patterns can be observed by future exoplanet detection missions. Overall, the features of climate patterns provide future observations of habitable exoplanets with additional constraints and information.

## 9 Future Prospects

Major observational advancements are happening in two key directions: (a) detections of exoplanets around bright stars, and (b) high precision observations of exoplanetary spectra. Firstly, bright exoplanet host stars are important to obtain precise measurements of exoplanetary masses, radii, and atmospheric spectra. Upcoming space missions, CHEOPS (Broeg et al. 2014) and TESS (Ricker et al. 2015), as well as various ground-based surveys (e.g. NGTS, SPECULOOS, MEARTH, etc.) are expected to find thousands of transiting exoplanets, including hundreds of super-Earths, orbiting nearby bright stars within the next five years (also see chapter on future landscape by Fridlund et al. 2016, this issue). During the same time, major ground-based direct imaging platforms (e.g. SPHERE, GPI, etc.) are also expected to find at least dozens of exoplanets at wide orbital separations orbiting nearby young stars. On a longer run, early 2020s, the recently selected ESA mission, PLATO (Rauer et al. 2014), will discover numerous transiting exoplanets in habitable zones of nearby stars. Secondly, major parallel efforts are also being pursued for spectroscopic observations of exoplanetary atmospheres. In this regard, enormous amount of time is currently being dedicated on the Hubble and Spitzer space telescopes, as well as major ground-based telescopes (e.g. VLT, Keck, Gemini, Magellan, CFHT, etc.). In addition, the James Webb Space Telescope (*JWST*) scheduled for launch in 2018 will revolutionize exoplanetary spectroscopy. By early 2020s, next generation ground-based telescopes, such as the European-Extremely Large Telescope (E-ELT), will further revolutionize the field. Future spectra with *JWST* would be of unprecedented precision and resolution which will enable us to derive precise chemical abundances for transiting exoplanets. In addition, JWST would also be able to observe atmospheres of much cooler low-mass planets that are possible to observe today.

## References

[R1] Abe Y, Matsui T (1985). The formation of an impact-generated H_2_O atmosphere and its implications for the early thermal history of the Earth. J Geophys Res.

[R2] Ackerman AS, Marley MS (2001). Precipitating condensation clouds in substellar atmospheres. Astrophys J.

[R3] Agúndez M, Venot O, Iro N, Selsis F, Hersant F, Hébrard E, Dobrijevic M (2012). The impact of atmospheric circulation on the chemistry of the hot Jupiter HD 209458b. Astron Astrophys.

[R4] Agúndez M, Parmentier V, Venot O, Hersant F, Selsis F (2014a). Pseudo 2D chemical model of hot-Jupiter atmospheres: Application to HD 209458b and HD 189733b. Astron Astrophys.

[R5] Agúndez M, Venot O, Selsis F, Iro N (2014b). The puzzling chemical composition of GJ 436b’s atmosphere: Influence of tidal heating on the chemistry. Astrophys J.

[R6] Albrecht S, Winn JN, Johnson JA, Howard AW, Marcy GW, Butler RP, Arriagada P, Crane JD, Shectman SA, Thompson IB, Hirano T (2012). Obliquities of hot Jupiter host stars: Evidence for tidal interactions and primordial misalignments. Astrophys J.

[R7] Ali-Dib M, Mousis O, Petit J-M, Lunine JI (2014). Carbon-rich planet formation in a solar composition disk. Astrophys J.

[R8] Alibert Y, Mordasini C, Benz W, Winisdoerffer C (2005). Models of giant planet formation with migration and disc evolution. Astron Astrophys.

[R9] Alibert Y, Baraffe I, Benz W, Chabrier G, Mordasini C, Lovis C, Mayor M, Pepe F, Bouchy F, Queloz D, Udry S (2006). Formation and structure of the three Neptune-mass planets system around HD 69830. Astron Astrophys.

[R10] Allard F, Hauschildt PH, Baraffe I, Chabrier G (1996). Synthetic spectra and mass determination of the brown dwarf Gl 229B. Astrophys J Lett.

[R11] Allard F, Hauschildt PH, Alexander DR, Starrfield S (1997). Model atmospheres of very low mass stars and brown dwarfs. Annu Rev Astron Astrophys.

[R12] Asplund M, Grevesse N, Sauval AJ, Scott P (2009). The chemical composition of the Sun. Annu Rev Astron Astrophys.

[R13] Astudillo-Defru N, Rojo P (2013). Ground-based detection of calcium and possibly scandium and hydrogen in the atmosphere of HD 209458b. Astron Astrophys.

[R14] Atreya SK (1986). Atmospheres and Ionospheres of the Outer Planets and Their Satellites.

[R15] Atreya SK, Wong A-S (2005). Coupled clouds and chemistry of the giant planets—A case for multiprobes. Space Sci Rev.

[R16] Bai (2016). Space Sci Rev.

[R17] Bailey RL, Helling C, Hodosán G, Bilger C, Stark CR (2014). Ionization in atmospheres of brown dwarfs and extrasolar planets VI: Properties of large-scale discharge events. Astrophys J.

[R18] Baraffe I, Chabrier G, Barman T (2010). The physical properties of extra-solar planets. Rep Prog Phys.

[R19] Barman T (2007). Identification of absorption features in an extrasolar planet atmosphere. Astrophys J Lett.

[R20] Barman TS, Hauschildt PH, Allard F (2005). Phase-dependent properties of extrasolar planet atmospheres. Astrophys J.

[R21] Barman TS, Macintosh B, Konopacky QM, Marois C (2011). Clouds and chemistry in the atmosphere of extrasolar planet HR8799b. Astrophys J.

[R22] Barman TS, Konopacky QM, Macintosh B, Marois C (2015). Simultaneous detection of water, methane, and carbon monoxide in the atmosphere of exoplanet HR8799b. Astrophys J.

[R23] Barstow JK, Aigrain S, Irwin PGJ, Hackler T, Fletcher LN, Lee JM, Gibson NP (2014). Clouds on the hot Jupiter HD189733b: Constraints from the reflection spectrum. Astrophys J.

[R24] Batalha NM, Rowe JF, Bryson ST, Barclay T, Burke CJ, Caldwell DA, Christiansen JL, Mullally F, Thompson SE, Brown TM, Dupree AK (2013). Planetary candidates observed by Kepler. III. Analysis of the first 16 months of data. Astrophys J Suppl Ser.

[R25] Bean JL, Désert J-M, Kabath P, Stalder B, Seager S, Miller-Ricci Kempton E, Berta ZK, Homeier D, Walsh S, Seifahrt A (2011). The optical and near-infrared transmission spectrum of the super-Earth GJ 1214b: Further evidence for a metal-rich atmosphere. Astrophys J.

[R26] Beaulieu JP, Carey S, Ribas I, Tinetti G (2008). Primary transit of the planet HD 189733b at 3.6 and 5.8 μm. Astrophys J.

[R27] Beaulieu J-P, Tinetti G, Kipping DM, Ribas I, Barber RJ, Cho JY-K, Polichtchouk I, Tennyson J, Yurchenko SN, Griffith CA, Batista V (2011). Methane in the atmosphere of the transiting hot Neptune GJ436B?. Astrophys J.

[R28] Benneke B (2015). Strict upper limits on the carbon-to-oxygen ratios of eight hot Jupiters from self-consistent atmospheric retrieval. ArXiv e-prints.

[R29] Benneke B, Seager S (2013). How to distinguish between cloudy mini-Neptunes and water/volatile-dominated super-Earths. Astrophys J.

[R30] Benz W, Ida S, Alibert Y, Lin D, Mordasini C, Beuther H, Klessen RS, Dullemond CP, Henning T (2014). Planet population synthesis. Protostars and Planets VI.

[R31] Berta ZK, Charbonneau D, Désert J-M, Miller-Ricci Kempton E, McCullough PR, Burke CJ, Fortney JJ, Irwin J, Nutzman P, Homeier D (2012). The flat transmission spectrum of the super-Earth GJ1214b from wide field camera 3 on the Hubble Space Telescope. Astrophys J.

[R32] Bilger C, Rimmer P, Helling C (2013). Small hydrocarbon molecules in cloud-forming brown dwarf and giant gas planet atmospheres. Mon Not R Astron Soc.

[R33] Birkby JL, de Kok RJ, Brogi M, de Mooij EJW, Schwarz H, Albrecht S, Snellen IAG (2013). Detection of water absorption in the day side atmosphere of HD 189733 b using ground-based high-resolution spectroscopy at 3.2 μm. Mon Not R Astron Soc.

[R34] Blum J, Wurm G (2008). The growth mechanisms of macroscopic bodies in protoplanetary disks. Annu Rev Astron Astrophys.

[R35] Borucki WJ, Koch DG, Basri G, Batalha N, Brown TM, Bryson ST, Caldwell D, Christensen-Dalsgaard J, Cochran WD, DeVore E, Dunham EW (2011). Characteristics of planetary candidates observed by Kepler. II. Analysis of the first four months of data. Astrophys J.

[R36] Boss AP (1997). Giant planet formation by gravitational instability. Science.

[R37] Boss AP (2000). Possible rapid gas giant planet formation in the solar nebula and other protoplanetary disks. Astrophys J Lett.

[R38] Bourrier V, Lecavelier des Etangs A, Dupuy H, Ehrenreich D, Vidal-Madjar A, Hébrard G, Ballester GE, Désert J-M, Ferlet R, Sing DK, Wheatley PJ (2013). Atmospheric escape from HD 189733b observed in H I Lyman-*α*: Detailed analysis of HST/STIS September 2011 observations. Astron Astrophys.

[R39] Bowler BP, Liu MC, Dupuy TJ, Cushing MC (2010). Near-infrared spectroscopy of the extrasolar planet HR 8799 b. Astrophys J.

[R40] Brasseur GP, Orlando JJ, Tyndall GS (1999). Atmospheric Chemistry and Global Change.

[R41] Broeg C, Benz W, Thomas N, Cheops Team (2014). The CHEOPS mission. Contrib Astron Obs Skaln Pleso.

[R42] Brogi M, Snellen IAG, de Kok RJ, Albrecht S, Birkby J, de Mooij EJW (2012). The signature of orbital motion from the dayside of the planet τ Boötis b. Nature.

[R43] Brogi M, Snellen IAG, de Kok RJ, Albrecht S, Birkby JL, de Mooij EJW (2013). Detection of molecular absorption in the dayside of exoplanet 51 Pegasi b?. Astrophys J.

[R44] Brogi M, de Kok RJ, Birkby JL, Schwarz H, Snellen IAG (2014). Carbon monoxide and water vapor in the atmosphere of the non-transiting exoplanet HD 179949 b. Astron Astrophys.

[R45] Brown TM (2001). Transmission spectra as diagnostics of extrasolar giant planet atmospheres. Astrophys J.

[R46] Burke CJ, Bryson ST, Mullally F, Rowe JF, Christiansen JL, Thompson SE, Coughlin JL, Haas MR, Batalha NM, Caldwell DA, Jenkins JM (2014). Planetary candidates observed by Kepler IV: Planet sample from q1–q8 (22 months). Astrophys J Suppl Ser.

[R47] Burrows A, Sharp CM (1999). Chemical equilibrium abundances in brown dwarf and extrasolar giant planet atmospheres. Astrophys J.

[R48] Burrows A, Marley M, Hubbard WB, Lunine JI, Guillot T, Saumon D, Freedman R, Sudarsky D, Sharp C (1997). A nongray theory of extrasolar giant planets and brown dwarfs. Astrophys J.

[R49] Burrows A, Hubbard WB, Lunine JI, Liebert J (2001). The theory of brown dwarfs and extrasolar giant planets. Rev Mod Phys.

[R50] Burrows A, Sudarsky D, Lunine JI (2003). Beyond the T dwarfs: Theoretical spectra, colors, and detectability of the coolest brown dwarfs. Astrophys J.

[R51] Burrows A, Sudarsky D, Hubeny I (2006). Theory for the secondary eclipse fluxes, spectra, atmospheres, and light curves of transiting extrasolar giant planets. Astrophys J.

[R52] Burrows A, Budaj J, Hubeny I (2008). Theoretical spectra and light curves of close-in extrasolar giant planets and comparison with data. Astrophys J.

[R53] Burton JR, Watson CA, Rodríguez-Gil P, Skillen I, Littlefair SP, Dhillon S, Pollacco D (2015). Defocused transmission spectroscopy: A potential detection of sodium in the atmosphere of WASP-12b. Mon Not R Astron Soc.

[R54] Castan T, Menou K (2011). Atmospheres of hot super-Earths. Astrophys J Lett.

[R55] Charbonneau D, Brown TM, Noyes RW, Gilliland RL (2002). Detection of an extrasolar planet atmosphere. Astrophys J.

[R56] Chassefière E (1996). Hydrodynamic escape of oxygen from primitive atmospheres: Applications to the cases of Venus and Mars. Icarus.

[R57] Chatterjee S, Tan JC (2014). Inside-out planet formation. Astrophys J.

[R58] Chiang E, Laughlin G (2013). The minimum-mass extrasolar nebula: In situ formation of close-in super-Earths. Mon Not R Astron Soc.

[R59] Chiang E, Youdin AN (2010). Forming planetesimals in solar and extrasolar nebulae. Annu Rev Earth Planet Sci.

[R60] Chilcote J, Barman T, Fitzgerald MP, Graham JR, Larkin JE, Macintosh B, Bauman B, Burrows AS, Cardwell A, De Rosa RJ, Dillon D (2015). The first H-band spectrum of the giant planet *β* Pictoris b. Astrophys J Lett.

[R61] Ciesla FJ, Cuzzi JN (2006). The evolution of the water distribution in a viscous protoplanetary disk. Icarus.

[R62] Colón KD, Ford EB, Redfield S, Fortney JJ, Shabram M, Deeg HJ, Mahadevan S (2012). Probing potassium in the atmosphere of HD 80606b with tunable filter transit spectrophotometry from the Gran Telescopio Canarias. Mon Not R Astron Soc.

[R63] Cooper CS, Showman AP (2006). Dynamics and disequilibrium carbon chemistry in hot Jupiter atmospheres, with application to HD 209458b. Astrophys J.

[R64] Cowan NB, Machalek P, Croll B, Shekhtman LM, Burrows A, Deming D, Greene T, Hora JL (2012a). Thermal phase variations of WASP-12b: Defying predictions. Astrophys J.

[R65] Cowan NB, Voigt A, Abbot DS (2012b). Thermal phases of Earth-like planets: Estimating thermal inertia from eccentricity, obliquity, and diurnal forcing. Astrophys J.

[R66] Crida A, Batygin K (2014). Spin-orbit angle distribution and the origin of (mis)aligned hot Jupiters. Astron Astrophys.

[R67] Croll B, Albert L, Lafreniere D, Jayawardhana R, Fortney JJ (2010). Near-infrared thermal emission from the hot Jupiter TrES-2b: Ground-based detection of the secondary eclipse. Astrophys J.

[R68] Crossfield IJM, Barman T, Hansen BMS, Tanaka I, Kodama T (2012). Re-evaluating WASP-12b: Strong emission at 2.315 μm, deeper occultations, and an isothermal atmosphere. Astrophys J.

[R69] Crouzet N, McCullough PR, Deming D, Madhusudhan N (2014). Water vapor in the spectrum of the extrasolar planet HD 189733b. II. The eclipse. Astrophys J.

[R70] Currie T, Burrows A, Itoh Y, Matsumura S, Fukagawa M, Apai D, Madhusudhan N, Hinz PM, Rodigas TJ, Kasper M, Pyo T-S (2011). A combined Subaru/VLT/MMT 1-5 μm study of planets orbiting HR 8799: Implications for atmospheric properties, masses, and formation. Astrophys J.

[R71] D’Angelo G, Durisen RH, Lissauer JJ, Seager S (2010). Giant planet formation. Exoplanets.

[R72] de Kok RJ, Brogi M, Snellen IAG, Birkby J, Albrecht S, de Mooij EJW (2013). Detection of carbon monoxide in the high-resolution day-side spectrum of the exoplanet HD 189733b. Astron Astrophys.

[R73] Deming D, Wilkins A, McCullough P, Burrows A, Fortney JJ, Agol E, Dobbs-Dixon I, Madhusudhan N, Crouzet N, Desert J-M, Gilliland RL (2013). Infrared transmission spectroscopy of the exoplanets HD 209458b and XO-1b using the wide field camera-3 on the Hubble Space Telescope. Astrophys J.

[R74] Demory B-O, Seager S, Madhusudhan N, Kjeldsen H, Christensen-Dalsgaard J, Gillon M, Rowe JF, Welsh WF, Adams ER, Dupree A, McCarthy D (2011). The high albedo of the hot Jupiter Kepler-7 b. Astrophys J Lett.

[R75] Demory B-O, de Wit J, Lewis N, Fortney J, Zsom A, Seager S, Knutson H, Heng K, Madhusudhan N, Gillon M, Barclay T (2013). Inference of inhomogeneous clouds in an exoplanet atmosphere. Astrophys J Lett.

[R76] Désert J-M, Vidal-Madjar A, Lecavelier Des Etangs A, Sing D, Ehrenreich D, Hébrard G, Ferlet R (2008). TiO and VO broad band absorption features in the optical spectrum of the atmosphere of the hot-Jupiter <ASTROBJ>HD 209458b</ASTROBJ>. Astron Astrophys.

[R77] Désert J-M, Lecavelier des Etangs A, Hébrard G, Sing DK, Ehrenreich D, Ferlet R, Vidal-Madjar A (2009). Search for carbon monoxide in the atmosphere of the transiting exoplanet HD 189733b. Astrophys J.

[R78] Désert J-M, Bean J, Miller-Ricci Kempton E, Berta ZK, Charbonneau D, Irwin J, Fortney J, Burke CJ, Nutzman P (2011). Observational evidence for a metal-rich atmosphere on the super-Earth GJ1214b. Astrophys J Lett.

[R79] Diamond-Lowe H, Stevenson KB, Bean JL, Line MR, Fortney JJ (2014). New analysis indicates no thermal inversion in the atmosphere of HD 209458b. Astrophys J.

[R80] Dodson-Robinson SE, Bodenheimer P (2010). The formation of Uranus and Neptune in solid-rich feeding zones: Connecting chemistry and dynamics. Icarus.

[R81] Dodson-Robinson SE, Willacy K, Bodenheimer P, Turner NJ, Beichman CA (2009). Ice lines, planetesimal composition and solid surface density in the solar nebula. Icarus.

[R82] Domagal-Goldman SD, Meadows VS, Claire MW, Kasting JF (2011). Using biogenic sulfur gases as remotely detectable biosignatures on anoxic planets. Astrobiology.

[R83] Domagal-Goldman SD, Segura A, Claire MW, Robinson TD, Meadows VS (2014). Abiotic ozone and oxygen in atmospheres similar to prebiotic Earth. Astrophys J.

[R84] Ehrenreich D, Bonfils X, Lovis C, Delfosse X, Forveille T, Mayor M, Neves V, Santos NC, Udry S, Ségransan D (2014). Near-infrared transmission spectrum of the warm-Uranus GJ 3470b with the wide field camera-3 on the Hubble Space Telescope. Astron Astrophys.

[R85] Ehrenreich D, Bourrier V, Wheatley PJ, Lecavelier des Etangs A, Hébrard G, Udry S, Bonfils X, Delfosse X, Désert J-M, Sing DK, Vidal-Madjar A (2015). A giant comet-like cloud of hydrogen escaping the warm Neptune-mass exoplanet GJ 436b. Nature.

[R86] Elkins-Tanton LT, Seager S (2008). Ranges of atmospheric mass and composition of super-Earth exoplanets. Astrophys J.

[R87] Esteves LJ, De Mooij EJW, Jayawardhana R (2015). Changing phases of alien worlds: Probing atmospheres of Kepler planets with high-precision photometry. Astrophys J.

[R88] Evans TM, Pont F, Sing DK, Aigrain S, Barstow JK, Désert J-M, Gibson N, Heng K, Knutson HA, Lecavelier des Etangs A (2013). The deep blue color of HD 189733b: Albedo measurements with Hubble Space Telescope/space telescope imaging spectrograph at visible wavelengths. Astrophys J Lett.

[R89] Fabrycky D, Tremaine S (2007). Shrinking binary and planetary orbits by Kozai cycles with tidal friction. Astrophys J.

[R90] Fang (2016). Space Sci Rev.

[R91] Fegley B, Lodders K (1994). Chemical models of the deep atmospheres of Jupiter and Saturn. Icarus.

[R92] Fegley B, Lodders K (1996). Atmospheric chemistry of the brown dwarf Gliese 229B: Thermochemical equilibrium predictions. Astrophys J Lett.

[R93] Fischer DA, Howard AW, Laughlin GP, Macintosh B, Mahadevan S, Sahlmann J, Yee JC (2014). Exoplanet detection techniques. Protostars and Planets VI.

[R94] Forget F, Pierrehumbert RT (1997). Warming early Mars with carbon dioxide clouds that scatter infrared radiation. Science.

[R95] Forget F, Hourdin F, Fournier R, Hourdin C, Talagrand O, Collins M, Lewis SR, Read PL, Huot J-P (1999). Improved general circulation models of the Martian atmosphere from the surface to above 80 km. J Geophys Res.

[R96] Fortney JJ (2005). The effect of condensates on the characterization of transiting planet atmospheres with transmission spectroscopy. Mon Not R Astron Soc.

[R97] Fortney JJ, Nettelmann N (2010). The interior structure, composition, and evolution of giant planets. Space Sci Rev.

[R98] Fortney JJ, Sudarsky D, Hubeny I, Cooper CS, Hubbard WB, Burrows A, Lunine JI (2003). On the indirect detection of sodium in the atmosphere of the planetary companion to HD 209458. Astrophys J.

[R99] Fortney JJ, Marley MS, Lodders K, Saumon D, Freedman R (2005). Comparative planetary atmospheres: Models of TrES-1 and HD 209458b. Astrophys J Lett.

[R100] Fortney JJ, Lodders K, Marley MS, Freedman RS (2008a). A unified theory for the atmospheres of the hot and very hot Jupiters: Two classes of irradiated atmospheres. Astrophys J.

[R101] Fortney JJ, Marley MS, Saumon D, Lodders K (2008b). Synthetic spectra and colors of young giant planet atmospheres: Effects of initial conditions and atmospheric metallicity. Astrophys J.

[R102] Fortney JJ, Shabram M, Showman AP, Lian Y, Freedman RS, Marley MS, Lewis NK (2010). Transmission spectra of three-dimensional hot Jupiter model atmospheres. Astrophys J.

[R103] Fortney JJ, Mordasini C, Nettelmann N, Kempton EM-R, Greene TP, Zahnle K (2013). A framework for characterizing the atmospheres of low-mass low-density transiting planets. Astrophys J.

[R104] Fossati L, Haswell CA, Froning CS, Hebb L, Holmes S, Kolb U, Helling C, Carter A, Wheatley P, Collier Cameron A, Loeillet B (2010). Metals in the exosphere of the highly irradiated planet WASP-12b. Astrophys J Lett.

[R105] Fraine J, Deming D, Benneke B, Knutson H, Jordán A, Espinoza N, Madhusudhan N, Wilkins A, Todorov K (2014). Water vapour absorption in the clear atmosphere of a Neptune-sized exoplanet. Nature.

[R106] Fressin F, Torres G, Charbonneau D, Bryson ST, Christiansen J, Dressing CD, Jenkins JM, Walkowicz LM, Batalha NM (2013). The false positive rate of Kepler and the occurrence of planets. Astrophys J.

[R107] Freytag B, Allard F, Ludwig H-G, Homeier D, Steffen M (2010). The role of convection, overshoot, and gravity waves for the transport of dust in M dwarf and brown dwarf atmospheres. Astron Astrophys.

[R108] Fridlund (2016). Space Sci Rev.

[R109] Gammie CF (2001). Nonlinear outcome of gravitational instability in cooling, gaseous disks. Astrophys J.

[R110] García Muñoz A (2007). Physical and chemical aeronomy of HD 209458b. Planet Space Sci.

[R111] Gibson NP, Pont F, Aigrain S (2011). A new look at NICMOS transmission spectroscopy of HD 189733, GJ-436 and XO-1: No conclusive evidence for molecular features. Mon Not R Astron Soc.

[R112] Gibson NP, Aigrain S, Pont F, Sing DK, Désert J-M, Evans TM, Henry G, Husnoo N, Knutson H (2012). Probing the haze in the atmosphere of HD 189733b with Hubble Space Telescope/WFC3 transmission spectroscopy. Mon Not R Astron Soc.

[R113] Gibson NP, Aigrain S, Barstow JK, Evans TM, Fletcher LN, Irwin PGJ (2013). The optical transmission spectrum of the hot Jupiter HAT-P-32b: Clouds explain the absence of broad spectral features?. Mon Not R Astron Soc.

[R114] Gladstone GR, Allen M, Yung YL (1996). Hydrocarbon photochemistry in the upper atmosphere of Jupiter. Icarus.

[R115] Goldreich P, Lithwick Y, Sari R (2004). Planet formation by coagulation: A focus on Uranus and Neptune. Annu Rev Astron Astrophys.

[R116] Grillmair CJ, Burrows A, Charbonneau D, Armus L, Stauffer J, Meadows V, van Cleve J, von Braun K, Levine D (2008). Strong water absorption in the dayside emission spectrum of the planet HD189733b. Nature.

[R117] Guillot T (2005). The interiors of giant planets: Models and outstanding questions. Annu Rev Earth Planet Sci.

[R118] Guillot T (2010). On the radiative equilibrium of irradiated planetary atmospheres. Astron Astrophys.

[R119] Haberle RM, McKay CP, Tyler D, Reynolds RT (1996). Can synchronously rotating planets support an atmosphere?. Circumstellar Habitable Zones.

[R120] Hadden S, Lithwick Y (2014). Densities and eccentricities of 139 Kepler planets from transit time variations. Astrophys J.

[R121] Haghighipour N (2013). The formation and dynamics of super-Earth planets. Annu Rev Earth Planet Sci.

[R122] Hansen BMS (2008). On the absorption and redistribution of energy in irradiated planets. Astrophys J Suppl Ser.

[R123] Hansen BMS, Murray N (2012). Migration then assembly: Formation of Neptune-mass planets inside 1 AU. Astrophys J.

[R124] Hansen BMS, Murray N (2013). Testing in situ assembly with the Kepler planet candidate sample. Astrophys J.

[R125] Hansen CJ, Schwartz JC, Cowan NB (2014). Features in the broad-band eclipse spectra of exoplanets: Signal or noise?. Mon Not R Astron Soc.

[R126] Hauschildt PH, Allard F, Baron E (1999). The NextGen model atmosphere grid for 3000 ≤ *T_eff_* ≤ 10000 K. Astrophys J.

[R127] Haynes K, Mandell AM, Madhusudhan N, Deming D, Knutson H (2015). Spectroscopic evidence for a temperature inversion in the dayside atmosphere of hot Jupiter WASP-33b. Astrophys J.

[R128] Helled R, Bodenheimer P (2010). Metallicity of the massive protoplanets around HR 8799 if formed by gravitational instability. Icarus.

[R129] Helled R, Bodenheimer P (2014). The formation of Uranus and Neptune: Challenges and implications for intermediate-mass exoplanets. Astrophys J.

[R130] Helling C, Ackerman A, Allard F, Dehn M, Hauschildt P, Homeier D, Lodders K, Marley M, Rietmeijer F, Tsuji T, Woitke P (2008). A comparison of chemistry and dust cloud formation in ultracool dwarf model atmospheres. Mon Not R Astron Soc.

[R131] Helling C, Jardine M, Stark C, Diver D (2013). Ionization in atmospheres of brown dwarfs and extrasolar planets. III. Breakdown conditions for mineral clouds. Astrophys J.

[R132] Helling C, Woitke P, Rimmer PB, Kamp I, Thi W-F, Meijerink R (2014). Disk evolution, element abundances and cloud properties of young gas giant planets. Life.

[R133] Heng K, Demory B-O (2013). Understanding trends associated with clouds in irradiated exoplanets. Astrophys J.

[R134] Heng K, Kopparla P (2012). On the stability of super-Earth atmospheres. Astrophys J.

[R135] Heng K, Lyons JR (2016). Carbon dioxide in exoplanetary atmospheres: Rarely dominant compared to carbon monoxide and water in hot, hydrogen-dominated atmospheres. Astrophys J.

[R136] Heng K, Showman AP (2015). Atmospheric dynamics of hot exoplanets. Annu Rev Earth Planet Sci.

[R137] Heng K, Vogt SS (2011). Gliese 581g as a scaled-up version of Earth: Atmospheric circulation simulations. Mon Not R Astron Soc.

[R138] Heng K, Hayek W, Pont F, Sing DK (2012). On the effects of clouds and hazes in the atmospheres of hot Jupiters: Semi-analytical temperature-pressure profiles. Mon Not R Astron Soc.

[R139] Hinz PM, Rodigas TJ, Kenworthy MA, Sivanandam S, Heinze AN, Mamajek EE, Meyer MR (2010). Thermal infrared MMTAO observations of the HR 8799 planetary system. Astrophys J.

[R140] Hoeijmakers HJ, de Kok RJ, Snellen IAG, Brogi M, Birkby JL, Schwarz H (2015). A search for TiO in the optical high-resolution transmission spectrum of HD 209458b: Hindrance due to inaccuracies in the line database. Astron Astrophys.

[R141] Howard AW, Marcy GW, Bryson ST, Jenkins JM, Rowe JF, Batalha NM, Borucki WJ, Koch DG, Dunham EW, Gautier TN, Van Cleve J (2012). Planet occurrence within 0.25 AU of solar-type stars from Kepler. Astrophys J Suppl Ser.

[R142] Hu Y, Ding F (2011). Radiative constraints on the habitability of exoplanets Gliese 581c and Gliese 581d. Astron Astrophys.

[R143] Hu Y, Ding F (2013). Atmospheric circulations and climate of tidal-locking exoplanets. Sci Sinica Phys Mech Astron.

[R144] Hu R, Seager S (2014). Photochemistry in terrestrial exoplanet atmospheres. III. Photochemistry and thermochemistry in thick atmospheres on super Earths and mini Neptunes. Astrophys J.

[R145] Hu Y, Yang J (2014). Role of ocean heat transport in climates of tidally locked exoplanets around m dwarf stars. Proc Natl Acad Sci.

[R146] Hu R, Seager S, Bains W (2012). Photochemistry in terrestrial exoplanet atmospheres. I. Photochemistry model and benchmark cases. Astrophys J.

[R147] Hu R, Seager S, Bains W (2013). Photochemistry in terrestrial exoplanet atmospheres. II. H_2_S and SO_2_ photochemistry in anoxic atmospheres. Astrophys J.

[R148] Hu R, Demory B-O, Seager S, Lewis N, Showman AP (2015a). A semi-analytical model of visible-wavelength phase curves of exoplanets and applications to Kepler- 7 b and Kepler- 10 b. Astrophys J.

[R149] Hu R, Seager S, Yung YL (2015b). Helium atmospheres on warm Neptune- and sub-Neptune-sized exoplanets and applications to GJ 436b. Astrophys J.

[R150] Huang S-S (1960). Life-supporting regions in the vicinity of binary systems. Publications of the Astronomical Society of the Pacific.

[R151] Hubbard WB, Burrows A, Lunine JI (2002). Theory of giant planets. Annu Rev Astron Astrophys.

[R152] Hubeny I, Burrows A, Sudarsky D (2003). A possible bifurcation in atmospheres of strongly irradiated stars and planets. Astrophys J.

[R153] Huitson CM, Sing DK, Vidal-Madjar A, Ballester GE, Lecavelier des Etangs A, Désert J-M, Pont F (2012). Temperature-pressure profile of the hot Jupiter HD 189733b from HST sodium observations: Detection of upper atmospheric heating. Mon Not R Astron Soc.

[R154] Huitson CM, Sing DK, Pont F, Fortney JJ, Burrows AS, Wilson PA, Ballester GE, Nikolov N, Gibson NP, Deming D, Aigrain S (2013). An HST optical-to-near-IR transmission spectrum of the hot Jupiter WASP-19b: Detection of atmospheric water and likely absence of TiO. Mon Not R Astron Soc.

[R155] Ida S, Lin DNC (2005). Toward a deterministic model of planetary formation. III. Mass distribution of short-period planets around stars of various masses. Astrophys J.

[R156] Inamdar NK, Schlichting HE (2015). The formation of super-Earths and mini-Neptunes with giant impacts. Mon Not R Astron Soc.

[R157] Ingersoll AP (1969). The runaway greenhouse: A history of water on Venus. J Atmos Sci.

[R158] Iro N, Bézard B, Guillot T (2005). A time-dependent radiative model of HD 209458b. Astron Astrophys.

[R159] Ito Y, Ikoma M, Kawahara H, Nagahara H, Kawashima Y, Nakamoto T (2015). Theoretical emission spectra of atmospheres of hot rocky super-Earths. Astrophys J.

[R160] Janson M, Bergfors C, Goto M, Brandner W, Lafrenière D (2010). Spatially resolved spectroscopy of the exoplanet HR 8799 c. Astrophys J Lett.

[R161] Janson M, Brandt TD, Kuzuhara M, Spiegel DS, Thalmann C, Currie T, Bonnefoy M, Zimmerman N, Sorahana S, Kotani T, Schlieder J (2013). Direct imaging detection of methane in the atmosphere of GJ 504 b. Astrophys J Lett.

[R162] Jensen AG, Redfield S, Endl M, Cochran WD, Koesterke L, Barman T (2012). A detection of H*α* in an exoplanetary exosphere. Astrophys J.

[R163] Joshi M (2003). Climate model studies of synchronously rotating planets. Astrobiology.

[R164] Joshi M, Haberle R, Reynolds R (1997). Simulations of the atmospheres of synchronously rotating terrestrial planets orbiting m dwarfs: Conditions for atmospheric collapse and the implications for habitability. Icarus.

[R165] Kasting J (2010). How to Find a Habitable Planet. Science Essentials.

[R166] Kasting JF, Whitmire DP, Reynolds RT (1993). Habitable zones around main sequence stars. Icarus.

[R167] Kennedy GM, Kenyon SJ (2008). Planet formation around stars of various masses: Hot super-Earths. Astrophys J.

[R168] Kipping DM, Spiegel DS (2011). Detection of visible light from the darkest world. Mon Not R Astron Soc.

[R169] Kirkpatrick JD (2005). New spectral types L and T. Annu Rev Astron Astrophys.

[R170] Knutson HA, Charbonneau D, Allen LE, Fortney JJ, Agol E, Cowan NB, Showman AP, Cooper CS, Megeath ST (2007). A map of the day-night contrast of the extrasolar planet HD 189733b. Nature.

[R171] Knutson HA, Charbonneau D, Cowan NB, Fortney JJ, Showman AP, Agol E, Henry GW (2009). The 8 μm phase variation of the hot Saturn hd 149026b. Astrophys J.

[R172] Knutson HA, Lewis N, Fortney JJ, Burrows A, Showman AP, Cowan NB, Agol E, Aigrain S, Charbonneau D, Deming D, Désert J-M (2012). 3.6 and 4.5 μm phase curves and evidence for non-equilibrium chemistry in the atmosphere of extrasolar planet HD 189733b. Astrophys J.

[R173] Knutson HA, Benneke B, Deming D, Homeier D (2014a). A featureless transmission spectrum for the Neptune-mass exoplanet GJ436b. Nature.

[R174] Knutson HA, Dragomir D, Kreidberg L, Kempton EM-R, McCullough PR, Fortney JJ, Bean JL, Gillon M, Homeier D, Howard AW (2014b). Hubble Space Telescope near-IR transmission spectroscopy of the super-Earth HD 97658b. Astrophys J.

[R175] Konopacky QM, Barman TS, Macintosh BA, Marois C (2013). Detection of carbon monoxide and water absorption lines in an exoplanet atmosphere. Science.

[R176] Kopparapu RK (2013). A revised estimate of the occurrence rate of terrestrial planets in the habitable zones around Kepler m-dwarfs. Astrophys J Lett.

[R177] Kopparapu Rk, Kasting JF, Zahnle KJ (2012). A photochemical model for the carbon-rich planet WASP-12b. Astrophys J.

[R178] Koskinen TT, Harris MJ, Yelle RV, Lavvas P (2013). The escape of heavy atoms from the ionosphere of HD209458b. I. A photochemical-dynamical model of the thermosphere. Icarus.

[R179] Koskinen TT, Lavvas P, Harris MJ, Yelle RV (2014). Thermal escape from extrasolar giant planets. Philos Trans R Soc A Math Phys Eng Sci.

[R180] Kreidberg L, Bean JL, Désert J-M, Benneke B, Deming D, Stevenson KB, Seager S, Berta-Thompson Z, Seifahrt A, Homeier D (2014a). Clouds in the atmosphere of the super-Earth exoplanet GJ1214b. Nature.

[R181] Kreidberg L, Bean JL, Désert J-M, Line MR, Fortney JJ, Madhusudhan N, Stevenson KB, Showman AP, Charbonneau D, McCullough PR, Seager S (2014b). A precise water abundance measurement for the hot Jupiter WASP-43b. Astrophys J Lett.

[R182] Kreidberg L, Line MR, Bean JL, Stevenson KB, Désert J-M, Madhusudhan N, Fortney JJ, Barstow JK, Henry GW, Williamson MH, Showman AP (2015). A detection of water in the transmission spectrum of the hot Jupiter WASP-12b and implications for its atmospheric composition. Astrophys J.

[R183] Kuchner MJ, Seager S (2005). Extrasolar carbon planets. ArXiv Astrophysics e-prints.

[R184] Kurucz RL (1979). Model atmospheres for G, F, A, B, and O stars. Astrophys J Suppl Ser.

[R185] Lai D (2014). Star-disc-binary interactions in protoplanetary disc systems and primordial spin-orbit misalignments. Mon Not R Astron Soc.

[R186] Lammer H, Eybl V, Kislyakova K, Weingrill J, Holmström M, Khodachenko M, Kulikov YN, Reiners A, Leitzinger M, Odert P (2011). Uv transit observations of euv-heated expanded thermospheres of Earth-like exoplanets around m-stars: Testing atmosphere evolution scenarios. Astrophys Space Sci.

[R187] Lammer H, Stökl A, Erkaev NV, Dorfi EA, Odert P, Güdel M, Kulikov YN, Kislyakova KG, Leitzinger M (2014). Origin and loss of nebula-captured hydrogen envelopes from ‘sub’- to ‘super-Earths’ in the habitable zone of Sun-like stars. Mon Not R Astron Soc.

[R188] Lanotte AA, Gillon M, Demory B-O, Fortney JJ, Astudillo N, Bonfils X, Magain P, Delfosse X, Forveille T, Lovis C, Mayor M (2014). A global analysis of Spitzer and new HARPS data confirms the loneliness and metal-richness of GJ 436 b. Astron Astrophys.

[R189] Lara LM, Lellouch E, López-Moreno JJ, Rodrigo R (1996). Vertical distribution of Titan’s atmospheric neutral constituents. J Geophys Res.

[R190] Lavvas P, Koskinen T, Yelle RV (2011). Chemical composition of extrasolar giant planets. EPSC-DPS Joint Meeting.

[R191] Lavvas P, Koskinen T, Yelle RV (2014). Electron densities and alkali atoms in exoplanet atmospheres. Astrophys J.

[R192] Lecavelier Des Etangs A, Pont F, Vidal-Madjar A, Sing D (2008a). Rayleigh scattering in the transit spectrum of HD 189733b. Astron Astrophys.

[R193] Lecavelier Des Etangs A, Vidal-Madjar A, Désert J-M, Sing D (2008b). Rayleigh scattering by H_2_ in the extrasolar planet HD 209458b. Astron Astrophys.

[R194] Lecavelier Des Etangs A, Ehrenreich D, Vidal-Madjar A, Ballester GE, Désert J-M, Ferlet R, Hébrard G, Sing DK, Tchakoumegni K-O, Udry S (2010). Evaporation of the planet HD 189733b observed in H I Lyman-*α*. Astron Astrophys.

[R195] Leconte J, Forget F, Charnay B, Wordsworth R, Pottier A (2013). Increased insolation threshold for runaway greenhouse processes on Earth-like planets. Nature.

[R196] Leconte J, Forget F, Lammer H (2014). On the (anticipated) diversity of terrestrial planet atmospheres. Exp Astron.

[R197] Lee J-M, Fletcher LN, Irwin PGJ (2012). Optimal estimation retrievals of the atmospheric structure and composition of HD 189733b from secondary eclipse spectroscopy. Mon Not R Astron Soc.

[R198] Lee J-M, Heng K, Irwin PGJ (2013). Atmospheric retrieval analysis of the directly imaged exoplanet HR 8799b. Astrophys J.

[R199] Lee EJ, Chiang E, Ormel CW (2014). Make super-Earths, not Jupiters: Accreting nebular gas onto solid cores at 0.1 AU and beyond. Astrophys J.

[R200] Lee G, Helling C, Dobbs-Dixon I, Juncher D (2015). Modelling the local and global cloud formation on HD 189733b. Astron Astrophys.

[R201] Léger A, Grasset O, Fegley B, Codron F, Albarede AF, Barge P, Barnes R, Cance P, Carpy S, Catalano F, Cavarroc C (2011). The extreme physical properties of the CoRoT-7b super-Earth. Icarus.

[R202] Lewis JS (1969). Observability of spectroscopically active compounds in the atmosphere of Jupiter. Icarus.

[R203] Lewis NK, Showman AP, Fortney JJ, Marley MS, Freedman RS, Lodders K (2010). Atmospheric circulation of eccentric hot Neptune GJ436b. Astrophys J.

[R204] Liang M-C, Parkinson CD, Lee AY-T, Yung YL, Seager S (2003). Source of atomic hydrogen in the atmosphere of HD 209458b. Astrophys J Lett.

[R205] Liang M-C, Seager S, Parkinson CD, Lee AY-T, Yung YL (2004). On the insignificance of photochemical hydrocarbon aerosols in the atmospheres of close-in extrasolar giant planets. Astrophys J Lett.

[R206] Lindal GF (1992). The atmosphere of Neptune—An analysis of radio occultation data acquired with Voyager 2. Astron J.

[R207] Lindzen RS (1981). Turbulence and stress owing to gravity wave and tidal breakdown. J Geophys Res.

[R208] Line MR, Liang MC, Yung YL (2010). High-temperature photochemistry in the atmosphere of HD 189733b. Astrophys J.

[R209] Line MR, Vasisht G, Chen P, Angerhausen D, Yung YL (2011). Thermochemical and photochemical kinetics in cooler hydrogen-dominated extrasolar planets: A methane-poor GJ436b?. Astrophys J.

[R210] Line MR, Zhang X, Vasisht G, Natraj V, Chen P, Yung YL (2012). Information content of exoplanetary transit spectra: An initial look. Astrophys J.

[R211] Line MR, Wolf AS, Zhang X, Knutson H, Kammer JA, Ellison E, Deroo P, Crisp D, Yung YL (2013). A systematic retrieval analysis of secondary eclipse spectra. I. A comparison of atmospheric retrieval techniques. Astrophys J.

[R212] Linsky JL, Yang H, France K, Froning CS, Green JC, Stocke JT, Osterman SN (2010). Observations of mass loss from the transiting exoplanet HD 209458b. Astrophys J.

[R213] Lissauer JJ (1993). Planet formation. Annu Rev Astron Astrophys.

[R214] Lissauer JJ, Stevenson DJ (2007). Formation of giant planets. Protostars and Planets V.

[R215] Lockwood AC, Brown ME, Stansberry J (2014). The size and shape of the oblong dwarf planet Haumea. Earth Moon Planets.

[R216] Lodders K (1999). Alkali element chemistry in cool dwarf atmospheres. Astrophys J.

[R217] Lodders K (2002). Titanium and vanadium chemistry in low-mass dwarf stars. Astrophys J.

[R218] Lodders K (2004). Jupiter formed with more tar than ice. Astrophys J.

[R219] Lodders K, Barnes R (2010). Exoplanet Chemistry.

[R220] Lodders K, Fegley B (2002). Atmospheric chemistry in giant planets, brown dwarfs, and low-mass dwarf stars. I. Carbon, nitrogen, and oxygen. Icarus.

[R221] Lopez ED, Fortney JJ (2013). The role of core mass in controlling evaporation: The Kepler radius distribution and the Kepler-36 density dichotomy. Astrophys J.

[R222] Lopez ED, Fortney JJ (2014). Understanding the mass-radius relation for sub-Neptunes: Radius as a proxy for composition. Astrophys J.

[R223] Lopez ED, Fortney JJ, Miller N (2012). How thermal evolution and mass-loss sculpt populations of super-Earths and sub-Neptunes: Application to the Kepler-11 system and beyond. Astrophys J.

[R224] Luger R, Barnes R (2015). Extreme water loss and abiotic O2Buildup on planets throughout the habitable zones of M dwarfs. Astrobiology.

[R225] Luger R, Barnes R, Lopez E, Fortney J, Jackson B, Meadows V (2015). Habitable evaporated cores: Transforming mini-Neptunes into super-Earths in the habitable zones of M dwarfs. Astrobiology.

[R226] Lupu RE, Zahnle K, Marley MS, Schaefer L, Fegley B, Morley C, Cahoy K, Freedman R, Fortney JJ (2014). The atmospheres of earthlike planets after giant impact events. Astrophys J.

[R227] Macintosh B, Graham JR, Barman T, De Rosa RJ, Konopacky Q, Marley MS, Marois C, Nielsen EL, Pueyo L, Rajan A, Rameau J (2015). Discovery and spectroscopy of the young Jovian planet 51 Eri b with the Gemini Planet Imager. Science.

[R228] Madhusudhan N (2012). C/O ratio as a dimension for characterizing exoplanetary atmospheres. Astrophys J.

[R229] Madhusudhan N, Redfield S (2015). Optimal measures for characterizing water-rich super-Earths. Int J Astrobiol.

[R230] Madhusudhan N, Seager S (2009). A temperature and abundance retrieval method for exoplanet atmospheres. Astrophys J.

[R231] Madhusudhan N, Seager S (2011). High metallicity and non-equilibrium chemistry in the dayside atmosphere of hot-Neptune GJ 436b. Astrophys J.

[R232] Madhusudhan N, Burrows A, Currie T (2011a). Model atmospheres for massive gas giants with thick clouds: Application to the HR 8799 planets and predictions for future detections. Astrophys J.

[R233] Madhusudhan N, Harrington J, Stevenson KB, Nymeyer S, Campo CJ, Wheatley PJ, Deming D, Blecic J, Hardy RA, Lust NB, Anderson DR (2011b). A high C/O ratio and weak thermal inversion in the atmosphere of exoplanet WASP-12b. Nature.

[R234] Madhusudhan N, Amin MA, Kennedy GM (2014a). Toward chemical constraints on hot Jupiter migration. Astrophys J Lett.

[R235] Madhusudhan N, Crouzet N, McCullough PR, Deming D, Hedges C (2014b). H_2_O abundances in the atmospheres of three hot Jupiters. Astrophys J Lett.

[R236] Madhusudhan N, Knutson H, Fortney JJ, Barman T (2014c). Exoplanetary atmospheres. Protostars and Planets VI.

[R237] Mahaffy PR, Niemann HB, Alpert A, Atreya SK, Demick J, Donahue TM, Harpold DN, Owen TC (2000). Noble gas abundance and isotope ratios in the atmosphere of Jupiter from the Galileo probe mass spectrometer. J Geophys Res.

[R238] Mancini L, Ciceri S, Chen G, Tregloan-Reed J, Fortney JJ, Southworth J, Tan TG, Burgdorf M, Calchi Novati S, Dominik M, Fang X-S (2013). Physical properties, transmission and emission spectra of the WASP-19 planetary system from multi-colour photometry. Mon Not R Astron Soc.

[R239] Mandell AM, Haynes K, Sinukoff E, Madhusudhan N, Burrows A, Deming D (2013). Exoplanet transit spectroscopy using WFC3: WASP-12 b, WASP-17 b, and WASP-19 b. Astrophys J.

[R240] Marboeuf U, Thiabaud A, Alibert Y, Cabral N, Benz W (2014). From stellar nebula to planetesimals. Astron Astrophys.

[R241] Marcy GW, Isaacson H, Howard AW, Rowe JF, Jenkins JM, Bryson ST, Latham DW, Howell SB, Gautier TN, Batalha NM, Rogers L (2014). Masses, radii, and orbits of small Kepler planets: The transition from gaseous to rocky planets. Astrophys J Suppl Ser.

[R242] Marley MS, Robinson TD (2015). On the cool side: Modeling the atmospheres of brown dwarfs and giant planets. Annu Rev Astron Astrophys.

[R243] Marley MS, Saumon D, Guillot T, Freedman RS, Hubbard WB, Burrows A, Lunine JI (1996). Atmospheric, evolutionary, and spectral models of the brown dwarf Gliese 229 B. Science.

[R244] Marley MS, Ackerman AS, Cuzzi JN, Kitzmann D, Mackwell SJ, Simon-Miller AA, Harder JW, Bullock MA (2013). Clouds and hazes in exoplanet atmospheres. Comparative Climatology of Terrestrial Planets.

[R245] Martins JHC, Santos NC, Figueira P, Faria JP, Montalto M, Boisse I, Ehrenreich D, Lovis C, Mayor M, Melo C, Pepe F (2015). Evidence for a spectroscopic direct detection of reflected light from <ASTROBJ>51 Pegasi b</ASTROBJ>. Astron Astrophys.

[R246] Massol (2016). Space Sci Rev.

[R247] Matousek S (2007). The Juno new frontiers mission. Acta Astronaut.

[R248] Mayor M, Marmier M, Lovis C, Udry S, Ségransan D, Pepe F, Benz W, Bertaux J-, Bouchy F, Dumusque X, Lo Curto G (2011). The HARPS search for southern extrasolar planets XXXIV. Occurrence, mass distribution and orbital properties of super-Earths and Neptune-mass planets. ArXiv e-prints.

[R249] McCullough P, MacKenty J (2012). Considerations for using Spatial Scans with WFC3. Technical report.

[R250] McCullough PR, Crouzet N, Deming D, Madhusudhan N (2014). Water vapor in the spectrum of the extrasolar planet HD 189733b I. The transit Astrophys J.

[R251] McKay CP, Pollack JB, Courtin R (1989). The thermal structure of Titan’s atmosphere. Icarus.

[R252] Mellor GL, Yamada T (1982). Development of a turbulence closure model for geophysical fluid problems. Rev Geophys Space Phys.

[R253] Menou K (2013). Water-trapped worlds. Astrophys J.

[R254] Miguel Y, Kaltenegger L (2014). Exploring atmospheres of hot mini-Neptunes and extrasolar giant planets orbiting different stars with application to HD 97658b, WASP-12b, CoRoT-2b, XO-1b, and HD 189733b. Astrophys J.

[R255] Miguel Y, Kaltenegger L, Fegley B, Schaefer L (2011). Compositions of hot super-Earth atmospheres: Exploring Kepler candidates. Astrophys J Lett.

[R256] Miguel Y, Kaltenegger L, Linsky JL, Rugheimer S (2015). The effect of Lyman *α* radiation on mini-Neptune atmospheres around M stars: Application to GJ 436b. Mon Not R Astron Soc.

[R257] Miller N, Fortney JJ (2011). The heavy-element masses of extrasolar giant planets, revealed. Astrophys J Lett.

[R258] Miller-Ricci Kempton E, Zahnle K, Fortney JJ (2012). The atmospheric chemistry of GJ 1214b: Photochemistry and clouds. Astrophys J.

[R259] Miller-Ricci E, Seager S, Sasselov D (2009). The atmospheric signatures of super-Earths: How to distinguish between hydrogen-rich and hydrogen-poor atmospheres. Astrophys J.

[R260] Morbidelli A, Lunine JI, O’Brien DP, Raymond SN, Walsh KJ (2012). Building terrestrial planets. Annu Rev Earth Planet Sci.

[R261] Mordasini C, Alibert Y, Georgy C, Dittkrist K-M, Klahr H, Henning T (2012). Characterization of exoplanets from their formation. II. The planetary mass-radius relationship. Astron Astrophys.

[R262] Morley CV, Fortney JJ, Kempton EM-R, Marley MS, Visscher C, Zahnle K (2013). Quantitatively assessing the role of clouds in the transmission spectrum of GJ 1214b. Astrophys J.

[R263] Moses JI (2014). Chemical kinetics on extrasolar planets. Philos Trans R Soc A Math Phys Eng Sci.

[R264] Moses JI, Bézard B, Lellouch E, Gladstone GR, Feuchtgruber H, Allen M (2000). Photochemistry of Saturn’s atmosphere. I. Hydrocarbon chemistry and comparisons with ISO observations. Icarus.

[R265] Moses JI, Visscher C, Fortney JJ, Showman AP, Lewis NK, Griffith CA, Klippenstein SJ, Shabram M, Friedson AJ, Marley MS, Freedman RS (2011). Disequilibrium carbon, oxygen, and nitrogen chemistry in the atmospheres of HD 189733b and HD 209458b. Astrophys J.

[R266] Moses JI, Line MR, Visscher C, Richardson MR, Nettelmann N, Fortney JJ, Barman TS, Stevenson KB, Madhusudhan N (2013a). Compositional diversity in the atmospheres of hot Neptunes, with application to GJ 436b. Astrophys J.

[R267] Moses JI, Madhusudhan N, Visscher C, Freedman RS (2013b). Chemical consequences of the C/O ratio on hot Jupiters: Examples from WASP-12b, CoRoT-2b, XO-1b, and HD 189733b. Astrophys J.

[R268] Mousis O, Lunine JI, Madhusudhan N, Johnson TV (2012). Nebular water depletion as the cause of Jupiter’s low oxygen abundance. Astrophys J Lett.

[R269] Murgas F, Pallé E, Zapatero Osorio MR, Nortmann L, Hoyer S, Cabrera-Lavers A (2014). The GTC exoplanet transit spectroscopy survey. I. OSIRIS transmission spectroscopy of the short period planet WASP-43b. Astron Astrophys.

[R270] Nettelmann N, Kramm U, Redmer R, Neuhäuser R (2010). Interior structure models of GJ 436b. Astron Astrophys.

[R271] Nikolov N, Sing DK, Pont F, Burrows AS, Fortney JJ, Ballester GE, Evans TM, Huitson CM, Wakeford HR, Wilson PA, Aigrain S (2014). Hubble Space Telescope hot Jupiter transmission spectral survey: A detection of Na and strong optical absorption in HAT-P-1b. Mon Not R Astron Soc.

[R272] Nikolov N, Sing DK, Burrows AS, Fortney JJ, Henry GW, Pont F, Ballester GE, Aigrain S, Wilson PA, Huitson CM, Gibson NP (2015). HST hot-Jupiter transmission spectral survey: Haze in the atmosphere of WASP-6b. Mon Not R Astron Soc.

[R273] Öberg KI, Murray-Clay R, Bergin EA (2011). The effects of snowlines on C/O in planetary atmospheres. Astrophys J Lett.

[R274] Oberg (2016). Space Sci Rev.

[R275] Ogilvie GI (2014). Tidal dissipation in stars and giant planets. Annu Rev Astron Astrophys.

[R276] Oppenheimer BR, Baranec C, Beichman C, Brenner D, Burruss R, Cady E, Crepp JR, Dekany R, Fergus R, Hale D, Hillenbrand L (2013). Reconnaissance of the HR 8799 exosolar system. I. Near-infrared spectroscopy. Astrophys J.

[R277] Owen JE, Wu Y (2013). Kepler planets: A tale of evaporation. Astrophys J.

[R278] Owen T, Mahaffy P, Niemann HB, Atreya S, Donahue T, Bar-Nun A, de Pater I (1999). A low-temperature origin for the planetesimals that formed Jupiter. Nature.

[R279] Papaloizou JCB, Terquem C (2006). Planet formation and migration. Rep Prog Phys.

[R280] Papaloizou JCB, Nelson RP, Kley W, Masset FS, Artymowicz P (2007). Disk-planet interactions during planet formation. Protostars and Planets V.

[R281] Parmentier V, Guillot T (2014). A non-grey analytical model for irradiated atmospheres. I. Derivation. Astron Astrophys.

[R282] Parmentier V, Showman AP, Lian Y (2013). 3D mixing in hot Jupiters atmospheres. I. Application to the day/night cold trap in HD 209458b. Astron Astrophys.

[R283] Parmentier V, Guillot T, Fortney JJ, Marley MS (2015). A non-grey analytical model for irradiated atmospheres. II. Analytical vs. numerical solutions. Astron Astrophys.

[R284] Peixoto J, Oort AH (1992). Physics of Climate.

[R285] Pepin RO (2006). Atmospheres on the terrestrial planets: Clues to origin and evolution. Earth Planet Sci Lett.

[R286] Phillips NA (1956). The general circulation of the atmosphere: A numerical experiment. Q J R Meteorol Soc.

[R287] Pierrehumbert RT (2011). A palette of climates for Gliese 581g. Astrophys J Lett.

[R288] Pollack JB, Hubickyj O, Bodenheimer P, Lissauer JJ, Podolak M (1996). Y Greenzweig, Formation of the giant planets by concurrent accretion of solids and gas. Icarus.

[R289] Pont F, Knutson H, Gilliland RL, Moutou C, Charbonneau D (2008). Detection of atmospheric haze on an extrasolar planet: The 0.55-1.05 μm transmission spectrum of HD 189733b with the Hubble Space Telescope. Mon Not R Astron Soc.

[R290] Pont F, Sing DK, Gibson NP, Aigrain S, Henry G, Husnoo N (2013). The prevalence of dust on the exoplanet HD 189733b from Hubble and Spitzer observations. Mon Not R Astron Soc.

[R291] Prinn RG, Barshay SS (1977). Carbon monoxide on Jupiter and implications for atmospheric convection. Science.

[R292] Rafikov RR (2005). Can giant planets form by direct gravitational instability?. Astrophys J Lett.

[R293] Ranjan S, Charbonneau D, Désert J-M, Madhusudhan N, Deming D, Wilkins A, Mandell AM (2014). Atmospheric characterization of five hot Jupiters with the wide field camera 3 on the Hubble Space Telescope. Astrophys J.

[R294] Rasio FA, Ford EB (1996). Dynamical instabilities and the formation of extrasolar planetary systems. Science.

[R295] Rauer H, Catala C, Aerts C, Appourchaux T, Benz W, Brandeker A, Christensen-Dalsgaard J, Deleuil M, Gizon L, Goupil M-J, Güdel M (2014). The PLATO 2.0 mission. Exp Astron.

[R296] Redfield S, Endl M, Cochran WD, Koesterke L (2008). Sodium absorption from the exoplanetary atmosphere of HD 189733b detected in the optical transmission spectrum. Astrophys J Lett.

[R297] Ricker GR, Winn JN, Vanderspek R, Latham DW, Bakos GÁ, Bean JL, Berta-Thompson ZK, Brown TM, Buchhave L, Butler NR, Butler RP (2015). Transiting Exoplanet Survey Satellite (TESS). J Astron Telesc Instrum Syst.

[R298] Rimmer PB, Helling C (2013). Ionization in atmospheres of brown dwarfs and extrasolar planets. IV. The effect of cosmic rays. Astrophys J.

[R299] Rimmer PB, Helling C (2015). A chemical kinetics network for lightning and life in planetary atmospheres. ArXiv e-prints.

[R300] Rimmer PB, Helling C, Bilger C (2014). The influence of galactic cosmic rays on ion-neutral hydrocarbon chemistry in the upper atmospheres of free-floating exoplanets. Int J Astrobiol.

[R301] Robinson TD, Catling DC (2012). An analytic radiative-convective model for planetary atmospheres. Astrophys J.

[R302] Rodler F, Lopez-Morales M, Ribas I (2012). Weighing the non-transiting hot Jupiter τ Boo b. Astrophys J Lett.

[R303] Rodler F, Kürster M, Barnes JR (2013). Detection of CO absorption in the atmosphere of the hot Jupiter HD 189733b. Mon Not R Astron Soc.

[R304] Rodono M (1986). The atmospheres of m dwarfs: Observations. NASA Spec Publ.

[R305] Rogers LA (2015). Most 1.6 Earth-radius planets are not rocky. Astrophys J.

[R306] Rogers LA, Bodenheimer P, Lissauer JJ, Seager S (2011). Formation and structure of low-density exo-Neptunes. Astrophys J.

[R307] Rossow WB (1978). Cloud microphysics—Analysis of the clouds of Earth, Venus, Mars, and Jupiter. Icarus.

[R308] Rowe JF, Matthews JM, Seager S, Miller-Ricci E, Sasselov D, Kuschnig R, Guenther DB, Moffat AFJ, Rucinski SM, Walker GAH, Weiss WW (2008). The very low albedo of an extrasolar planet: MOST space-based photometry of HD 209458. Astrophys J.

[R309] Rowe JF, Coughlin JL, Antoci V, Barclay T, Batalha NM, Borucki WJ, Burke CJ, Bryson ST, Caldwell DA, Campbell JR, Catanzarite JH (2015). Planetary candidates observed by Kepler. V. Planet sample from Q1-Q12 (36 months). Astrophys J Suppl Ser.

[R310] Sanz-Forcada J, Micela G, Ribas I, Pollock AMT, Eiroa C, Velasco A, Solano E, García-Álvarez D (2011). Estimation of the XUV radiation onto close planets and their evaporation. Astron Astrophys.

[R311] Schaefer L, Fegley B (2011). Atmospheric chemistry of Venus-like exoplanets. Astrophys J.

[R312] Schaefer L, Fegley B (2009). Chemistry of silicate atmospheres of evaporating super-Earths. Astrophys J Lett.

[R313] Schaefer L, Fegley B (2010). Chemistry of atmospheres formed during accretion of the Earth and other terrestrial planets. Icarus.

[R314] Schaefer L, Lodders K, Fegley B (2012). Vaporization of the Earth: Application to exoplanet atmospheres. Astrophys J.

[R315] Schlawin E, Agol E, Walkowicz LM, Covey K, Lloyd JP (2010). Exoplanetary transits of limb-brightened lines: Tentative Si IV absorption by HD 209458b. Astrophys J Lett.

[R316] Schlawin E, Zhao M, Teske JK, Herter T (2014). A 0.8-2.4 μm transmission spectrum of the hot Jupiter CoRoT-1b. Astrophys J.

[R317] Schulze-Makuch D, Méndez A, Fairén AG, Von Paris P, Turse C, Boyer G, Davila AF, António MRdS, Catling D, Irwin LN (2011). A two-tiered approach to assessing the habitability of exoplanets. Astrobiology.

[R318] Schwartz JC, Cowan NB (2015). Balancing the energy budget of short-period giant planets: Evidence for reflective clouds and optical absorbers. Mon Not R Astron Soc.

[R319] Seager S, Sasselov DD (1998). Extrasolar giant planets under strong stellar irradiation. Astrophys J Lett.

[R320] Seager S, Sasselov DD (2000). Theoretical transmission spectra during extrasolar giant planet transits. Astrophys J.

[R321] Seager S, Richardson LJ, Hansen BMS, Menou K, Cho JY-K, Deming D (2005). On the dayside thermal emission of hot Jupiters. Astrophys J.

[R322] Segura A, Walkowicz LM, Meadows V, Kasting J, Hawley S (2010). The effect of a strong stellar flare on the atmospheric chemistry of an Earth-like planet orbiting an M dwarf. Astrobiology.

[R323] Selsis F, Kasting J, Levrard B, Paillet J, Ribas I, Delfosse X (2007). Habitable planets around the star Gliese 581? Astron. Astrophys.

[R324] Shaikhislamov IF, Khodachenko ML, Sasunov YL, Lammer H, Kislyakova KG, Erkaev NV (2014). Atmosphere expansion and mass loss of close-orbit giant exoplanets heated by stellar XUV. I. Modeling of hydrodynamic escape of upper atmospheric material. Astrophys J.

[R325] Shapley H (1953). Climatic Change: Evidence, Causes, and Effects.

[R326] Showman AP, Guillot T (2002). Atmospheric circulation and tides of “51 Pegasus b-like” planets. Astron Astrophys.

[R327] Showman AP, Polvani LM (2011). Equatorial superrotation on tidally locked exoplanets. Astrophys J.

[R328] Showman AP, Fortney JJ, Lian Y, Marley MS, Freedman RS, Knutson HA, Charbonneau D (2009). Atmospheric circulation of hot Jupiters: Coupled radiative-dynamical general circulation model simulations of HD 189733b and HD 209458b. Astrophys J.

[R329] Showman AP, Cho JY-K, Menou K, Seager S (2010). Atmospheric circulation of exoplanets. Exoplanets.

[R330] Showman AP, Wordsworth RD, Merlis TM, Kaspi Y, Mackwell SJ, Simon-Miller AA, Harder JW, Bullock MA (2013). Atmospheric circulation of terrestrial exoplanets. Comparative Climatology of Terrestrial Planetsed.

[R331] Sing DK, López-Morales M (2009). Ground-based secondary eclipse detection of the very-hot Jupiter OGLE-TR-56b. Astron Astrophys.

[R332] Sing DK, Désert J-M, Lecavelier Des Etangs A, Ballester GE, Vidal-Madjar A, Parmentier V, Hebrard G, Henry GW (2009). Transit spectrophotometry of the exoplanet HD 189733b. I. Searching for water but finding haze with HST NICMOS. Astron Astrophys.

[R333] Sing DK, Désert J-M, Fortney JJ, Lecavelier Des Etangs A, Ballester GE, Cepa J, Ehrenreich D, López-Morales M, Pont F, Shabram M, Vidal-Madjar A (2011a). Gran Telescopio Canarias OSIRIS transiting exoplanet atmospheric survey: Detection of potassium in XO-2b from narrowband spectrophotometry. Astron Astrophys.

[R334] Sing DK, Pont F, Aigrain S, Charbonneau D, Désert J-M, Gibson N, Gilliland R, Hayek W, Henry G, Knutson H, Lecavelier Des Etangs A (2011b). Hubble Space Telescope transmission spectroscopy of the exoplanet HD 189733b: High-altitude atmospheric haze in the optical and near-ultraviolet with STIS. Mon Not R Astron Soc.

[R335] Sing DK, Huitson CM, Lopez-Morales M, Pont F, Désert J-M, Ehrenreich D, Wilson PA, Ballester GE, Fortney JJ, Lecavelier des Etangs A, Vidal-Madjar A (2012). GTC OSIRIS transiting exoplanet atmospheric survey: Detection of sodium in XO-2b from differential long-slit spectroscopy. Mon Not R Astron Soc.

[R336] Sing DK, Lecavelier des Etangs A, Fortney JJ, Burrows AS, Pont F, Wakeford HR, Ballester GE, Nikolov N, Henry GW, Aigrain S, Deming D (2013). HST hot-Jupiter transmission spectral survey: Evidence for aerosols and lack of TiO in the atmosphere of WASP-12b. Mon Not R Astron Soc.

[R337] Sing DK, Wakeford HR, Showman AP, Nikolov N, Fortney JJ, Burrows AS, Ballester GE, Deming D, Aigrain S, Désert J-M, Gibson NP (2015). HST hot-Jupiter transmission spectral survey: Detection of potassium in WASP-31b along with a cloud deck and Rayleigh scattering. Mon Not R Astron Soc.

[R338] Skemer AJ, Hinz PM, Esposito S, Burrows A, Leisenring J, Skrutskie M, Desidera S, Mesa D, Arcidiacono C, Mannucci F, Rodigas TJ (2012). First light LBT AO images of HR 8799 bcde at 1.6 and 3.3 μm: New discrepancies between young planets and old brown dwarfs. Astrophys J.

[R339] Smith MD (1998). Estimation of a length scale to use with the quench level approximation for obtaining chemical abundances. Icarus.

[R340] Snellen IAG, de Kok RJ, de Mooij EJW, Albrecht S (2010). The orbital motion, absolute mass and high-altitude winds of exoplanet HD209458b. Nature.

[R341] Snellen IAG, Brandl BR, de Kok RJ, Brogi M, Birkby J, Schwarz H (2014). Fast spin of the young extrasolar planet *β* Pictoris b. Nature.

[R342] Spiegel DS, Silverio K, Burrows A (2009). Can TiO explain thermal inversions in the upper atmospheres of irradiated giant planets?. Astrophys J.

[R343] Spiegel DS, Burrows A, Ibgui L, Hubeny I, Milsom JA (2010). Models of Neptune-mass exoplanets: Emergent fluxes and albedos. Astrophys J.

[R344] Stark CR, Helling C, Diver DA, Rimmer PB (2014). Electrostatic activation of prebiotic chemistry in substellar atmospheres. Int J Astrobiol.

[R345] Stevenson DJ, Lunine JI (1988). Rapid formation of Jupiter by diffuse redistribution of water vapor in the solar nebula. Icarus.

[R346] Stevenson KB, Harrington J, Nymeyer S, Madhusudhan N, Seager S, Bowman WC, Hardy RA, Deming D, Rauscher E, Lust NB (2010). Possible thermochemical disequilibrium in the atmosphere of the exoplanet GJ 436b. Nature.

[R347] Stevenson KB, Bean JL, Madhusudhan N, Harrington J (2014a). Deciphering the atmospheric composition of WASP-12b: A comprehensive analysis of its dayside emission. Astrophys J.

[R348] Stevenson KB, Bean JL, Seifahrt A, Désert J-M, Madhusudhan N, Bergmann M, Kreidberg L, Homeier D (2014b). Transmission spectroscopy of the hot Jupiter WASP-12b from 0.7 to 5 μm. Astron J.

[R349] Stevenson KB, Désert J-M, Line MR, Bean JL, Fortney JJ, Showman AP, Kataria T, Kreidberg L, McCullough PR, Henry GW, Charbonneau D (2014c). Thermal structure of an exoplanet atmosphere from phase-resolved emission spectroscopy. Science.

[R350] Stökl A, Dorfi E, Lammer H (2015). Hydrodynamic simulations of captured protoatmospheres around Earth-like planets. Astron Astrophys.

[R351] Strobel DF (1981). Parameterization of linear wave chemical transport in planetary atmospheres by eddy diffusion. J Geophys Res.

[R352] Sudarsky D, Burrows A, Pinto P (2000). Albedo and reflection spectra of extrasolar giant planets. Astrophys J.

[R353] Sudarsky D, Burrows A, Hubeny I (2003). Theoretical spectra and atmospheres of extrasolar giant planets. Astrophys J.

[R354] Swain MR, Vasisht G, Tinetti G (2008). The presence of methane in the atmosphere of an extrasolar planet. Nature.

[R355] Swain MR, Tinetti G, Vasisht G, Deroo P, Griffith C, Bouwman J, Chen P, Yung Y, Burrows A, Brown LR, Matthews J (2009a). Water, methane, and carbon dioxide present in the dayside spectrum of the exoplanet HD 209458b. Astrophys J.

[R356] Swain MR, Vasisht G, Tinetti G, Bouwman J, Chen P, Yung Y, Deming D, Deroo P (2009b). Molecular signatures in the near-infrared dayside spectrum of HD 189733b. Astrophys J Lett.

[R357] Swain M, Deroo P, Tinetti G, Hollis M, Tessenyi M, Line M, Kawahara H, Fujii Y, Showman AP, Yurchenko SN (2013). Probing the extreme planetary atmosphere ofWASP-12b. Icarus.

[R358] Swain MR, Line MR, Deroo P (2014). On the detection of molecules in the atmosphere of HD 189733b using HST NICMOS transmission spectroscopy. Astrophys J.

[R359] Terquem C, Papaloizou JCB (2007). Migration and the formation of systems of hot super-Earths and Neptunes. Astrophys J.

[R360] Tian F (2009). Thermal escape from super Earth atmospheres in the habitable zones of m stars. Astrophys J.

[R361] Tinetti G, Vidal-Madjar A, Liang M-C, Beaulieu J-P, Yung Y, Carey S, Barber RJ, Tennyson J, Ribas I, Allard N, Ballester GE (2007). Water vapour in the atmosphere of a transiting extrasolar planet. Nature.

[R362] Tinetti G, Deroo P, Swain MR, Griffith CA, Vasisht G, Brown LR, Burke C, McCullough P (2010). Probing the terminator region atmosphere of the hot-Jupiter XO-1b with transmission spectroscopy. Astrophys J Lett.

[R363] Todorov KO, Deming D, Burrows A, Grillmair CJ (2014). Updated Spitzer emission spectroscopy of bright transiting hot Jupiter HD 189733b. Astrophys J.

[R364] Todorov KO, Line MR, Pineda JE, Meyer MR, Quanz SP, Hinkley S, Fortney JJ (2015). The water abundance of the directly imaged substellar companion {\kappa} and b retrieved from a near infrared spectrum. ArXiv e-prints.

[R365] Tomasko MG, Archinal B, Becker T, Bézard B, Bushroe M, Combes M, Cook D, Coustenis A, de Bergh C, Dafoe LE, Doose L (2005). Rain, winds and haze during the Huygens probe’s descent to Titan’s surface. Nature.

[R366] Triaud AHMJ, Collier Cameron A, Queloz D, Anderson DR, Gillon M, Hebb L, Hellier C, Loeillet B, Maxted PFL, Mayor M, Pepe F (2010). Spin-orbit angle measurements for six southern transiting planets. New insights into the dynamical origins of hot Jupiters. Astron Astrophys.

[R367] Tsuji T (1973). Molecular abundances in stellar atmospheres. II. Astron Astrophys.

[R368] Tsuji T, Ohnaka K, Aoki W, Nakajima T (1996). Evolution of dusty photospheres through red to brown dwarfs: How dust forms in very low mass objects. Astron Astrophys.

[R369] Udry S, Bonfils X, Delfosse X, Forveille T, Mayor M, Perrier C, Bouchy F, Lovis C, Pepe F, Queloz D (2007). The harps search for southern extra-solar planets *, **. Astron Astrophys.

[R370] van Dishoeck EF, Bergin EA, Lis DC, Lunine JI (2014). Water: From clouds to planets. Protostars and Planets VI.

[R371] Venot O, Hébrard E, Agúndez M, Dobrijevic M, Selsis F, Hersant F, Iro N, Bounaceur R (2012). A chemical model for the atmosphere of hot Jupiters. Astron Astrophys.

[R372] Venot O, Fray N, Bénilan Y, Gazeau M-C, Hébrard E, Larcher G, Schwell M, Dobrijevic M, Selsis F (2013). High-temperature measurements of VUV-absorption cross sections of CO_2_ and their application to exoplanets. Astron Astrophys.

[R373] Venot O, Agúndez M, Selsis F, Tessenyi M, Iro N (2014). The atmospheric chemistry of the warm Neptune GJ 3470b: Influence of metallicity and temperature on the CH_4_/CO ratio. Astron Astrophys.

[R374] Venot O, Hébrard E, Agúndez M, Decin L, Bounaceur R (2015). New chemical scheme for studying carbon-rich exoplanet atmospheres. Astron Astrophys.

[R375] Venturini J, Alibert Y, Benz W, Ikoma M (2015). Critical core mass for enriched envelopes: The role of H_2_O condensation. Astron Astrophys.

[R376] Vidal-Madjar A, Lecavelier des Etangs A, Désert J-M, Ballester GE, Ferlet R, Hébrard G, Mayor M (2003). An extended upper atmosphere around the extrasolar planet HD209458b. Nature.

[R377] Vidal-Madjar A, Désert J-M, Lecavelier des Etangs A, Hébrard G, Ballester GE, Ehrenreich D, Ferlet R, McConnell JC, Mayor M, Parkinson CD (2004). Detection of oxygen and carbon in the hydrodynamically escaping atmosphere of the extrasolar planet HD 209458b. Astrophys J Lett.

[R378] Vidal-Madjar A, Sing DK, Lecavelier Des Etangs A, Ferlet R, Désert J-M, Hébrard G, Boisse I, Ehrenreich D, Moutou C (2011). The upper atmosphere of the exoplanet HD 209458 b revealed by the sodium D lines. Temperature-pressure profile, ionization layer, and thermosphere. Astron Astrophys.

[R379] Vidal-Madjar A, Huitson CM, Bourrier V, Désert J-M, Ballester G, Lecavelier des Etangs A, Sing DK, Ehrenreich D, Ferlet R, Hébrard G, McConnell JC (2013). Magnesium in the atmosphere of the planet HD 209458 b: Observations of the thermosphere-exosphere transition region. Astron Astrophys.

[R380] Visscher C, Moses JI (2011). Quenching of carbon monoxide and methane in the atmospheres of cool brown dwarfs and hot Jupiters. Astrophys J.

[R381] Visscher C, Lodders K, Fegley B (2006). Atmospheric chemistry in giant planets, brown dwarfs, and low-mass dwarf stars. II. Sulfur and phosphorus. Astrophys J.

[R382] Visscher C, Lodders K, Fegley B (2010a). Atmospheric chemistry in giant planets, brown dwarfs, and low-mass dwarf stars. III. Iron, magnesium, and silicon. Astrophys J.

[R383] Visscher C, Moses JI, Saslow SA (2010b). The deep water abundance on Jupiter: New constraints from thermochemical kinetics and diffusion modeling. Icarus.

[R384] Von Bloh W, Bounama C, Cuntz M, Franck S (2007). The habitability of super-earths in Gliese 581. Astron Astrophys.

[R385] Von Paris P, Gebauer S, Godolt M, Rauer H, Stracke B (2011). Atmospheric studies of habitability in the Gliese 581 system. Astron Astrophys.

[R386] Waite JH, Young DT, Cravens TE, Coates AJ, Crary FJ, Magee B, Westlake J (2007). The process of tholin formation in Titan’s upper atmosphere. Science.

[R387] Wakeford HR, Sing DK, Deming D, Gibson NP, Fortney JJ, Burrows AS, Ballester G, Nikolov N, Aigrain S, Henry G, Knutson H (2013). HST hot Jupiter transmission spectral survey: Detection of water in HAT-P-lb from WFC3 near-IR spatial scan observations. Mon Not R Astron Soc.

[R388] Waldmann IP, Tinetti G, Deroo P, Hollis MDJ, Yurchenko SN, Tennyson J (2013). Blind extraction of an exoplanetary spectrum through independent component analysis. Astrophys J.

[R389] Waldmann IP, Tinetti G, Rocchetto M, Barton EJ, Yurchenko SN, Tennyson J (2015). Tau-REx I: A next generation retrieval code for exoplanetary atmospheres. Astrophys J.

[R390] Wang Y, Tian F, Hu Y (2014). Climate patterns of habitable exoplanets in eccentric orbits around m dwarfs. Astrophys J Lett.

[R391] Wang D, Gierasch PJ, Lunine JI, Mousis O (2015). New insights on Jupiter’s deep water abundance from disequilibrium species. Icarus.

[R392] Weiss LM, Marcy GW (2014). The mass-radius relation for 65 exoplanets smaller than 4 Earth radii. Astrophys J Lett.

[R393] Weiss LM, Marcy GW, Rowe JF, Howard AW, Isaacson H, Fortney JJ, Miller N, Demory B-O, Fischer DA, Adams ER, Dupree AK (2013). The mass of KOI-94d and a relation for planet radius, mass, and incident flux. Astrophys J.

[R394] Wilkins AN, Deming D, Madhusudhan N, Burrows A, Knutson H, McCullough P, Ranjan S (2014). The emergent 1.1-1.7 μm spectrum of the exoplanet CoRoT-2b as measured using the Hubble Space Telescope. Astrophys J.

[R395] Wilson PA, Sing DK, Nikolov N, Lecavelier des Etangs A, Pont F, Fortney JJ, Ballester GE, López-Morales M, Désert J-M, Vidal-Madjar A (2015). GTC OSIRIS transiting exoplanet atmospheric survey: Detection of potassium in HAT-P-1b from narrow-band spectrophotometry. Mon Not R Astron Soc.

[R396] Winn JN, Fabrycky D, Albrecht S, Johnson JA (2010). Hot stars with hot Jupiters have high obliquities. Astrophys J Lett.

[R397] Wolfgang A, Lopez E (2015). How rocky are they? The composition distribution of Kepler’s sub-Neptune planet candidates within 0.15 AU. Astrophys J.

[R398] Wong MH, Mahaffy PR, Atreya SK, Niemann HB, Owen TC (2004). Updated Galileo probe mass spectrometer measurements of carbon, oxygen, nitrogen, and sulfur on Jupiter. Icarus.

[R399] Wood PL, Maxted PFL, Smalley B, Iro N (2011). Transmission spectroscopy of the sodium ‘D’ doublet in WASP-17b with the VLT. Mon Not R Astron Soc.

[R400] Wordsworth R (2015). Atmospheric heat redistribution and collapse on tidally locked rocky planets. Astrophys J.

[R401] Wordsworth R, Forget F, Selsis F, Madeleine J-B, Millour E, Eymet V (2010). Is Gliese 581d habitable? Some constraints from radiative-convective climate modeling. Astron Astrophys.

[R402] Wu Y, Lithwick Y (2013). Density and eccentricity of Kepler planets. Astrophys J.

[R403] Wyttenbach A, Ehrenreich D, Lovis C, Udry S, Pepe F (2015). Spectrally resolved detection of sodium in the atmosphere of HD 189733b with the HARPS spectrograph. Astron Astrophys.

[R404] Yang J, Peltier WR (2012a). Y Hu, The initiation of modern soft and hard snowball Earth climates in ccsm4. Clim Past.

[R405] Yang J, Peltier WR, Hu Y (2012b). The initiation of modern soft snowball and hard snowball climates in ccsm3. part i: The influences of solar luminosity, co2 concentration, and the sea ice/snow albedo parameterization. J Climate.

[R406] Yang J, Peltier WR, Hu Y (2012c). The initiation of modern soft snowball and hard snowball climates in ccsm3. part ii: Climate dynamic feedbacks. J Climate.

[R407] Yang J, Cowan NB, Abbot DS (2013). Stabilizing cloud feedback dramatically expands the habitable zone of tidally locked planets. Astrophys J Lett.

[R408] Yang J, Liu Y, Hu Y, Abbot DS (2014). Water trapping on tidally locked terrestrial planets requires special conditions. Astrophys J Lett.

[R409] Yelle RV (2004). Aeronomy of extra-solar giant planets at small orbital distances. Icarus.

[R410] Yung YL, Allen M, Pinto JP (1984). Photochemistry of the atmosphere of Titan—Comparison between model and observations. Astrophys J Suppl Ser.

[R411] Zahnle K, Marley MS (2014). Methane, carbon monoxide, and ammonia in brown dwarfs and self-luminous giant planets. Astrophys J.

[R412] Zahnle K, Marley MS, Fortney JJ (2009a). Thermometric soots on warm Jupiters?. ArXiv e-prints.

[R413] Zahnle K, Marley MS, Freedman RS, Lodders K, Fortney JJ (2009b). Atmospheric sulfur photochemistry on hot Jupiters. Astrophys J Lett.

[R414] Zapatero Osorio MR, Béjar VJS, Martín EL, Rebolo R, Barrado y Navascués D, Bailer-Jones CAL, Mundt R (2000). Discovery of young, isolated planetary mass objects in the *σ* orionis star cluster. Science.

[R415] Zhang X, West RA, Banfield D, Yung YL (2013). Stratospheric aerosols on Jupiter from Cassini observations. Icarus.

[R416] Zhou G, Bayliss DDR (2012). Detection of sodium absorption in WASP-17b with Magellan. Mon Not R Astron Soc.

